# Identification and validation of a prognostic risk model based on caveolin family genes for breast *cancer*

**DOI:** 10.3389/fcell.2022.822187

**Published:** 2022-09-06

**Authors:** Qiang Tang, Shurui Wang, Ziyang Di, Huimin Li, Kailiang Xu, Xin Hu, Maojun Di

**Affiliations:** ^1^ Department of General Surgery, Shiyan Taihe Hospital, Hubei University of Medicine, Shiyan, China; ^2^ School of Nursing Peking Union Medical College, Beijing, China; ^3^ Department of Urology, Jingzhou Central Hospital, the Second Clinical Medical College, Yangtze University, Jingzhou, China

**Keywords:** breast cancer, caveolins, expression, prognosis, methylation, infiltrating immune, TMB

## Abstract

**Background:** Breast cancer (BC) is the most vicious killer of women’s health and is accompanied by increased incidence and mortality rates worldwide. Many studies have demonstrated that caveolins (CAVs) were abnormally expressed in a variety of tumors and implicated in tumorigenesis and cancer progression. However, the role of CAVs in BC remains somewhat contentious.

**Methods:** We comprehensively explored the expression and prognostic value of CAVs (CAV1-3) in BC utilizing public databases (ONCOMINE, TIMER, UALCAN, and TCGA databases). Then we constructed a prognostic model based on the expression profiles. Also, a prognostic nomogram was built to predict the overall survival (OS). We further investigated the relationship between this signature and immune cell infiltration and the mutational landscape in BC. The R package “pRRophetic” was used to predict chemotherapeutic response in BC patients. Finally, we employed loss-of-function approaches to validate the role of CAVs in BC.

**Results:** We found that CAVs were significantly downregulated in various cancer types, especially in BC. Low CAV expression was closely related to the malignant clinicopathological characteristics and worse OS and relapse-free survival (RFS) in BC. Then we constructed a prognostic model based on the expression profiles of CAVs, which divided BC patients into two risk groups. The Kaplan–Meier analysis showed that patients in the high-risk group tend to have a poorer prognosis than those in the low-risk group. Multivariate analysis indicated that the risk score and stage were both independent prognostic factors for BC patients, suggesting a complementary value. The clinical profiles and risk module were used to construct a nomogram that could accurately predict the OS in BC. In addition, we found that patients in the low-risk group tend to have a relatively high immune status and a lower mutation event frequency compared to the high-risk group. Furthermore, this signature could predict the response to chemotherapy and immunotherapy. Finally, CAV depletion promoted the colony formation, migration, and invasion of BC cells.

**Conclusion:** CAVs may serve as novel biomarkers and independent prognostic factors for BC patients. Also, the constructed signature based on CAVs may predict immunotherapeutic responses and provide a novel nomogram for precise outcome prediction of BC.

## Introduction

Breast cancer (BC) is the most common type of carcinoma and remains the first leading cause of cancer-related death in women around the world (Downs-Holmes and Silverman, 2011; Mascara and Constantinou, 2021). Although BC patients with early-stage and decent conditions can be cured by radical removal, chemotherapy, and targeting therapy, the prognosis of patients with metastasis, recurrence, and drug resistance is poor (Munagala et al., 2011; Wind and Holen, 2011). Therefore, the treatment of patients with these high-risk factors remains a great challenge for breast surgeons (Sledge et al., 2014). Currently, available molecular targeted therapy for BC has achieved some great success including estrogen receptor (ER)-targeting agents (e.g., tamoxifen) and human epidermal growth factor receptor 2 (HER2)-targeting therapeutics (e.g., trastuzumab) (Munagala et al., 2011; Liedtke and Kiesel, 2012; Miller, 2014). Some evidence has shown that the discovery and application of novel molecular biomarkers can provide prognostic value (Davis et al., 2020; Macklin et al., 2020). Therefore, the discovery and application of new diagnostic and prognostic molecular markers of early-stage tumors may provide new insights into the mechanisms of tumorigenesis and novel therapeutic targets.

Caveolae are flask-shaped vesicular organelles that are particularly abundant in the plasma membrane of cells (Scherer et al., 1994). Caveolins (CAVs) and cavins are the necessary structural proteins for the formation and fusion of the vesicle, which have been demonstrated to implicate in the transcytosis, potocytosis, and signal transduction of cancer cells (Gould et al., 2010; Parton and del Pozo, 2013; Nwosu et al., 2016). The caveolin protein family consists of three members in mammals: caveolin-1 (CAV1), caveolin-2 (CAV2), and caveolin-3 (CAV3) (Williams and Lisanti, 2004b). Previous studies showed that genes CAV1 and CAV2 lie adjacent to one another at chromosome region 7q31.1, and CAV3 locates on chromosome 3p25.3 (Engelman et al., 1998a; Engelman et al., 1998b; Aldred et al., 2003). The encoded protein, CAV1, and CAV2 can interact with each other and form a hetero-oligomeric complex, which constitutes the skeleton of the vesicle. Also, CAV3 has been confirmed to interact with CAV2 and CAV1 in an analogous fashion (Rybin et al., 2003; Pfleger et al., 2012). Recently, many studies have demonstrated that CAVs, especially CAV1, are abnormally expressed in a variety of tumors and implicated in tumorigenesis and cancer progression (Quest et al., 2008; El-Gendi et al., 2012; Lamaze and Torrino, 2015; Ketteler and Klein, 2018). However, the role of CAVs in cancer remains unclear and controversial (Ayala et al., 2013; Martinez-Outschoorn et al., 2015). CAV1 is downregulated in some cancer types, including lung cancer, colon cancer, ovarian carcinomas, and sarcomas (Bender et al., 2000; Wiechen et al., 2001; Quest et al., 2008). J A Engelman first reported that the expression of CAV1 was significantly suppressed in oncogene-transformed (H-Ras and v-Abl) fibroblasts cells and the overexpression of CAV1 could completely reverse the transformed phenotype and inhibit contact-dependent growth of fibroblasts cells (Koleske et al., 1995; Razandi et al., 2002). In contrast, some studies suggested that CAV1 was overexpressed in bladder, esophagus, lung, and prostate carcinomas (Yang et al., 1998; Yang et al., 1999; Ho et al., 2002; Kato et al., 2002; Yoo et al., 2003), and CAV1 upregulation could promote the proliferation, invasion, and distant metastatic potential of cancer cells (Joshi et al., 2008; Tanase et al., 2009; Martinez-Outschoorn et al., 2015). Additionally, patients with metastasis showed higher levels of CAV1 than those with non-metastasis in esophageal squamous cell carcinoma and renal cell carcinoma (Tahir et al., 2001; Kato et al., 2002). Therefore, the dual role of CAVs in cancer occurrence and metastasis generated many controversies regarding the exact function (tumor-suppressive or pro-oncogenic). The present study aimed to explore the expression pattern and prognostic value of CAVs. In addition, we integrated a CAV-based prognostic signature to predict the prognosis and immunotherapeutic response in BC.

## Materials and methods

### Identification of differential caveolin expression, promoter methylation, and genomic alterations in breast cancer

To explore the transcriptional expression of CAVs in various types of cancers, we analyzed genome-wide expression data from the Oncomine database ( *http://oncomine.org* ), which includes more than 400 unique analyses. Also , the expression in 33 cancer types of CAVs from TCGA data sets was analyzed for validation in TIMER ( *https://cistrome.shinyapps.io/timer/* ). The mRNA and promoter methylation levels of the CAVs in BC patients were downloaded from UALCAN ( *http://ualcan.path.uab.edu/analysis.html* ). Furthermore, we explored the relationship between CAV expression and the clinicopathologic parameters of BC patients. The Kaplan–Meier plotter ( *http://kmplot.com/analysis/index.php?P=service*&*cancer=breast* ) was applied to determine the association between the OS of patients with BC and the expression profiles of CAV genes, RFS and post progression survival (PPS). The copy number variation (CNV), mutations, and prognosis value of CAVs in BC were comprehensively evaluated according to the cBioPortal database ( *https://cbioportal.org* ), which included 10,920 samples involving 20 studies.

### Risk assessment model construction and prognostic survival analysis

We constructed a set of scoring systems to evaluate the CAV-relevant gene expression pattern of individual patients in BC. Then a principal component analysis (PCA) was performed to construct the CAV signature, which was termed as CAV score. Both principal components 1 and 2 were selected to act as signature scores. We defined the CAV score using a method similar to GGI (Sotiriou et al., 2006; Zeng et al., 2019): **CAV score = Σ (PC1i + PC2i)**, which is the expression of CAV-related genes.

### Evaluation of tumor-infiltrating immune cells between the high- and low-risk groups

To investigate whether the prognostic model could predict the immune response of BC patients, we analyze the associations between risk score and the proportion of different immune cells in the tumor microenvironment. The CIBERSORT algorithm was utilized to quantify the fraction of the relative 28 immune infiltration cell types in BC patients. Then we compared and analyzed the differential abundances of immune cell infiltration between the high- and low-risk groups via the Wilcoxon ranked-sum test.

### Prediction of chemotherapy and immunotherapy drug response

Genomics of Drug Sensitivity in Cancer database (GDSC), the largest integrated public pharmacogenomics database, can predict the chemotherapeutic response of patients with cancer and promote potential therapeutic applications of targeted agents in cancer treatment. The R package “pRRophetic” was used to predict the half-maximal inhibitory concentration (IC50) of chemotherapy drugs for the patients in high- and low-risk groups from the TCGA database. As previously mentioned, we further investigated the response to immunotherapy using a urothelial carcinoma cohort **(IMvigor210 cohort)**, which included 348 advanced bladder cancer patients treated with anti-PD-L1 antibody atezolizumab. The gene expression and clinical information data were extracted and analyzed with the R package. The response mainly included four outcome indices: complete response (CR), partial response (PR), progressive disease (PD), and stable disease (SD). Among these, patients with CR or PR were classified as responder groups and SD or PD were classified as non-responder groups. Then the difference between the responder and non-responder groups was analyzed with the Wilcoxon test.

### Gene set enrichment analysis

GeneMANIA could provide the genetic and protein interactions, co-expression, pathways, co-localization, and domain-protein similarity of the candidate genes. In this study, we performed a comprehensive analysis to identify the network between the interacting and co-expression proteins, and pathways of the CAVs. Metascape was used for comprehensive GO and KEGG function enrichment to identify and visualize the networks and enriched pathways of the CAVs.

### Cell lines and transfection

MDA-MB-231 cell lines were cultured in Dulbecco’s modified Eagle’s medium (DMEM) with 10% FBS (HyClone, United States) in a humidified atmosphere containing 5% CO_2_ and 95% O_2_ at 37°C. The specific CAVs shRNA and the control were purchased from Shanghai GenePharma (The sequences are shown in Supplementary Table S1) and were transfected into MDA-MB-231 cells with Lipo3000 kit according to the manufacturer’s protocol.

### Western blotting

Western blot (WB) analysis was performed as described previously. Equal amounts of protein (30–50 μg) were separated by 4–12% sodium dodecyl sulfate polyacrylamide gel electrophoresis (SDS/PAGE) and transferred to PVDF membranes. 5% non-fat milk was used for blocking purposes for 1 h. Then the membranes were incubated with the corresponding primary antibody [CAV1 (1:1,000 dilution, CST), CAV2 (1:1,000, Santa), CAV3 (1:1,000, Santa), GAPDH (1:1,000, Santa), Vimentin (1:1,000, Santa), E-cadherin (1:1,000, Santa), N-cadherin (1:1,000, Santa), AKT/p-AKT (1:1,000 dilution, CST), ERK/p-ERK (1:1,000 dilution, CST)] overnight at 4°. The appropriate second antibody was added and incubated for 1 h at room temperature. Then the bands were detected using a enhanced ECL western blotting kit.

### Wound healing and transwell assays

Cells were trypsinized and reseeded into 6-well dishes and incubated for 24 h. When cells reached more than 90% convergence, the wound was performed with a pipette tip. Then, cells were washed with PBS and replaced with a serum-free medium. Cells were photographed after 0 and 24 h in ×4 magnification, and the width of the wound was recorded.

Transwell assays were performed to determine the cell migration and invasion ability. 1 × 10^5^ cells were seeded in the upper chamber with a serum-free medium (the upper chamber was covered with Matrigel in the invasion assay), and a medium with 10% FBS was added into the lower chamber (600 ul). After incubating for 24h, the cell on the outer membrane was fixed and stained with 0.1% crystal violet solution. The migrated and invalided cells crossed the polycarbonate membrane and were counted under a light microscope.

### Colony and sphere formation experiments

A clone formation assay was performed to estimate the capacity of cell proliferation *in vitro*. A total of 500–1,000 cells were cultured in a six-well plate for 7–14 days and then fixed with 4% paraformaldehyde and stained with 0.5% crystal violet for 15 min. The number of colonies was counted, and the plate clone formation efficiency was calculated.

To investigate the effect of CAVs on the stemness of breast cancer cells, we performed a sphere formation assay with CAV knockdown cells. MDA-MB-231 cells (4,000–5,000 cells) were cultured in non-adherent culture plates supplemented with EGF, FGF, and B27 complement for 7–14 days, with media changes every 3–4 days. The number of tumor spheres was counted, and the morphology was observed under a light microscope.

### Statistical analysis

GraphPad software version 8.0, R (4.0.0) software, and SPSS 23 software were used for the statistical analysis. Statistical differences between different groups were calculated by Student’s t-test. Kaplan–Meier analysis was used to estimate survival, and the difference was compared by log-rank test. *p* < 0.05 was defined as statistically significant.

## Results

### Differential expression level of caveolins in breast cancer

**FIGURE 1:**
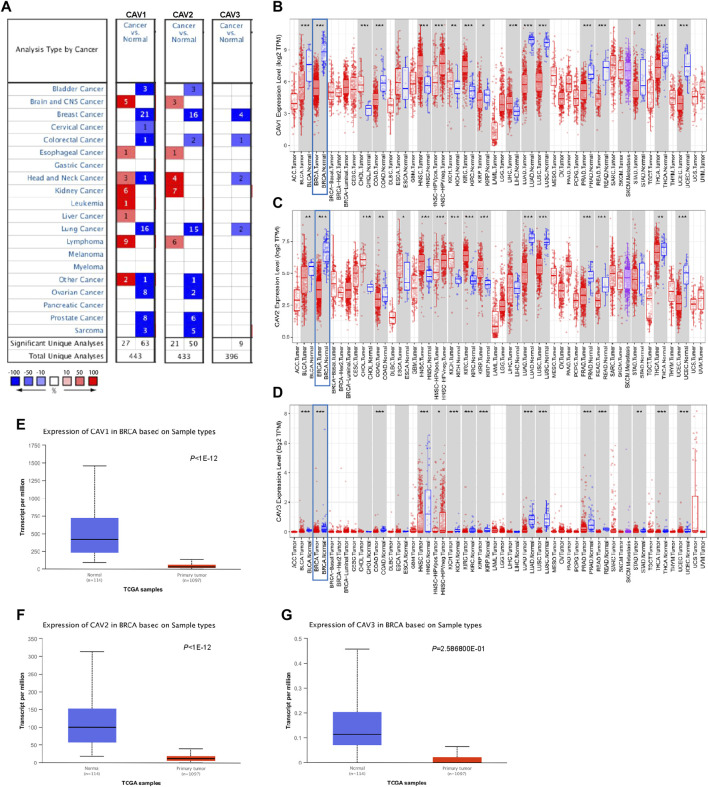
Expression of CAV family members in tumors. **(A)** Transcription levels of CAVs in different human tumor types (Oncomine database, http://oncomine.org). **(B–D)** Decreased expression of CAVs in 33 tumor types. Red, tumor samples; gray, normal samples (TIMER database, http://timer.comp-genomics.org/). **(E–G)** CAVs were expressed low in BC tissues compared with normal tissues (UALCAN database, http://ualcan.path.uab.edu/).

A meta-analysis was performed to detect the expression levels of CAVs in various cancer types using the Oncomine database. We found a total of 444, 433, and 396 unique analyses for CAV1, CAV2, and CAV3, respectively. Among the significant analyses, 70% of the study revealed that CAV1 and CAV2 were all significantly downregulated in most tumor types, especially in BC, lung cancer, ovarian cancer, prostate cancer, bladder cancer, and sarcoma cancer. Also, all nine included significant studies which presented that CAV3 was expressed low in tumor tissues when compared to non-tumor tissues (Figure 1A). We further explored the expression levels of CAVs in another independent data set (TIMER database) and validated that CAV1, CAV2, and CAV3 were expressed low in most cancer, especially in BC (Figures 1B–D). In addition, consistent results were also obtained in the UALCAN database (http://ualcan.path.uab.edu/), which suggested that the protein levels of CAVs were significantly reduced in the breast cancer tissues compared with the adjacent non-tumor tissues (Figures 1E–F). Overall, by analysis of these publicly available databases, we found that CAVs were downregulated in BC tissues.

### Prognostic value of caveolins in breast cancer

**FIGURE 2:**
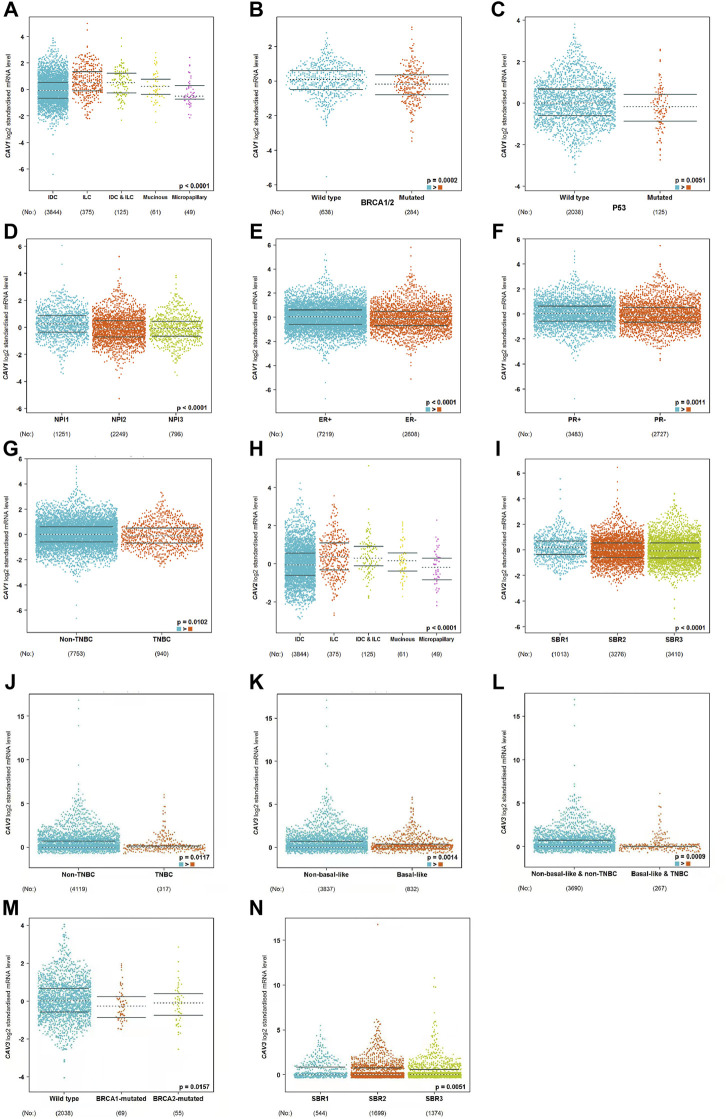
**(A–N)** Analysis of the relationship between CAV expression and the clinicopathologic parameters in BC according to the bc-GenExMiner v4.8 database (http://bcgenex.ico.unicancer.fr/BC-GEM/GEM-Accueil.php?js=1).

**FIGURE 3:**
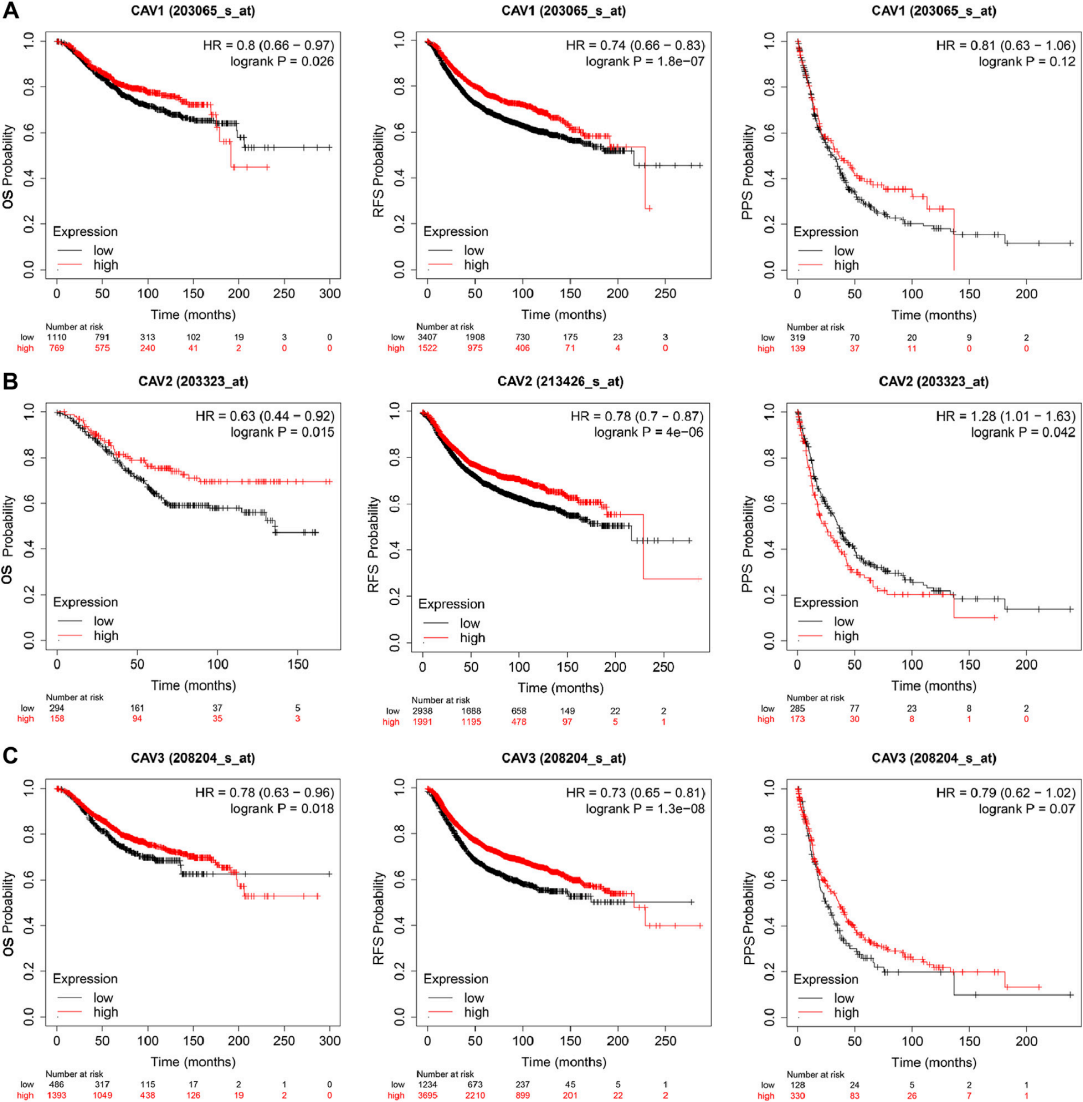
Kaplan–Meier survival curve analysis **(**OS, RFS, PPS) for CAVs in BC (Kaplan–Meier plotter, https://kmplot.com/analysis/): **(A)** CAV1, **(B)** CAV2, and **(C)** CAV3.

To determine the prognostic value of CAVs in BC, we explored the association between the expression of CAVs and tumor pathological features. The results demonstrated that lower expression of CAV1 was significantly correlated with history subtypes (Figure 2A), BRCA1/2 mutation (Figure 2B), TP53 mutation status (Figure 2C), and Nottingham prognostic index (NPI) value (Figure 2D). Also, the expression of CAV1 was significantly decreased in TNBC patients compared with non-TNBC patients (Figures 2E–G). CAV2 expression was significantly associated with history subtypes and Scarff-Bloom-Richardson (SBR) grade (Figures 2H,I). The expression of CAV3 in TNBC and basal-like cell carcinoma patients was significantly lower than that in the non-TNBC and non-basal-like cancer patients (Figures 2J–L). In addition, lower expression of CAV3 was significantly correlated with a BRCA1/2 mutation (Figure 2M) and SBR value (Figure 2N). These results demonstrated that CAV expression was associated with adverse pathologic outcomes in BC. Furthermore, the survival values of the CAVs were generated by the Kaplan–Meier (KM) plotter, which suggested that the survival time of patients in the low-expression CAV group was significantly shorter than that in the high-expression group (Figures 3A–C). Specifically, low expressions of CAV1, CAV2, and CAV3 were closely related to poor overall survival (OS) and relapse-free survival (RFS) (all *p* < 0.05). These results were validated by the Breast *Cancer* Gene-Expression Miner v4.7 database (http://bcgenex.centregauducheau.fr/BC-GEM/GEM-Accueil.php?js=1) (Supplementary Figures 1A–C). These results indicated that low CAV expression predicts an unfavorable prognosis in BC patients.

### Correlation between deoxyribonucleic acid methylation of caveolins and prognosis of patients with breast cancer

**FIGURE 4:**
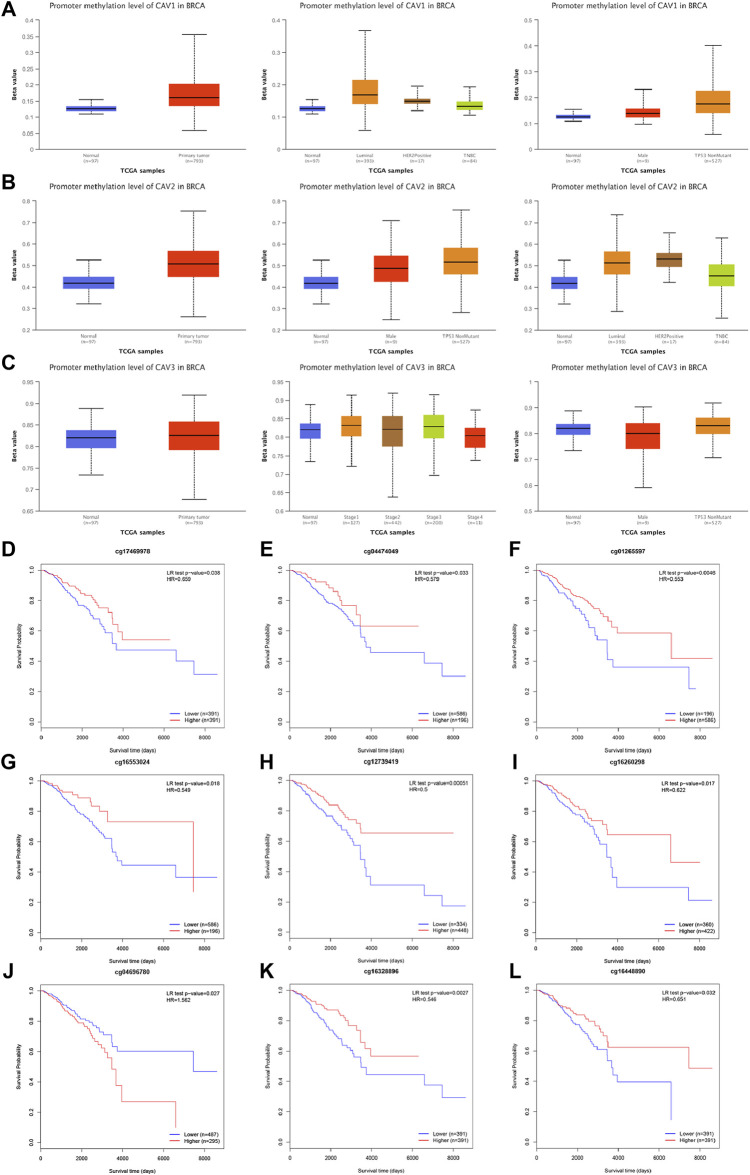
DNA methylation level of CAV promoters correlated with the prognosis of BC. **(A–C)** CAV1 and CAV2 were hypermethylated in BC tissues. **(D–L)** Prognostic value of CAVs –specific CpG site methylation in BC.

A growing body of research suggests that DNA methylation at active gene elements can directly modulate gene expression and involve in carcinogenesis and tumorigenesis. Methylation of the gene promoter has been considered an important mechanism regulating gene transcription. Therefore, we investigated whether CAV methylation is related to the prognosis of BC and found that the methylation levels in the CAV1 and CAV2 promoters were markedly higher than those in the corresponding para-cancerous tissues (Figures 4A–C). These results revealed that promoter hypermethylation might induce downregulation of CAV1 and CAV2 expression. Furthermore, we also found that DNA methylation levels of CAV1 and CAV2 were associated with TP53 status and history types (Figures 4A,B). The prognostic impact of DNA methylation of CAVs in BC was analyzed by MethSurv. The results suggested that four CpG sites of CAV1 (cg01265597, cg18329,349, cg17469,978, and cg04474049), four CpG sites of CAV2 (cg12739419, cg16260298, cg04696780, and cg16553024), and two CpG sites of CAV3 (cg16328896 and cg16448890) showed an association with poor prognosis (Table 1). Then, the KM survival curve was performed and suggested that all these critical CpG sites were associated with OS of patients with BC (Figures 4D–L).

**TABLE 1:** Significant prognostic values of CpG in the CAV family members.

Gene symbol	CpG Name	Hazard ratio	CI	*p* value	UCSC RefGene Group	Relation to UCSC CpG Island
CAV1	cg01265597	0.553	(0.371; 0.823)	0.0046	TSS1500	N_Shore
	cg18329349	0.597	(0.394; 0.906)	0.019	5'UTR;1stExon	Island
	cg17469978	0.659	(0.442; 0.982)	0.038	TSS200	Island
	cg04474049	0.579	(0.339; 0.988)	0.033	TSS1500	N_Shore
CAV2	cg12739419	0.5	(0.336; 0.743)	0.00051	Body	S_Shore
	cg16260298	0.622	(0.419; 0.922)	0.017	Body	Island
	cg04696780	1.562	(1.055; 2.312)	0.027	TSS200	N_Shore
	cg16553024	0.549	(0.322; 0.936)	0.018	TSS1500	N_Shore
CAV3	cg16328896	0.546	(0.365; 0.817)	0.0027	TSS200	Open_Sea
	cg16448890	0.651	(0.438; 0.968)	0.032	Body	Open_Sea

### Expression of caveolins in breast cancer cell lines and tissues

**FIGURE 5:**
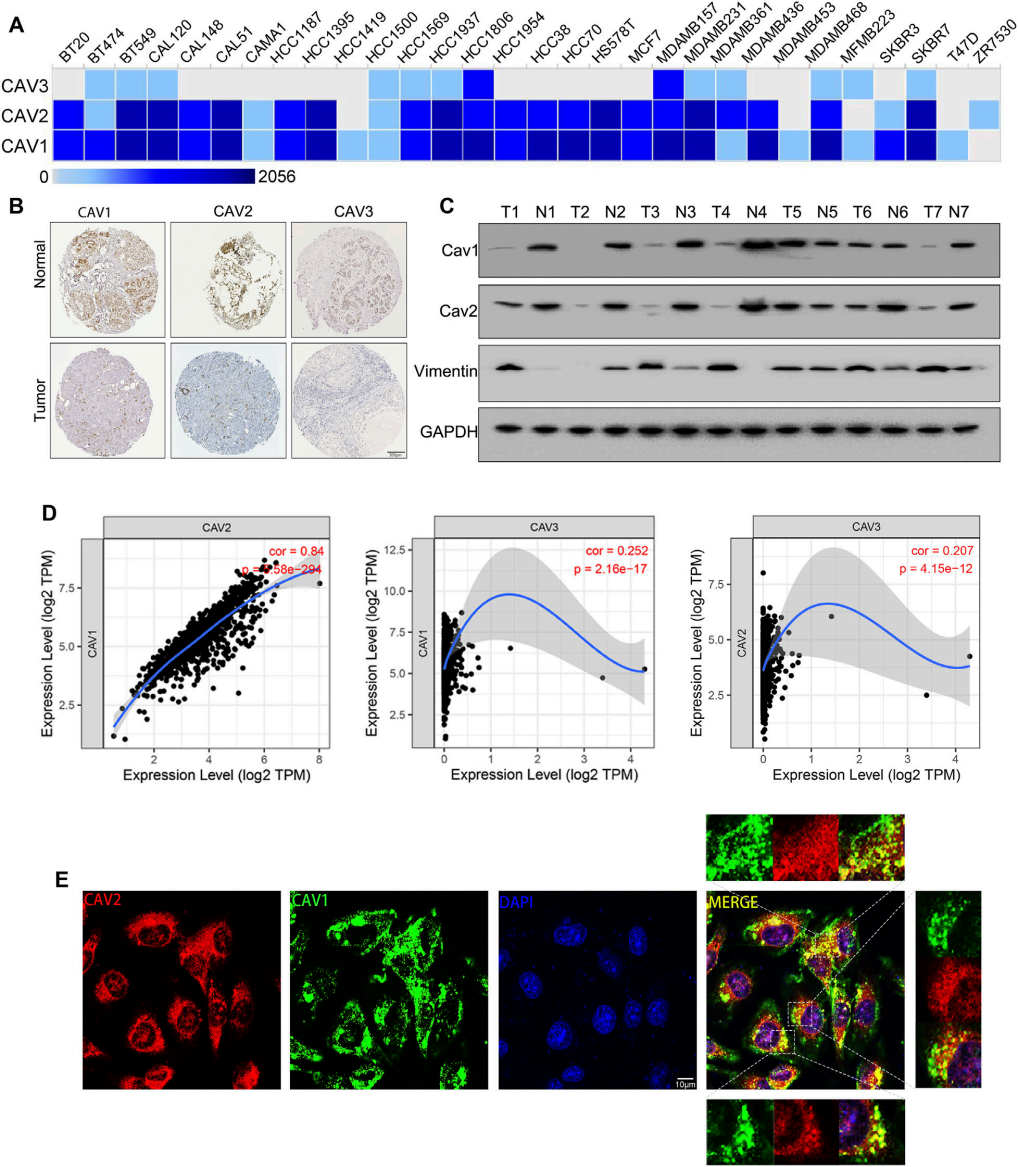
Expression of CAVs in BC cell lines and tissues. **(A)** Transcription expression of CAVs in 30 types of BC cancer cells (EMBL-EBI database, https://www.ebi.ac.uk/). **(B)** Immunohistochemistry showed the protein expression of CAVs in BC. **(C)** WB analysis of the expression of CAV1, CAV2, and Vimentin in BC tissues. **(D)** Correlation analysis between CAV expressions in BC. **(E)** Double immunofluorescence staining displayed the co-localization of CAV1 and CAV2.

We next evaluated the expression of CAVs in BC cell lines and tissues. According to the EMBL results, we found that CAV1 and CAV2 were moderately expressed in most BC cell lines. However, CAV3 was expressed in a portion of BC cells (Figure 5A). In addition, we investigated the expression of CAVs in BC tissues in the HPA database, and the results confirmed that CAV1, CAV2, and CAV3 were expressed low in BC tissues compared to that in normal tissues. Particularly, the expressions of CAV1 and CAV2 were significantly downregulated in BC tissues (Figure 5B). Then these results were validated by WB analysis, which also demonstrated that the expression of CAV1 and CAV2 was negatively correlated with that of Vimentin (Figure 5C). In addition, we explored the correlation between expression profiles of CAVs with GEPIA2 (http://gepia2.cancer-pku.cn/). As shown in Figure 5D, the expression of CAV1 precisely paralleled that of CAV2. Furthermore, we evaluated the co-localization of endogenous CAV1 with CAV2, which suggested that CAV1 and CAV2 had an obvious distribution in the cell cytoplasm, membrane, and nucleus, and co-localization of CAV1 and CAV2 was found in the cell cytoplasm (Figure 5E).

### Risk assessment model construction and prognostic survival analysis

**FIGURE 6:**
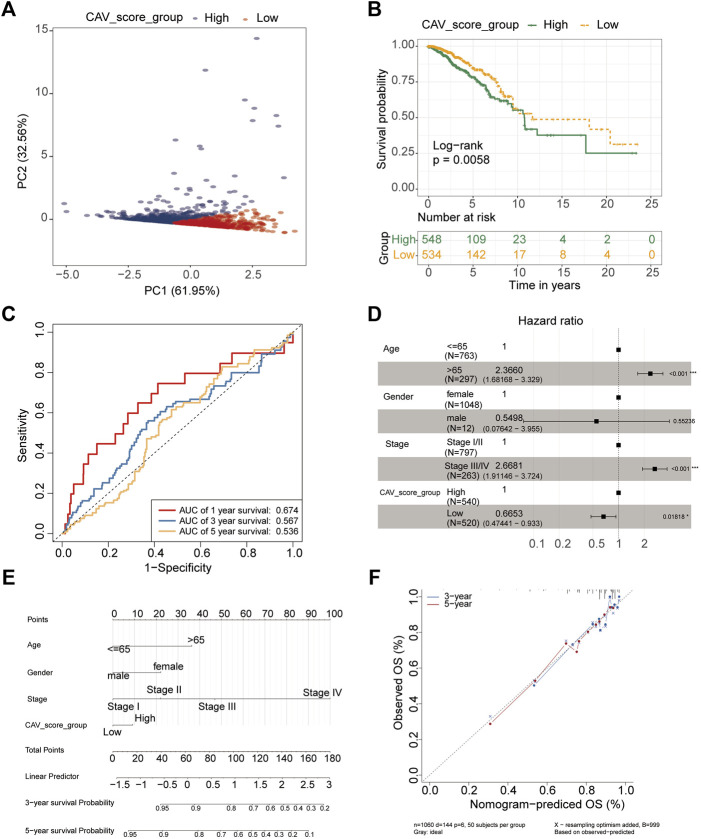
Construction and validation of the prognostic model based on CAV genes in BC. **(A)** PCA showing the two clusters of samples (PC1 and PC2). **(B)** Kaplan–Meier survival curves of OS between the high- and low-risk groups. **(C)** Survival ROC curves for predicting OS of BC patients by the risk score. **(D)** Forest plot representation of the multivariate Cox regression analysis of the risk score and clinical features for BC. **(E)** Nomogram consists of the clinical features and risk scores of patients for predicting OS. **(F)** Calibration curves of the nomogram to predict OS at 3 and 5 years.

As each of the CAVs had a good predictive value, we tried to construct a multigene model to evaluate the prognosis of BC. The results revealed that all patients included in the study could be divided into low-risk groups and high-risk groups depending on the risk score (Figure 6A), and patients in high-risk groups had a lower survival probability and patients in low-risk groups had a higher survival probability (Figure 6B). To validate the predictive value of risk scores, the receiver operating characteristic (ROC) curve was created and the areas under the curve (AUCs) for 1-, 3-, and 5-year survival were 0.674, 0.567, and 0.536, respectively (Figure 6C). Multivariate Cox regression analysis demonstrated that TNM stage (HR = 2.67, 95% CI = 1.91–3.72, *p* < 0.001) and prognosis models (HR = 2.67, 95% CI = 0.47–0.93, *p* < 0.05) were independent predictors of prognosis for BC patients (Figure 6D). Moreover, a nomogram was conducted using the TCGA data set based on the independent factors (age, gender, stage, and risk score). The calibration plots for the 3- and 5-year OS were predicted well in the TCGA cohort (Figures 6E,F). To further verify the reliability of the model, we downloaded another BRCA cohort as a validation data set from the GEO database (GSE21653). Then survival analysis was performed via Kaplan–Meier survival analysis, with differences between curves analyzed via a log-rank test. We found that the DFS in the high-risk signature group was significantly shorter than that in the low-risk signature group. ROC curve of 1-year, 3-year and 5-year survival were plotted in Supplementary Figures 1D,E.

### Risk signature was associated with immune cell infiltration and the mutational landscape of breast cancer patients

**FIGURE 7:**
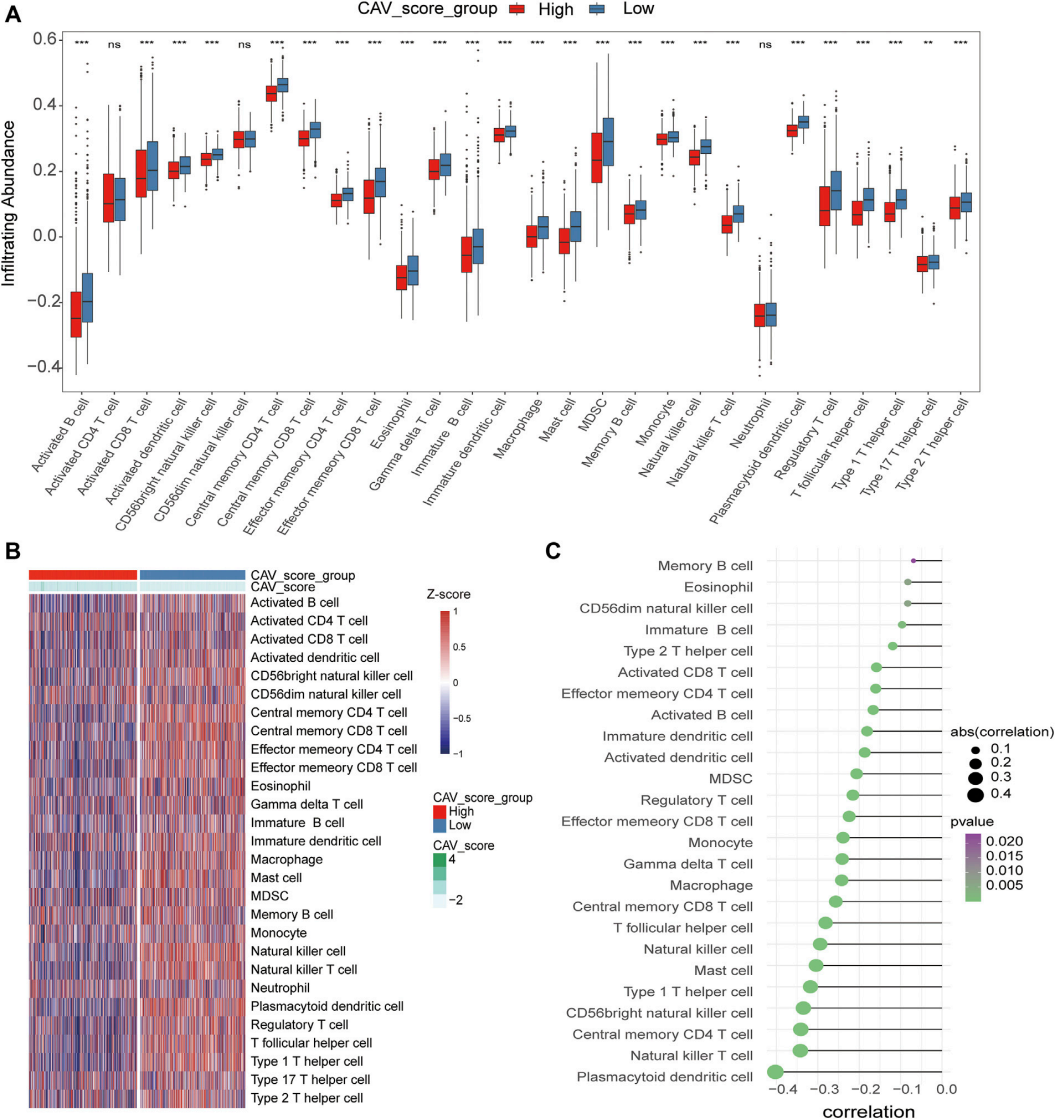
Correlations of CAVs with immune infiltration level in BC. **(A,B)** Identification of the relative infiltration of 28 types of immune cell subpopulations in high- and low-risk signature subgroups. **(C)** Correlation between immune infiltration cells and the risk score.

**FIGURE 8:**
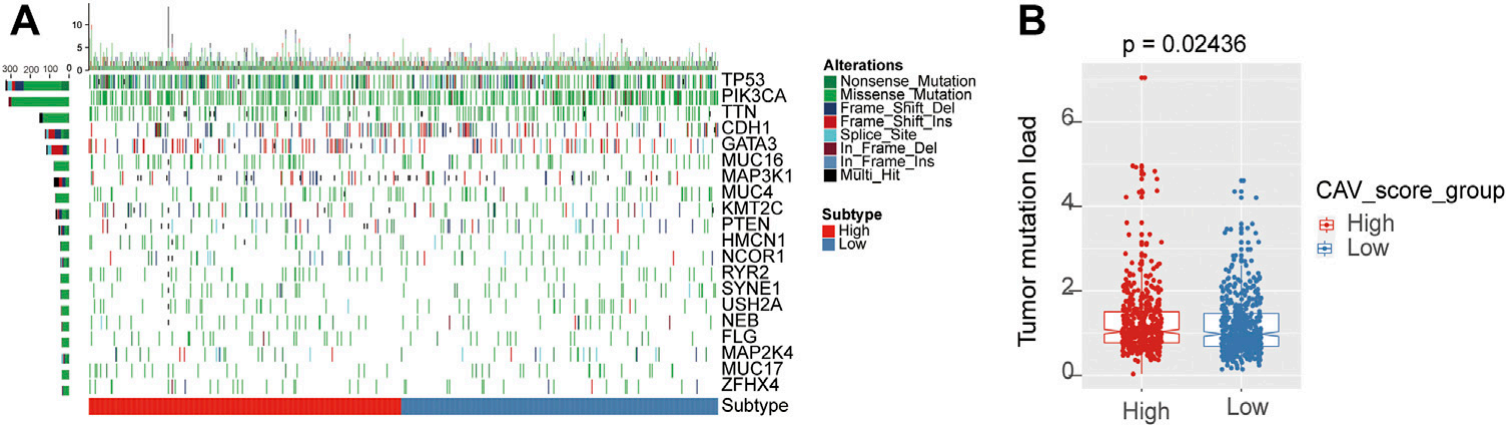
Differential landscape of tumor mutation burden between high- and low-risk groups. **(A)** Mutational landscape showed the frequency of mutated genes in high- and low-risk signature groups. **(B)** Tumor burden was quantitated by the Wilcoxon test in the risk groups.

To evaluate the relationship between the risk score and immune microenvironment, we performed CIBERSORT analysis to quantify the proportions of diverse immune cell subpopulations. We found that most immune cells are significantly different between the high- and low-risk groups. Moreover, patients in the low-risk group tend to have a relatively high immune status compared to the high-risk group (Figures 7A,B). Moreover, we found that the most noticeable correlations are the correlations between the risk score and plasmacytoid dendritic cell, natural killer T-cell, central memory CD4 T-cell, and CD56 bright natural killer cell (Figure 7C). Thus, we believed that BC patients with different phenotypes of the risk scores may directly lead to different immune statuses and subsequently result in diverse outcomes. We further explored the specific mutational landscape between the high- and low-risk groups. The waterfall plots were drawn and revealed that the mutation event frequency was significantly higher in the high-risk group than in the low-risk group. Also, the top 10 detected genetic mutations were APC, PI3CA, TTN, CDH1, GATA3, MUK16, MAP3K1, MUK4, KMT2C, and PTEN mutations (Figure 8A). Furthermore, the tumor mutation load was figured out, which suggested that the tumor mutation load in the high-risk group was significantly higher than those in the low-risk group (*p* < 0.05) (Figure 8B). Therefore, all these results suggested that tumor mutation load might be another risk factor for BC patients.

### Risk signature can predict the response to chemotherapy and immunotherapy in breast cancer

**FIGURE 9:**
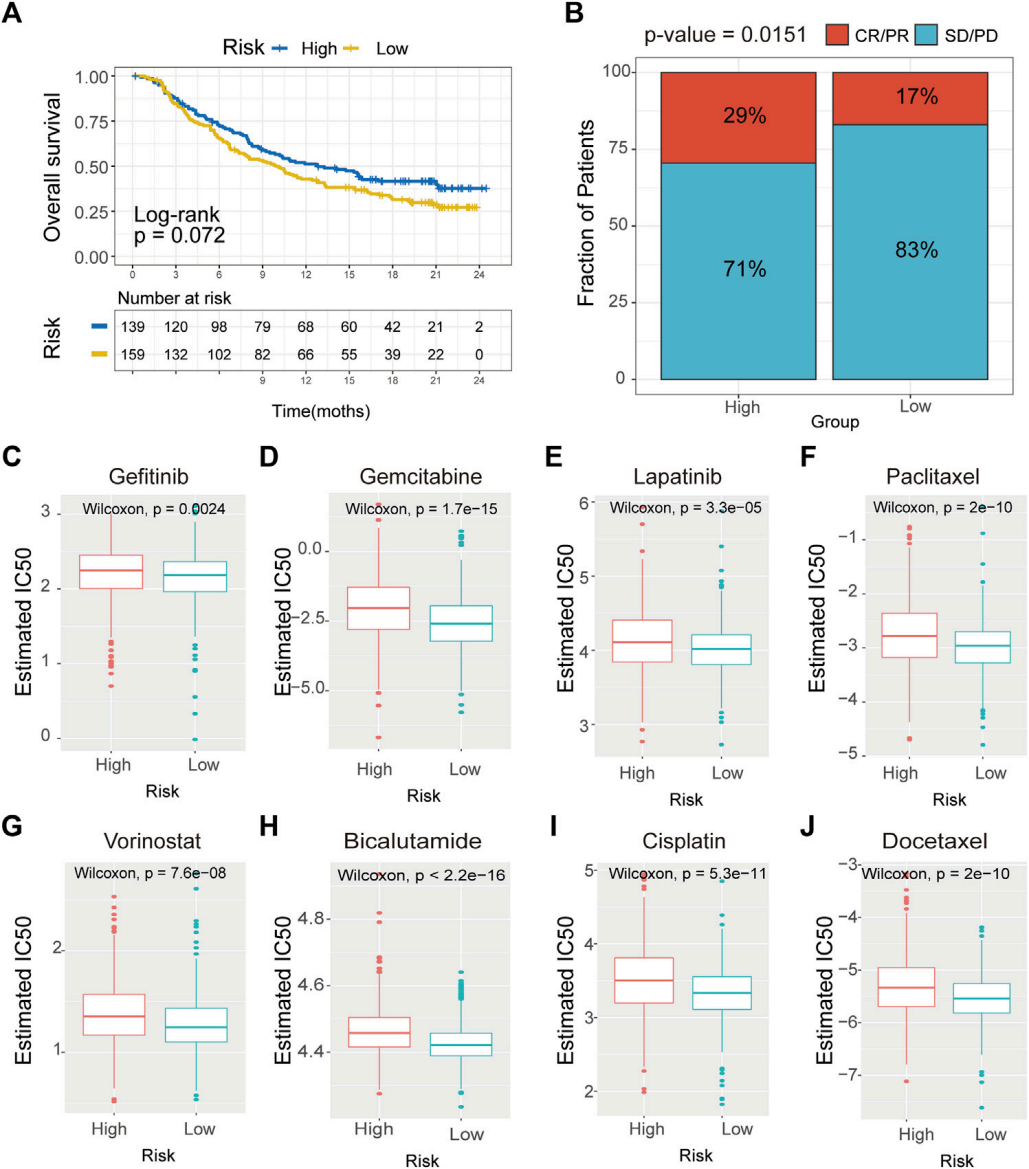
Drug sensitivity analysis of CAVs according to the prediction module. **(A)** Survival analysis of the drug responsiveness between the high- and low-risk subgroups according to the anti-PD-L1 cohort (IMvigor210 cohort). **(B)** Proportion of immune response to anti-PD-L1 treatment in high- and low-immune-risk score subgroups. **(C–J)** IC50 values for anticancer agents were compared between the high- and low-risk subgroups.

**FIGURE 10:**
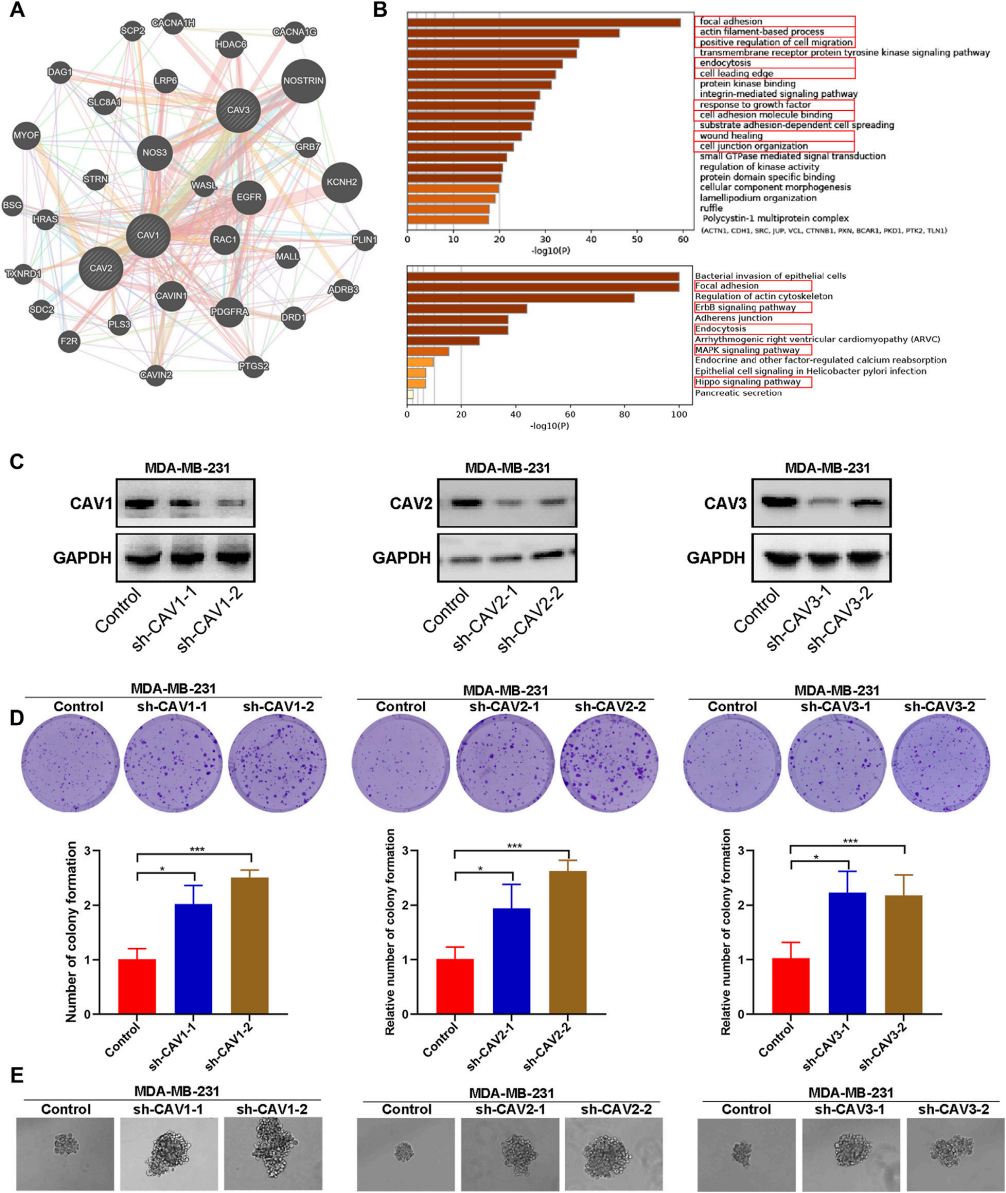
Functional enrichment, colony, and sphere formation analysis of CAVs in BC. **(A,B)** GO and KEGG pathways and functions analysis of CAVs and the enriched gene in BC. **(C)** The efficiency of CAV silencing was confirmed by WB. **(D)** Colony formation ability was determined by using a colony formation assay. **(E)** Quantification of the sphere formation ability of CAV-transfected MDA-MB-231 cells. *p < 0.05, **p < 0.01, ***p < 0.001.

Recently, it has been demonstrated that tumor immunogenicity, tumor mutation load, and immune infiltration in the tumor microenvironment had significant relationships with checkpoint blockade therapy, especially the anti-PD-1/L1 therapy. Therefore, we investigated whether this signature could predict the responsiveness of the patient to the immunotherapeutic treatment. We collated the clinical characteristics and outcomes of the patients treated with the anti-PDL1 agent from an immunotherapy cohort (Imvigor210). The results verified that patients in the high-risk group might benefit more from the immunotherapeutic treatment than those in the low-risk group (Figure 9A). Also, the cumulative remission and partial remission rates were significantly higher in the high-risk group than in the low-risk group (Figure 9B). Additionally, we also estimated the responsiveness of chemotherapy drugs in the high- and low-risk groups using the R package “pRRophetic”. The results suggested that the IC50 values of gefitinib, gemcitabine, lapatinib, paclitaxel, vorinostat, bicalutamide, cisplatin, and docetaxel in the low-risk group were significantly higher compared with the control group (Figures 10C–J). These results demonstrated that patients in the high-risk group were relatively sensitive to these agents.

### Enrichment analysis of caveolins in breast cancer patients

To further identify the biological function, the protein–protein interactions (PPIs), and co-expressed genes of the signature genes, we constructed a network using GeneMANIA Database and Metascape. A PPI network was established, and the network of enriched terms was labeled with different colors (Figure 10A). We performed GO and KEGG pathway analysis of network genes using the Metascape. As shown in Figure 10B, these genes were particularly enriched in GO:0,005,925 focal adhesion, the GO:0,030,029 actin filament-based process, GO:0,030,335 positive regulation of cell migration, GO:0,006,897 endocytosis, GO:0,031,252 cell leading edge, GO:0,070,848 response to growth factor, GO:0,050,839 cell adhesion molecule binding, GO:0,034,446 substrate adhesion-dependent cell spreading; GO:0,042,060 wound healing; GO:0,034,330 cell junction organization; hsa04510 Focal adhesion, hsa04810 regulation of the actin cytoskeleton, the hsa04012 ErbB signaling pathway, hsa04520 Adherens junction, hsa04144 endocytosis, the hsa04010 MAPK signaling pathway, ko04961 endocrine and other factor-regulated calcium reabsorption, and hsa05120 epithelial cell signaling in *Helicobacter pylori* infection. Therefore, these genes mainly participated in cell adhesion, migration, endocytosis, and the ErbB signaling pathway.

### Caveolin downregulation promoted colony and tumor sphere formation of breast cancer cells *in vitro*

To explore the effect of CAVs on the BC cells, we constructed short hairpin RNA (shRNA) pools specifically targeting CAV1, CAV2, and CAV3 in MDA-MB-231 cells. The efficiency of CAV silencing was confirmed by WB (Figure 10C). Colony formation and sphere formation assays were used to investigate the tumorigenesis potential of the transfected cells. The results revealed that CAV depletion dramatically increased the clone formation and sphere formation capability (Figures 10D,E). Therefore, CAVs may have a significant effect on the growth and stemness maintenance of BC cells.

### Caveolin downregulation enhanced migration and invasion of breast cancer cells

**FIGURE 11:**
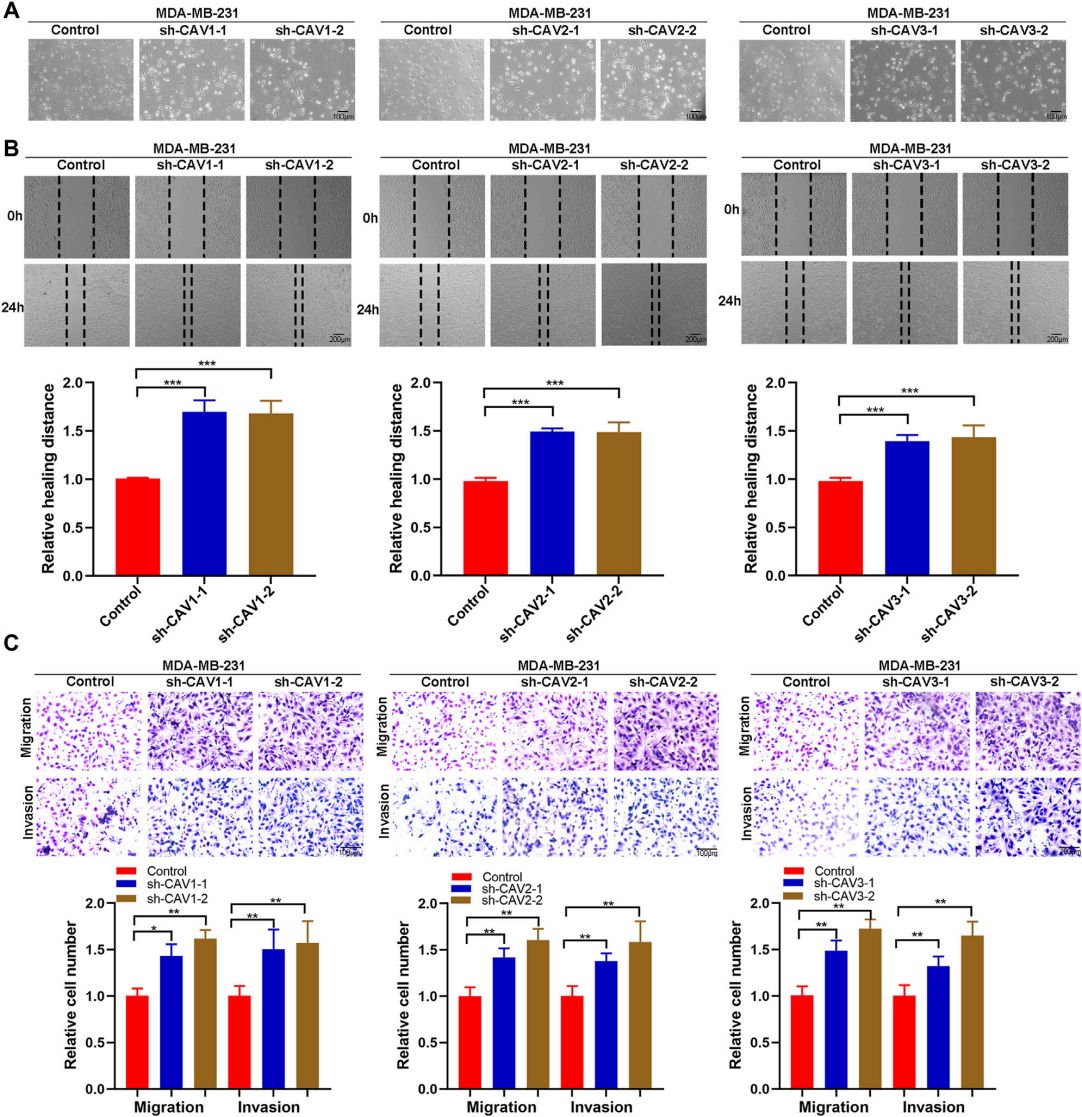
CAVs modulated migration and invasion of BC cells. **(A)** Wound healing assay measuring the cell migration capability of the indicated BC cells. **(B,C)** Transwell assay showed the migration and invasion ability of MDA-MB-231 cells. *p < 0.05, **p < 0.01, ***p < 0.001.

**FIGURE 12:**
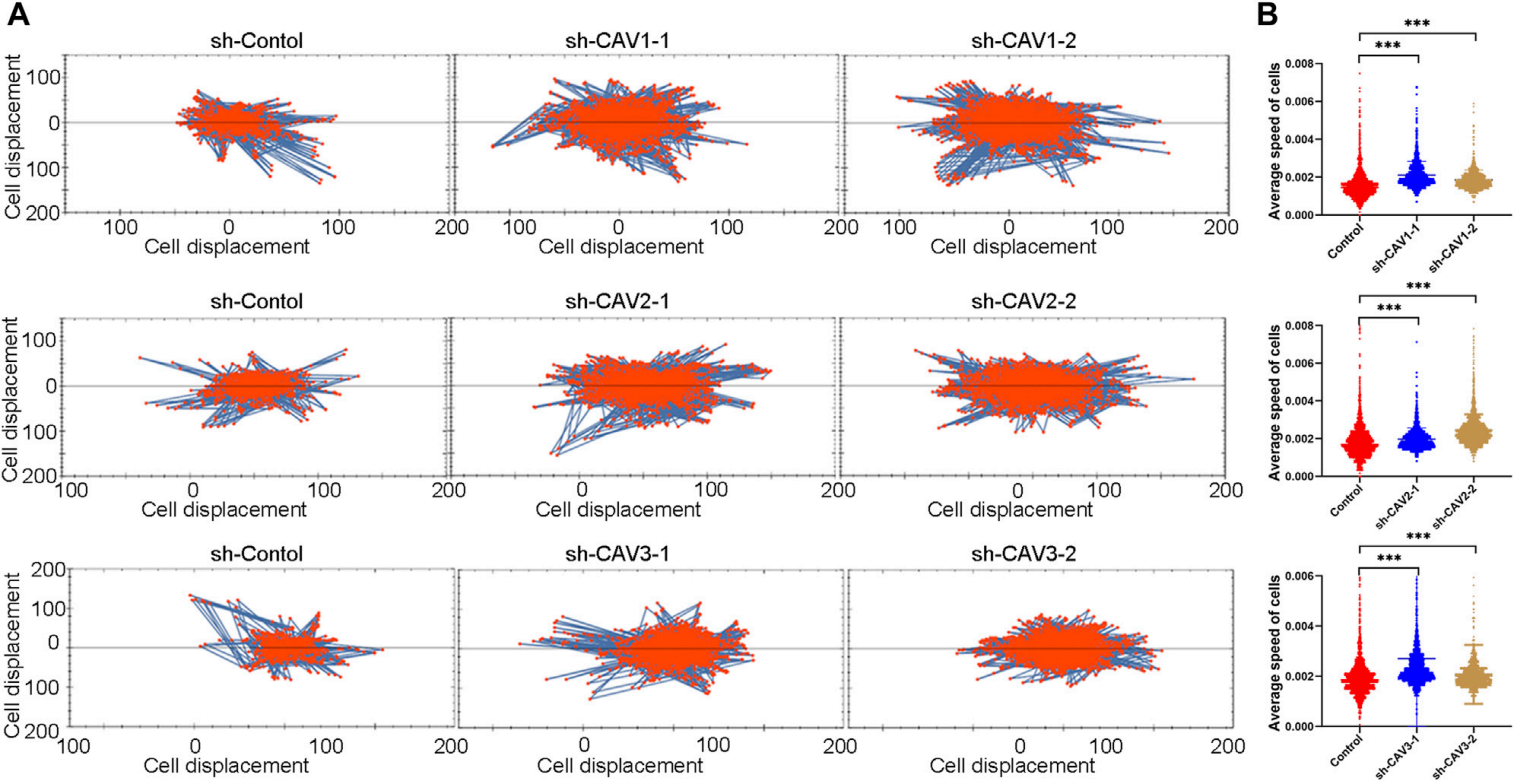
Cell motility was monitored in real time by The High-Throughput Connotation of Imaging System. **(A)** CAV knockdown increased the cumulative displacement **(B)** and increased the mean rate of motion of indicated cells. *p < 0.05, **p < 0.01, ***p < 0.001.

It has been demonstrated that alterations in morphology from the epithelial to mesenchymal phenotype are the most prevalent features of epithelial mesenchymal transition (EMT). In this study, CAV knockdown induced morphological changes from the cobble stone-like appearance to the elongated, spindle-like mesenchymal shape (Figure 11A). Then, we detected the cell migration and invasion ability in BC cells through wound healing and Transwell assay. According to the wound healing assay, we found that CAV1, CAV2, and CAV3 knockdown could significantly increase the migration of MDA-MB-231 cells (Figure 11B). Similar results were also observed in the Transwell assay migration experiments. In addition, the Transwell assay also revealed that CAV silencing could enhance the invasive capability of BC cells (Figure 11C). Moreover, real-time live-cell migration observation was performed using the High-Throughput Connotation of Imaging System, and the results suggested that CAV depletion could increase the cumulative displacement of BC cells, and the CAV knockdown cells moved significantly faster on average than the control cells (Figures 12A,B).

### CAV1 and CAV2 participate in the process of epidermal growth factor receptor endocytosis

**FIGURE 13:**
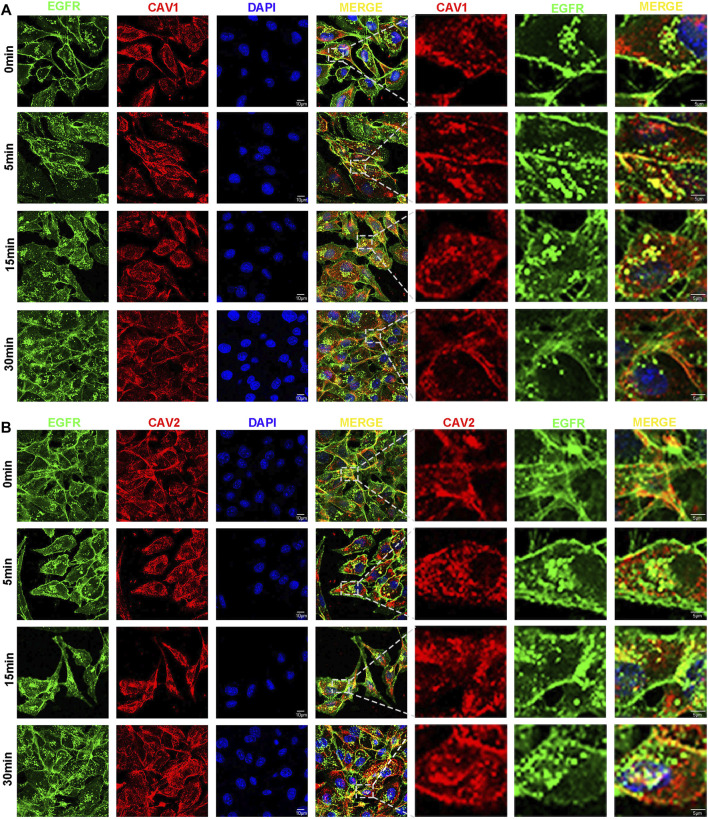
CAVs participate in the process of EGFR endocytosis. **(A,B)** After serum starvation for 12h, cells were stimulated with EGF (50 ng/ml) for 0, 5, 15, and 30 min. The cells were fixed and blocked, and primary and secondary antibodies were applied (EGFR stained with green, CAV1 **(A)** and CAV2 **(B)** with red, and nuclei with DAPI).

It has been demonstrated that epidermal growth factor receptor (EGFR) signaling involves multiple biological possession of carcinoma, including cell proliferation, migration, and survival (An et al., 2018). EGFR endocytosis and trafficking are critical physiological processes that can modulate EGFR and the downstream signaling pathway through multiple mechanisms (An et al., 2018). In this study, the GO and KEGG function analysis revealed CAVs and the interacting genes/proteins were involved in the receptor endocytosis process, particularly EGFR. Therefore, we performed immunofluorescence to investigate whether CAV1 and CAV2 could regulate EGF-mediated endocytosis trafficking. As shown in Figures 13A,B, the results of the immunofluorescence assay demonstrated that CAV1 and CAV2 mainly coexisted with EGFR in the cytoplasmic membrane at the rest state; when supplemented with EGF, CAV1, and CAV2, they could co-localize with EGFR in the cytoplasm in a time-dependent manner. After 30 min incubation, the co-localization gradually decreased. Therefore, we speculated that CAV1 and CAV2 might be involved in EGF-mediated endocytosis trafficking of EGFR.

### Caveolins regulate EMT and MAPK signal pathways

**FIGURE 14:**
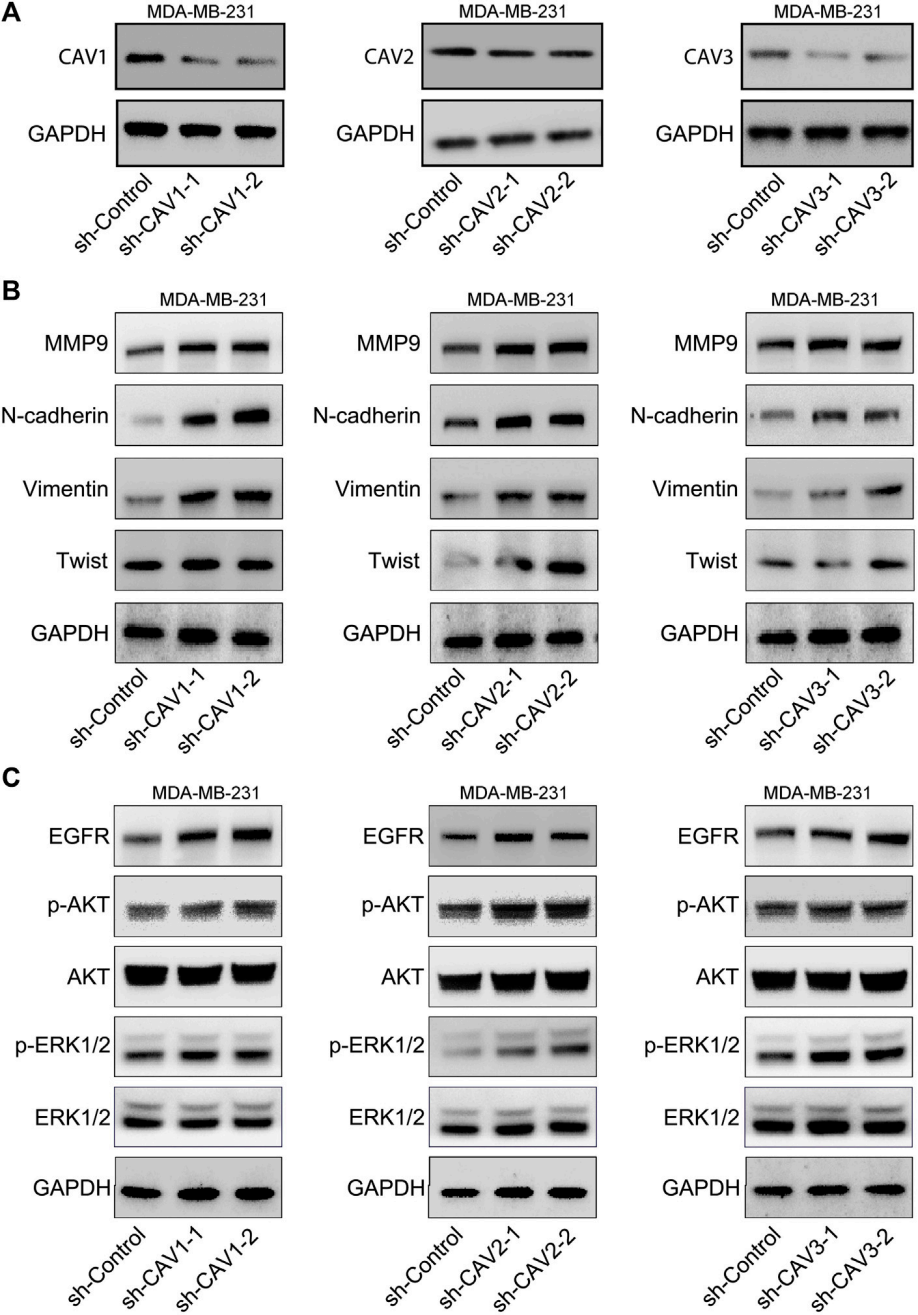
CAVs modulated EMT-associated genes and the MAPK and ERK pathways. **(A,B)** WB analysis detected the expression of Vimentin, N-cadherin, Twist, and MMP9. **(C)** MAPK and ERK signaling pathway-related proteins were detected using western blotting.

We detected the expression of the typical signs of EMT, N-cadherin, Vimentin, and MMP9 in CAV knockdown MDA-MB-231 cells. WB analysis suggested that CAV silencing significantly increased the expression of Vimentin, N-cadherin, MMP9, Twist, and Snail/Slug (Figures 14A,B). We further selected MAPK and ErbB pathways of the enrichment analysis of CAVs for validation. WB analyses showed that CAV knockdown could upregulate the expression of EGFR and subsequently increase the expression of the phosphorylation of ERK, AKT, and PI3K. These results demonstrated that CAVs could modulate EMT via MAPK and EGFR signal pathways in BC (Figure 14C).

## Discussion

Multiple studies have confirmed that the abnormal expression or function of caveolae due to somatic mutation or epigenetic regulation in CAVs was involved in numerous types of human diseases, including cardiovascular disease, muscular dystrophies, primary osteoporosis, pathogen infection, and tumors (Williams and Lisanti, 2004a; Lamaze and Torrino, 2015). Also, it suggested that CAVs could be involved in multiple biological processes of BC, such as tumor proliferation, migration, invasion, metastasis, and chemotherapy resistance (Ketteler and Klein, 2018). However, the relationship between CAVs and tumorigenesis remains contentious, especially for CAV1. Recently, some studies believe that CAV1 and CAV2 were expressed low as tumor suppressors in primary BC and cell lines (Bouras et al., 2004). Lee SW reported that the mRNA and protein levels of CAV1 were downregulated or even absent in the BC cell lines, including MCF7, ZR75, T47D, BT20, and MDA-MB231, compared to that of the normal mammary epithelial cells (MCF10A) (Williams and Lisanti, 2004a). CAV1 overexpression could suppress colony formation, matrix invasion, migration, and metastasis (Nwosu et al., 2016). However, increasing evidence suggested that CAV1 was upregulated in BC and positively correlated with aggressive clinical behaviors (Li et al., 2001; Tahir et al., 2001; Savage et al., 2007). Gert G reported that CAV1 and CAV2 were highly expressed in inflammatory breast cancer (IBC), and the CAV1 and CAV2 promoter sites were hypomethylated in SUM149 cells (Van den Eynden et al., 2006). Exogenous expression of CAV1 could significantly promote colony formation in soft agar and inhibit apoptosis in the Hs578T cells (Wu et al., 2007). CAV1 enhanced anchorage-independent survival by upregulating the expression of IGFR and phosphorylated AKT and suppressed the cyclin-dependent kinase inhibitor p21WAF1/Cip1 (Ravid et al., 2005; Van den Eynden et al., 2006; Patani et al., 2012). Therefore, the clinical relevance of CAV1 in BC remains debated with either the tumor suppressor or tumor oncogene. Moreover, there are only a few studies on the function of CAV2 and CAV3 in BC. Therefore, compositive and comprehensive analyses of the function of CAVs in BC are highly warranted.

In the present study, the results indicated that CAV1–3 were all significantly downregulated in most tumor types, especially in BC, lung cancer, ovarian cancer, prostate cancer, and sarcoma cancer. In addition, low expressions of CAV1, CAV2, and CAV3 were closely related to poor OS, RFS, and PPS. According to the HPA database, CAV1, CAV2, and CAV3 were expressed low in BC tissues than in the normal tissues, which were validated by WB analysis. The expressions of CAV1 and CAV2 were negatively correlated with the expression of Vimentin. These results indicated that low CAV expression predicts an unfavorable prognosis in BC patients. Furthermore, we found that patients with CAV mutations exhibited significantly poorer prognoses compared with those without mutations, although the alteration frequencies of CAVs were not high. The previous genomic study has revealed that CAV1 gene mutation occurred in up to ∼16% of breast invasive carcinoma patients (Lee et al., 2002). Christy Moore reported that consistent with the effect of knockout of Cav1, CAV1 gene mutation was molecularly similar to drive metabolic deficiencies, pulmonary hypertension, and reduced spontaneous exercise in mice (Rathinasabapathy et al., 2020). Another report suggested that Cav-1 (P132L) mutation could significantly promote anchorage-independent growth and form tumors in immunodeficient mice (Cerezo et al., 2009). Hyangkyu Lee found that CAV1 mutation was sufficient to result in hyperplasia of mammary epithelial cells, which suggested that Cav-1-null mice may be a well-described animal model to study premalignant mammary disease (Williams and Lisanti, 2005).

Moreover, we found that the methylation levels in the CAV1 and CAV2 promoters were markedly higher, which were significantly associated with TP53 status and history types. We also found that four CpG sites for CAV1, four CpG sites for CAV2, and two CpG sites for CAV3 were associated with poor prognosis. Cui revealed that hypermethylation of the CAV1 promoter was negatively correlated with the inactivation of CAV1 expression (Cui et al., 2001). Similarly, Yan Y Sanders found that CAV1 absence in lung fibroblasts may be regulated by epigenetic mechanisms including histone modifications, in particular H3 lysine 4 trimethylation (Sanders et al., 2017). Leonidas Alevizos reported that hypermethylation-mediated inactivation of CAV1 was associated with nodal metastasis and disease progression in BC (Alevizos et al., 2014). X Rao suggested that lower CAV1 expression and C CGI shore hypermethylation may represent novel prognostic factors for ERα-negative, basal-like BC (Rao et al., 2013). Therefore, hypermethylation may be responsible for the downexpression of CAV1 and represent a new prognostic marker in BC.

Additionally, a comprehensive survival analysis of the CAVs was performed and a prognostic predicting model was conducted by establishing a nomogram and stratified joint-effects survival analysis. The results confirmed that all patients in the study could be divided into low-risk groups and high-risk groups, and patients in high-risk groups had a lower survival probability than the patients in low-risk groups. The ROC curve was created, and the AUCs for 1-, 3-, and 5-year survival were 0.674, 0.567, and 0.536, respectively. Multivariate Cox regression analysis demonstrated that this prognosis model was an independent prognosis predictor for BC patients. Moreover, a nomogram was conducted to predict the 3- and 5-year OS. Therefore, the prognostic model has good predictive power and specificity. Furthermore, this prognostic model could predict different immune statuses and tumor mutation loads, which suggested that patients in the low-risk group tend to have a relatively higher immune status and a lower tumor mutation load compared to the high-risk group.

Recently, the cancer-immunity axis has become the intellectual framework for cancer research and immunotherapy has become one of the most promising approaches for cancer treatment. Studies have demonstrated that tumor mutation load and immune infiltration in the tumor microenvironment can be used as an indicator to predict the immune response after immunotherapy (Goodman et al., 2017; Budczies et al., 2018; Chan et al., 2019b). The present study suggested that patients in the high-risk group might benefit more from the immunotherapeutic treatment than those in the low-risk group, which was consistent with other studies. Aaron M. Goodman reported that higher TMB predicted favorable outcomes for PD-1/PD-L1 blockade across many cancer types (Goodman et al., 2017). Chan suggested that high disease-specific TMB could select the patients benefiting from ICB therapy in lung, bladder, and head and neck cancers (Chan et al., 2019a). Immune checkpoint targets have revolutionized cancer therapy and become the focus of investigation for the treatment of multiple cancer types (Lipson et al., 2015). The CTLA-4 antagonists’ tremelimumab and ipilimumab have been used in small breast cancer trials, with evidence of downregulating Tregs tumor infiltration in breast cancer (Emens et al., 2017). In addition, extensive clinical data show that there are currently many immunotherapies used to treat patients with metastatic TNBC that target the PD-1/PD-L1 signaling, including avelumab, pembrolizumab, atezolizumab, and pembrolizumab (Emens, 2012). The utility of PD-1/PD-L1 blockade for TNBC in the adjuvant and neoadjuvant settings is under intensive investigation. I-SPY trial suggested that neoadjuvant administration of pembrolizumab plus paclitaxel may result in an estimated pCR rate of 46% in HER-2 patients and 60% in TNBC patients (Nanda et al., 2020). A successful Phase 3 trial revealed that the addition of pembrolizumab to paclitaxel results in a superior CPR rate estimated at >99% for all HER-2 patient subgroups (Schmid et al., 2020). Therefore, immune checkpoint blockade has gradually become a new method for tumor therapy (Emens, 2018). Moreover, our module could predict the responsiveness of chemotherapy drugs, including gefitinib, gemcitabine, lapatinib, paclitaxel, vorinostat, bicalutamide, cisplatin, and docetaxel. Therefore, our prediction module may provide clinical guidance for drug combination therapy and medication instruction in BC.

To investigate the underlying mechanism of CAVs in BC, we performed GO annotation and KEGG pathway analysis and found that these genes were particularly enriched in cell adhesion, migration, cell junction organization, endocytosis, the ErbB signaling pathway, and the MAPK signaling pathway. Previous studies have reported that some mechanisms of CAVs in several cancers were mainly attributed to the regulation of EMT-related signaling pathways. Hongxiu Yu reported that CAV1 could promote the EMT process via the Wnt/β-catenin pathway in hepatocellular carcinoma (Yu et al., 2014). Kundong Zhang reported that knockdown of CAV1 suppressed the expression of E-cadherin and enhanced cell migration ability in gastric cancer (Zhang et al., 2016). CAV1 deletion cells displayed enhanced EMT and premetastatic properties in head and neck carcinoma (Jung et al., 2015). To further validate the function of CAVs, we performed *in vivo* experiments, and the results suggested that CAV knockdown could promote cell growth, migration, invasion, and stemness maintenance in BC cells, which is consistent with findings from previous research (Shan-Wei et al., 2012; Wang et al., 2020; Ren et al., 2021; Wu et al., 2021). Furthermore, we found that CAV1 and CAV2 might be involved in EGF-mediated endocytosis trafficking. Overall, CAVs and the related pathways may act as biomarkers or therapeutic targets for clinical treatment.

Nevertheless, there were several limitations in the present study. In the first place, we investigated the prognosis value of CAVs mainly depending on the retrospective public data set of different cohorts, which might cause selection bias and be somewhat heterogeneous in data processing and patient population. Second, the prognostic signature of CAVs was identified from TCGA, and the sample size is relatively small, which may cause some bias. Therefore, the expression profiles and the prognosis value of CAVs require further validation by means of clinical research. Third, for the data of the immunotherapy cohorts of BRCA that are not available, we used urothelial carcinoma to investigate the relationship between immunotherapy response and our risk signature. Similar to our study, previous studies also used this UC cohort to investigate whether their risk signature could predict patients’ response to immune checkpoint blockade therapy in different cancer types. Therefore, future studies should take these factors into account to validate the current findings.

## Conclusion

The present study revealed the specific patterns of CAV expression and assessed the prognostic value in BC via integrated bioinformatics analysis. We found that low expression of CAVs was notably correlated with an aggressive phenotype and poorer prognosis for BC patients. Then we constructed a prognostic model based on the expression profiles of CAVs, which divided BC patients into two risk groups. Patients in the high-risk group tended to have a poorer prognosis and a higher mutation event frequency compared to the low-risk group, suggesting that risk score was an independent risk factor for BC patients. Furthermore, this signature could effectively predict the response to chemotherapy and immunotherapy. Finally, loss of function studies strongly confirmed that CAVs exert the tumor suppressor role in BC cells.

## Data Availability

The data sets presented in this study can be found in online repositories. The names of the repository/repositories and accession number(s) can be found in the article/Supplementary Material.
