# Supplementary material for: Identification and validation of a prognostic risk model based on caveolin family genes for breast cancer
Source: Front Cell Dev Biol. 2022 Sep 6;10:822187. doi: 10.3389/fcell.2022.822187 (PMC9485841; doi:10.3389/fcell.2022.822187)
Supplement: Supplementary file 1 [file DataSheet2.pdf]

Figure 1A

https://www.oncomine.org/resource/login.html

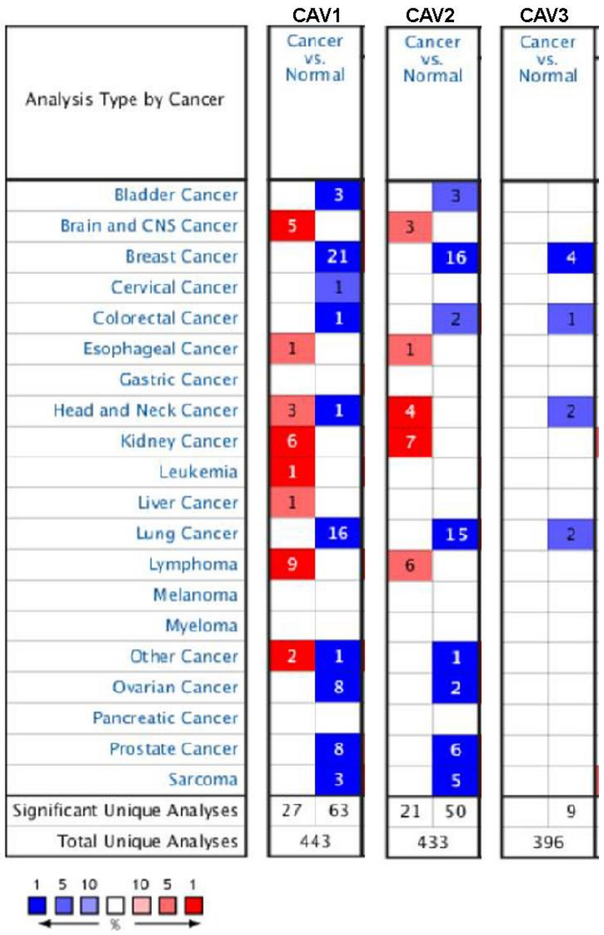

http://timer.cistrome.org

Figure 1 B

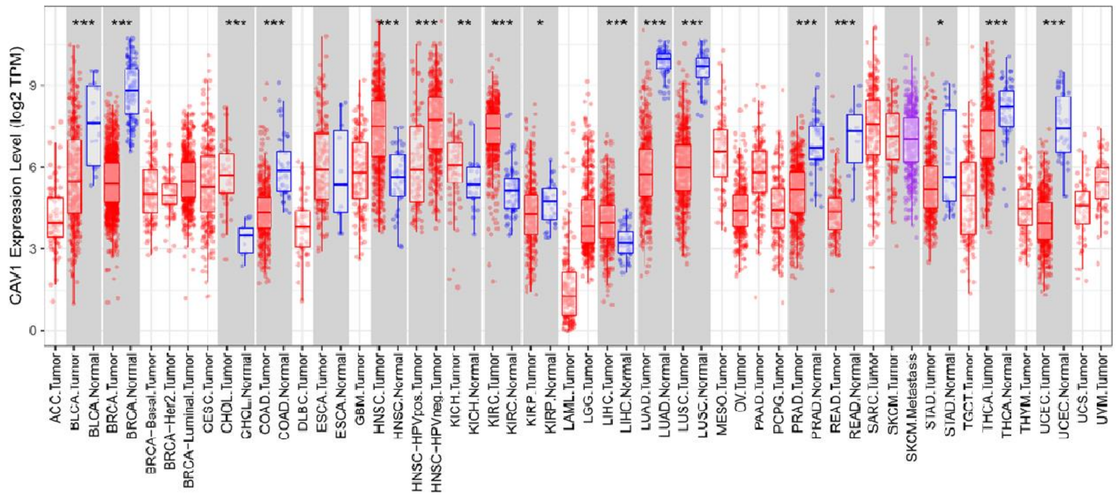

Figure 1 C

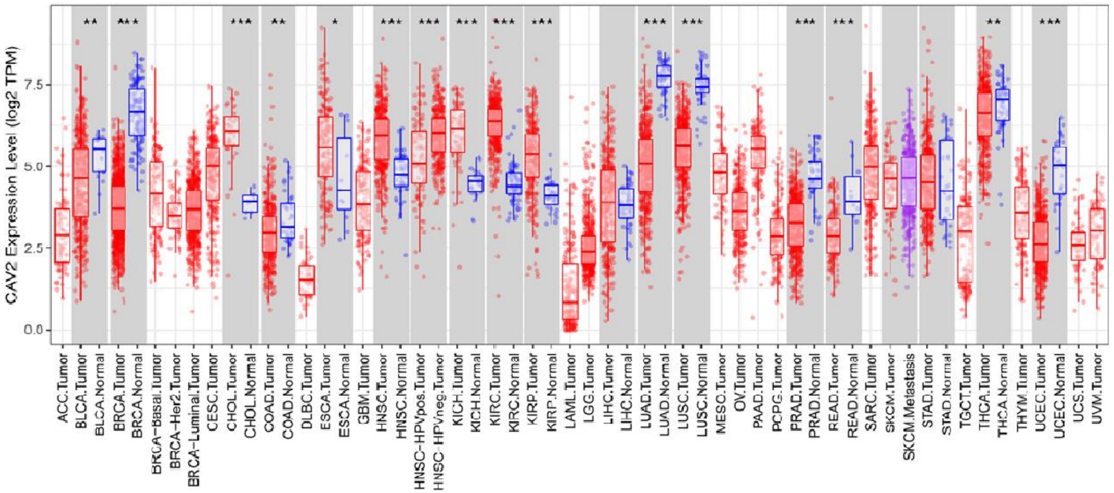

Figure 1 D

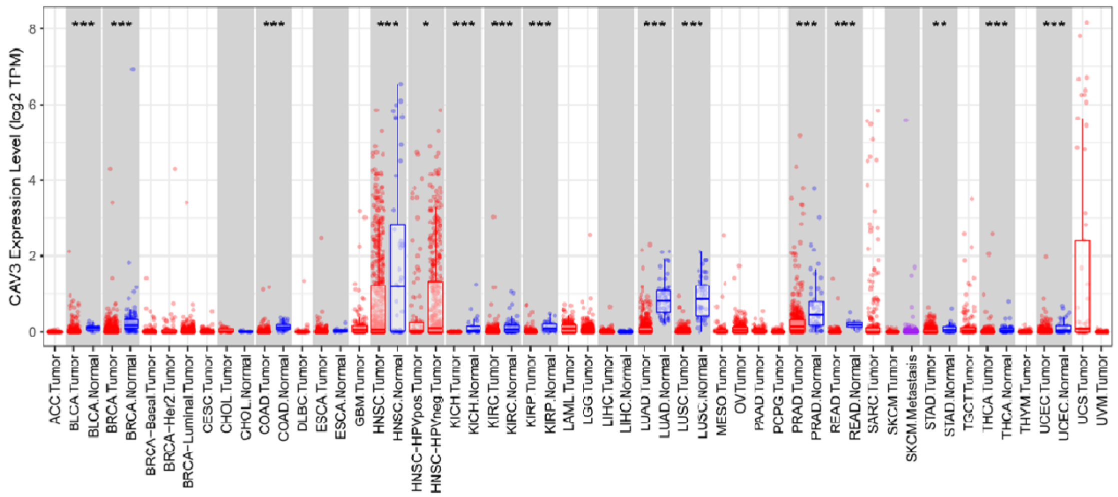

http://ualcan.path.uab.edu/

Figure 1 E

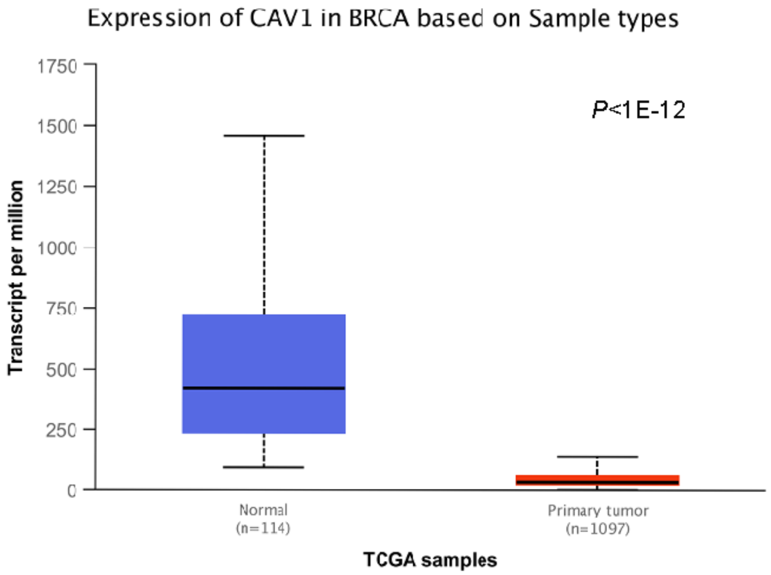

Figure 1 F

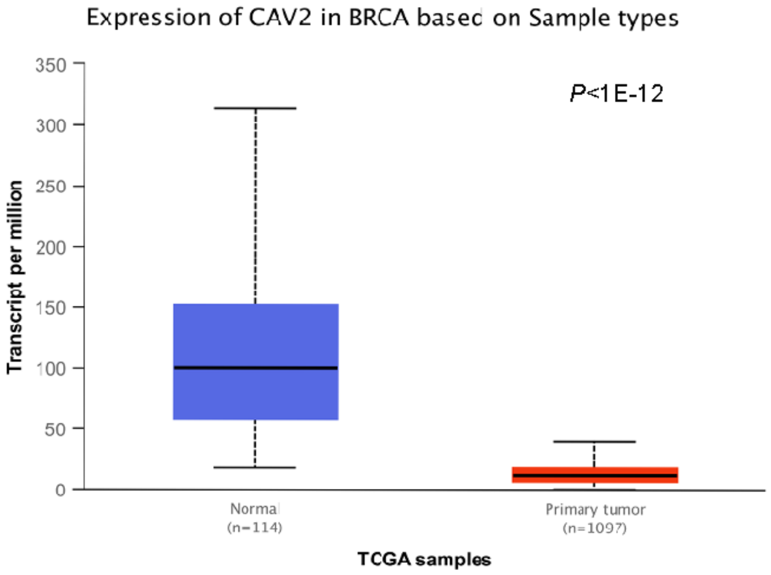

Figure 1 G

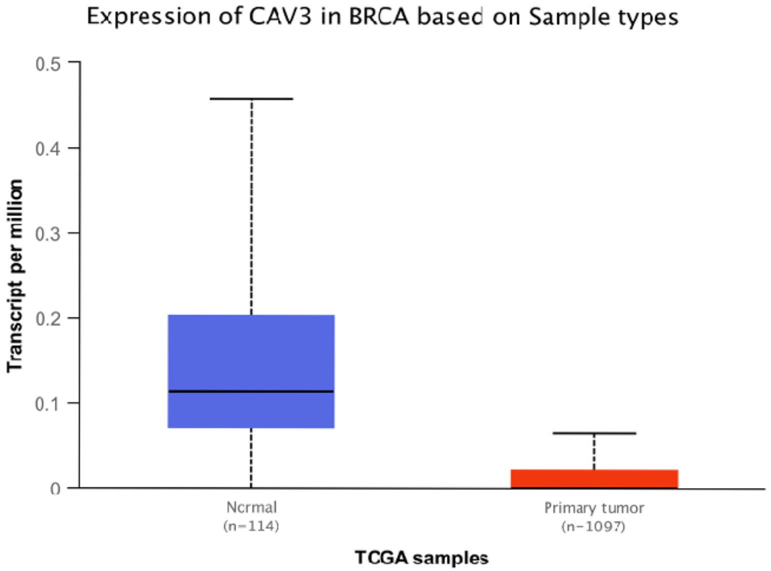

Figure 2

http://bcgenex.ico.unicancer.fr/BC-GEM/GEM-Accueil.php?js=1

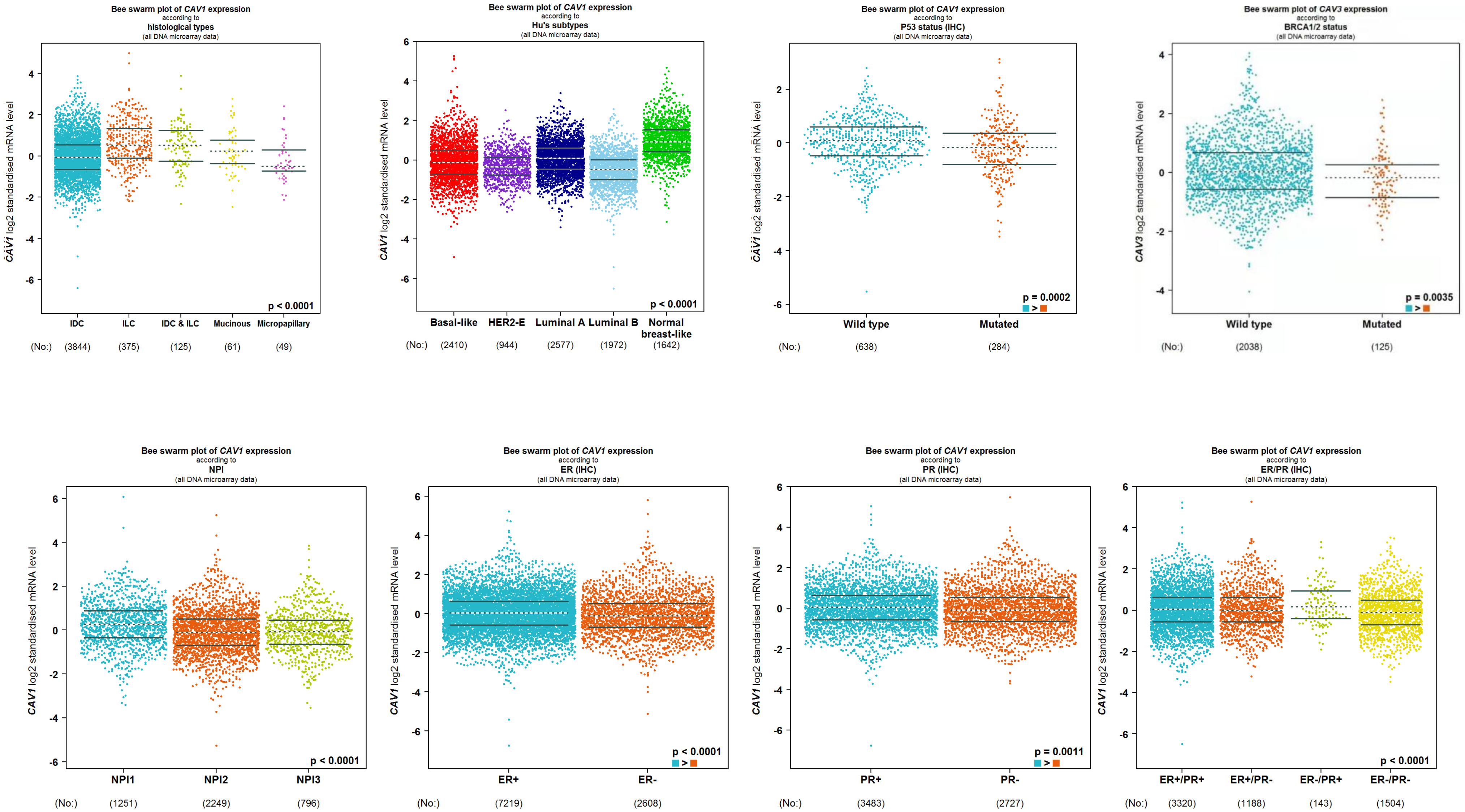

Figure 2

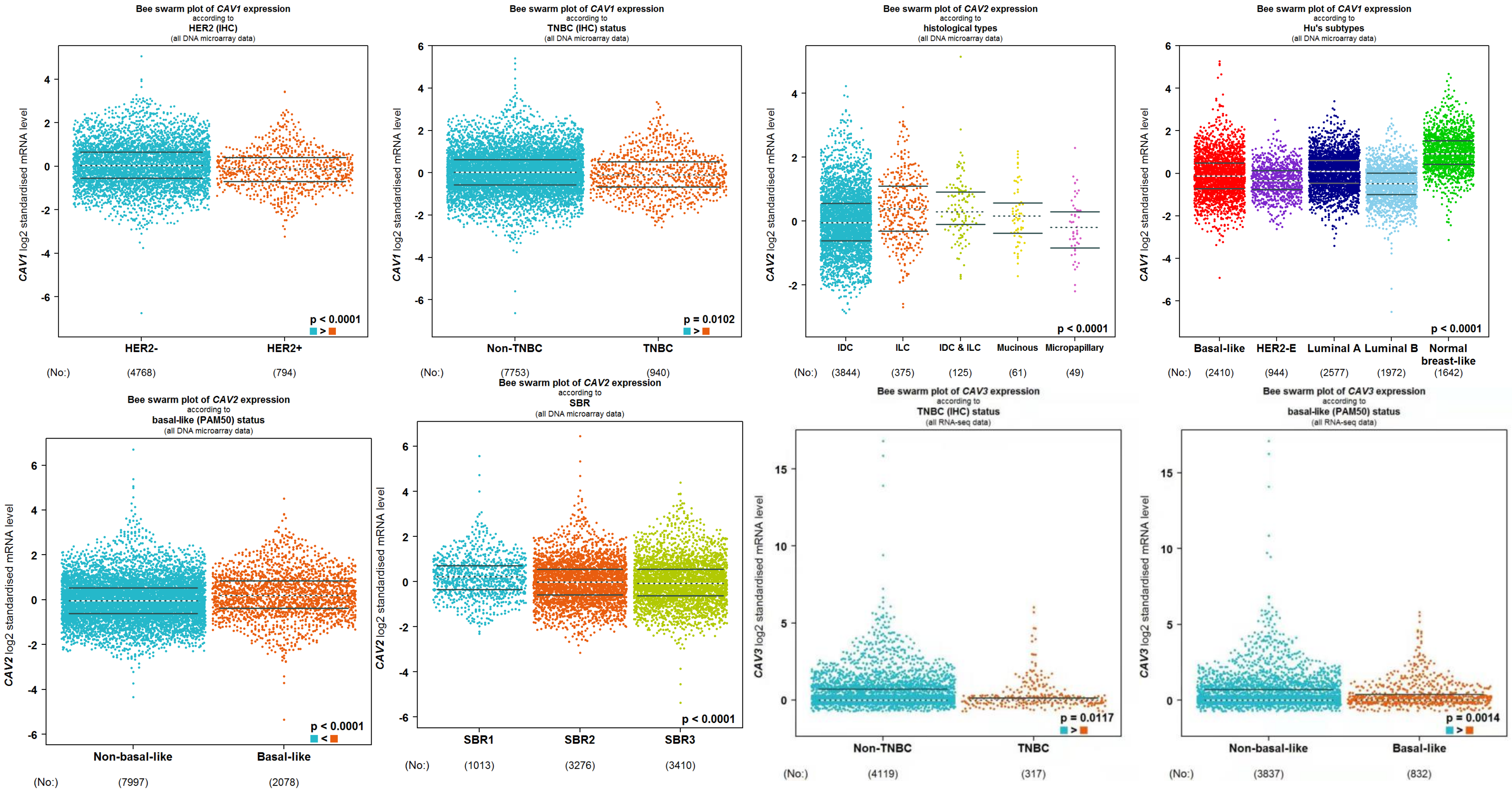
Bee swarm plot of CAV1 expression
according to
HER2 (IHC)
(all DNA microarray data)
CAV1 log2 standardised mRNA level
p < 0.0001
HER2-
HER2+
(No:) (4768) (794)
Bee swarm plot of CAV1 expression
according to
TNBC (IHC) status
(all DNA microarray data)
CAV1 log2 standardised mRNA level
p = 0.0102
Non-TNBC
TNBC
(No:) (7753) (940)
Bee swarm plot of CAV2 expression
according to
histological types
(all DNA microarray data)
CAV2 log2 standardised mRNA level
p < 0.0001
IDC
ILC
IDC & ILC
Mucinous
Micropapillary
(No:) (3844) (375) (125) (61) (49)
Bee swarm plot of CAV1 expression
according to
Hu's subtypes
(all DNA microarray data)
CAV1 log2 standardised mRNA level
p < 0.0001
Basal-like
HER2-E
Luminal A
Luminal B
Normal breast-like
(No:) (2410) (944) (2577) (1972) (1642)
Bee swarm plot of CAV2 expression
according to
basal-like (PAM50) status
(all DNA microarray data)
CAV2 log2 standardised mRNA level
p < 0.0001
Non-basal-like
Basal-like
(No:) (7997) (2078)
Bee swarm plot of CAV2 expression
according to
SBR
(all DNA microarray data)
CAV2 log2 standardised mRNA level
p < 0.0001
SBR1
SBR2
SBR3
(No:) (1013) (3276) (3410)
Bee swarm plot of CAV3 expression
according to
TNBC (IHC) status
(all RNA-seq data)
CAV3 log2 standardised mRNA level
p = 0.0117
Non-TNBC
TNBC
(No:) (4119) (317)
Bee swarm plot of CAV3 expression
according to
basal-like (PAM50) status
(all RNA-seq data)
CAV3 log2 standardised mRNA level
p = 0.0014
Non-basal-like
Basal-like
(No:) (3837) (832)

Figure 2

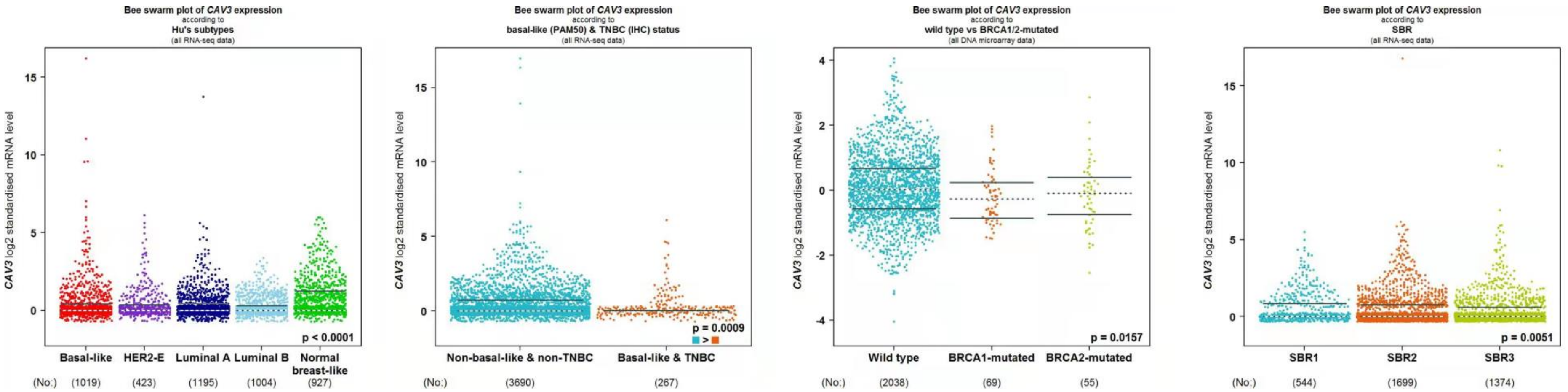
Bee swarm plot of CAV3 expression
according to
Hu's subtypes
(all RNA-seq data)
CAV3 log2 standardised mRNA level
p < 0.0001
Basal-like
HER2-E
Luminal A
Luminal B
Normal breast-like
(No:) (1019) (423) (1195) (1004) (927)
Bee swarm plot of CAV3 expression
according to
basal-like (PAM50) & TNBC (IHC) status
(all RNA-seq data)
CAV3 log2 standardised mRNA level
p = 0.0009
Non-basal-like & non-TNBC
Basal-like & TNBC
(No:) (3690) (267)
Bee swarm plot of CAV3 expression
according to
wild type vs BRCA1/2-mutated
(all DNA microarray data)
CAV3 log2 standardised mRNA level
p = 0.0157
Wild type
BRCA1-mutated
BRCA2-mutated
(No:) (2038) (69) (55)
Bee swarm plot of CAV3 expression
according to
SBR
(all RNA-seq data)
CAV3 log2 standardised mRNA level
p = 0.0051
SBR1
SBR2
SBR3
(No:) (544) (1699) (1374)

http://kmplot.com/analysis/

Figure 3

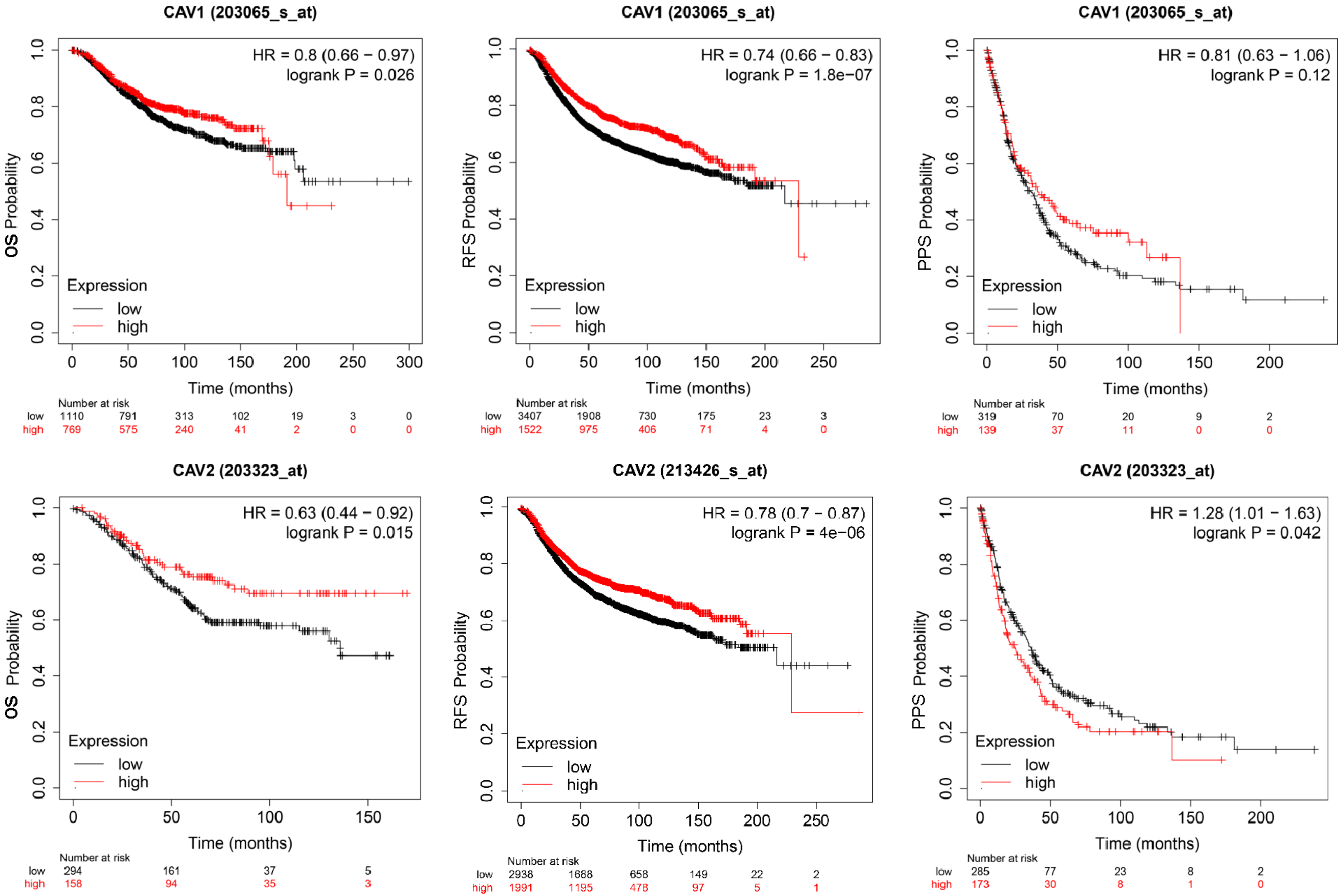

http://kmplot.com/analysis/

Figure 3

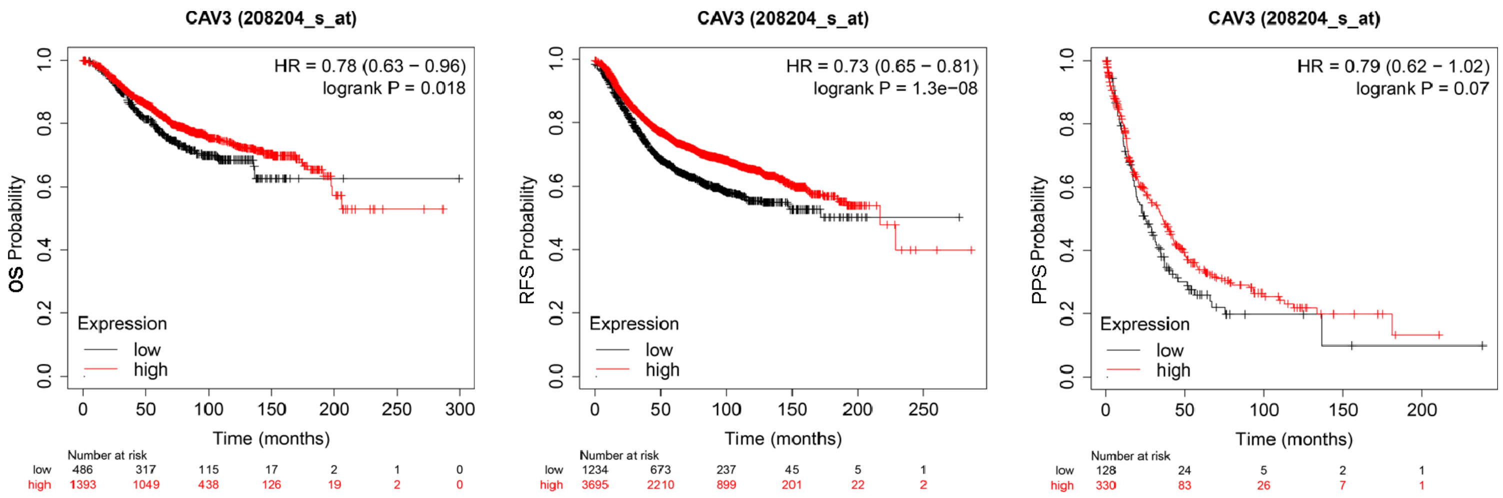

http://ualcan.path.uab.edu/

Figure 4 A

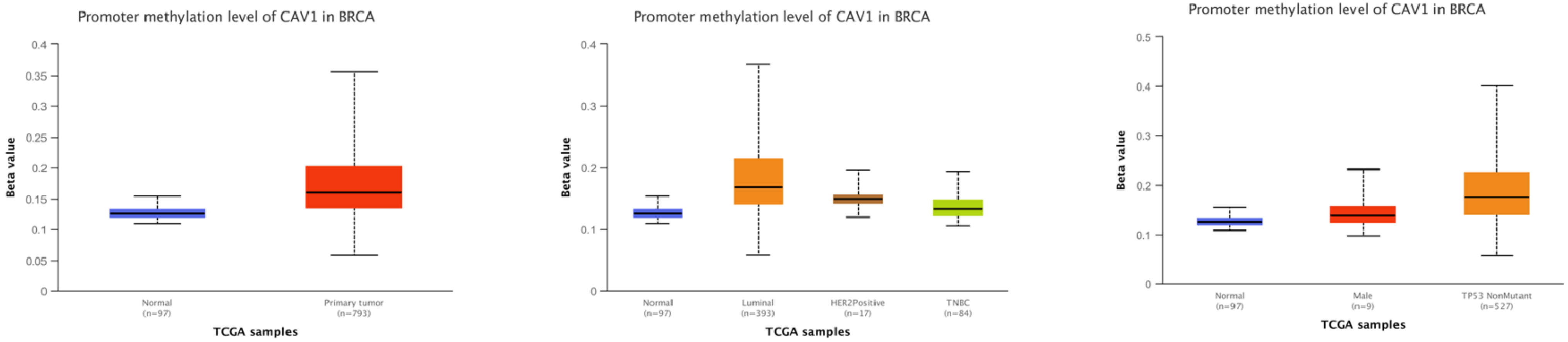

Figure 4 B

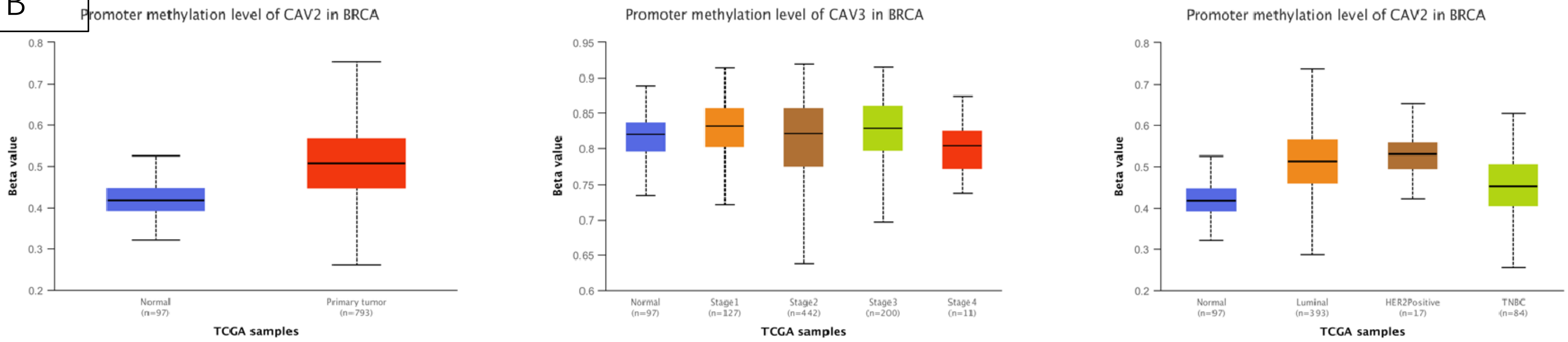

http://ualcan.path.uab.edu/

Figure 4 C

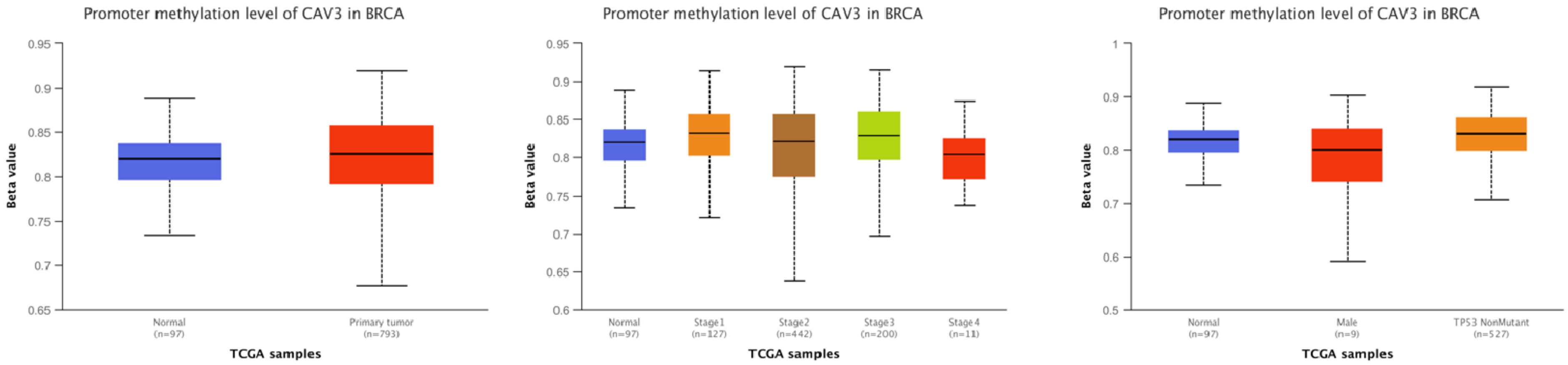

Figure 5 A

https://www.ebi.ac.uk/ebisearch/search.ebi?db=allebi&query=CAV1&requestFrom=searchBox

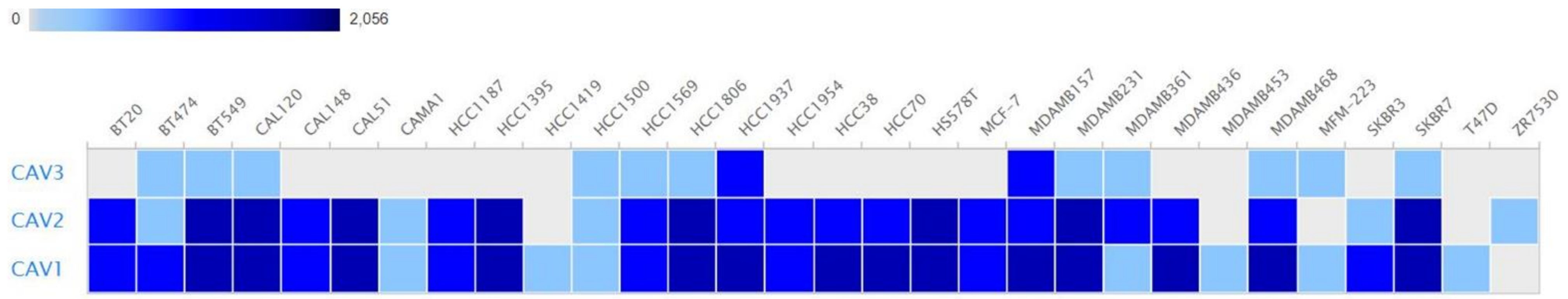

https://www.proteinatlas.org/

Figure 5 B

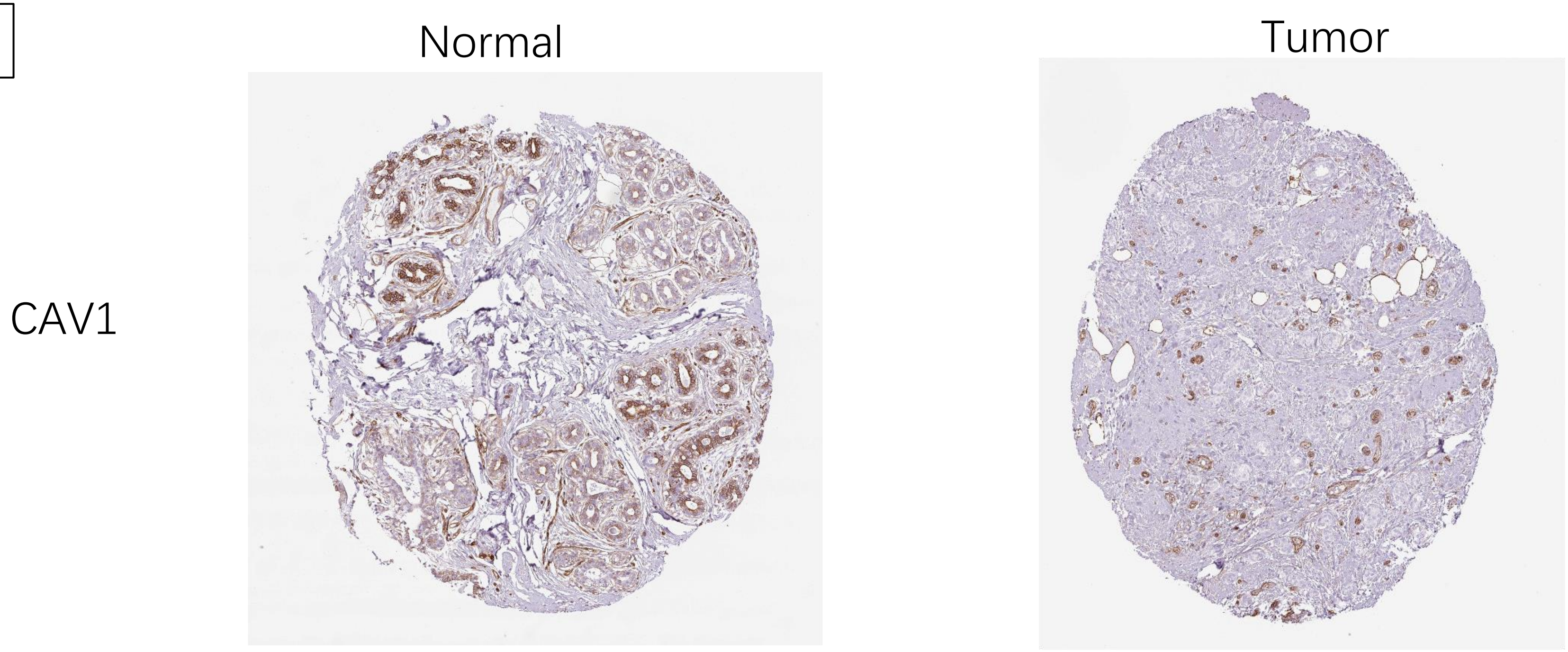

https://www.proteinatlas.org/

Figure 5 B

Normal

Tumor

CAV2

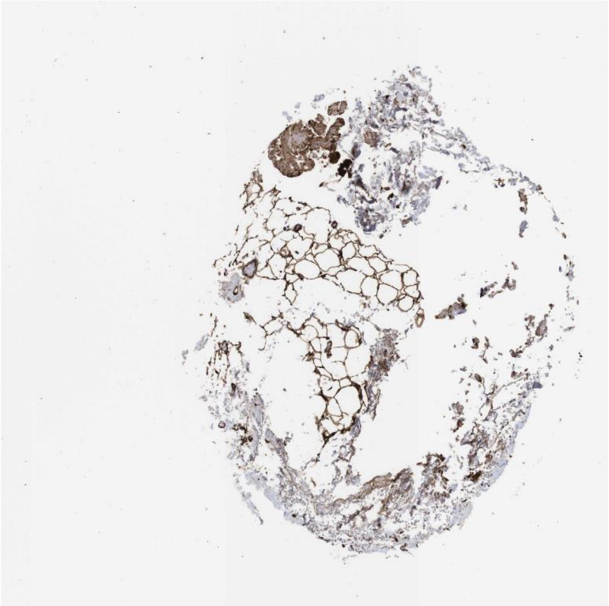

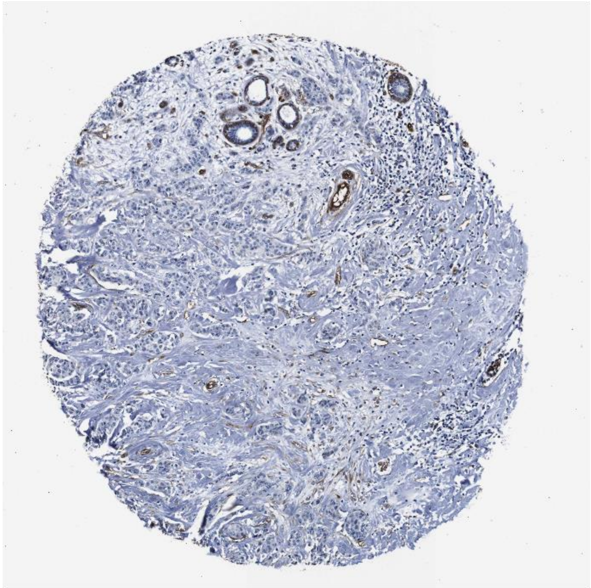

Figure 5 B

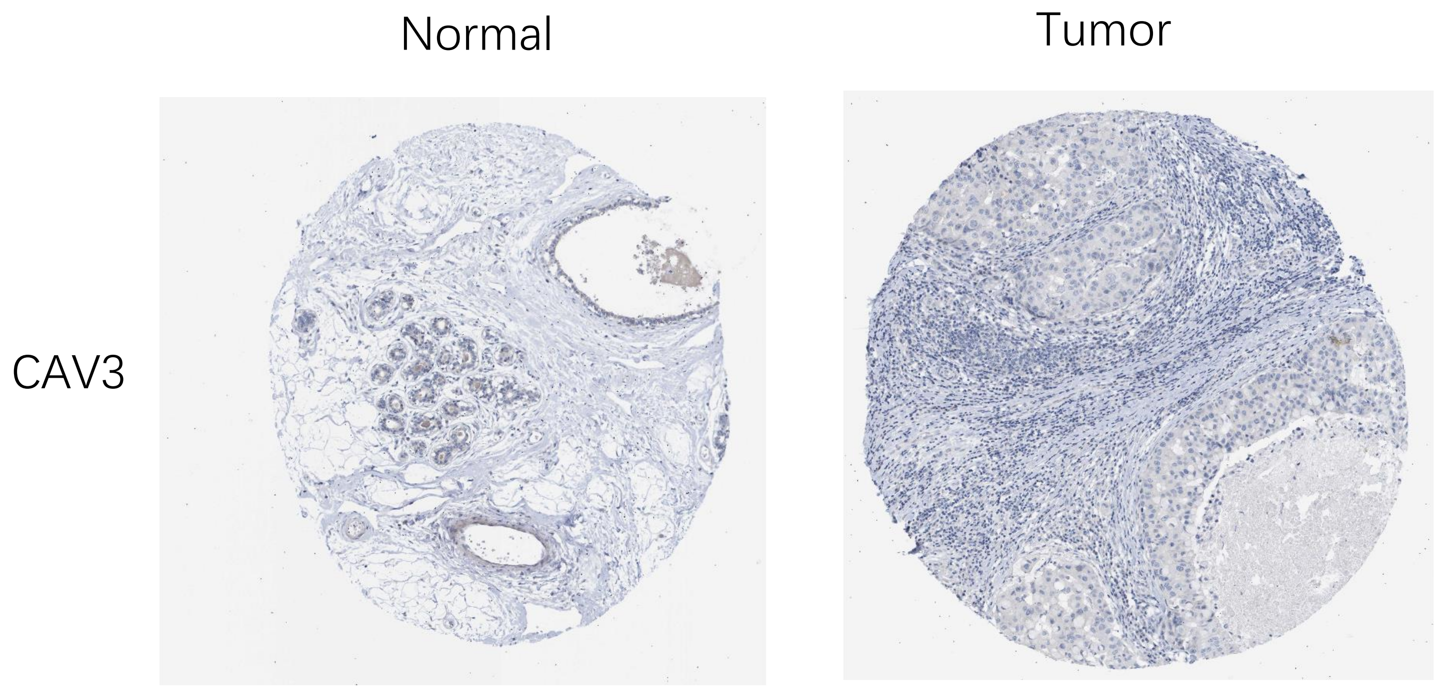

Figure 5 C

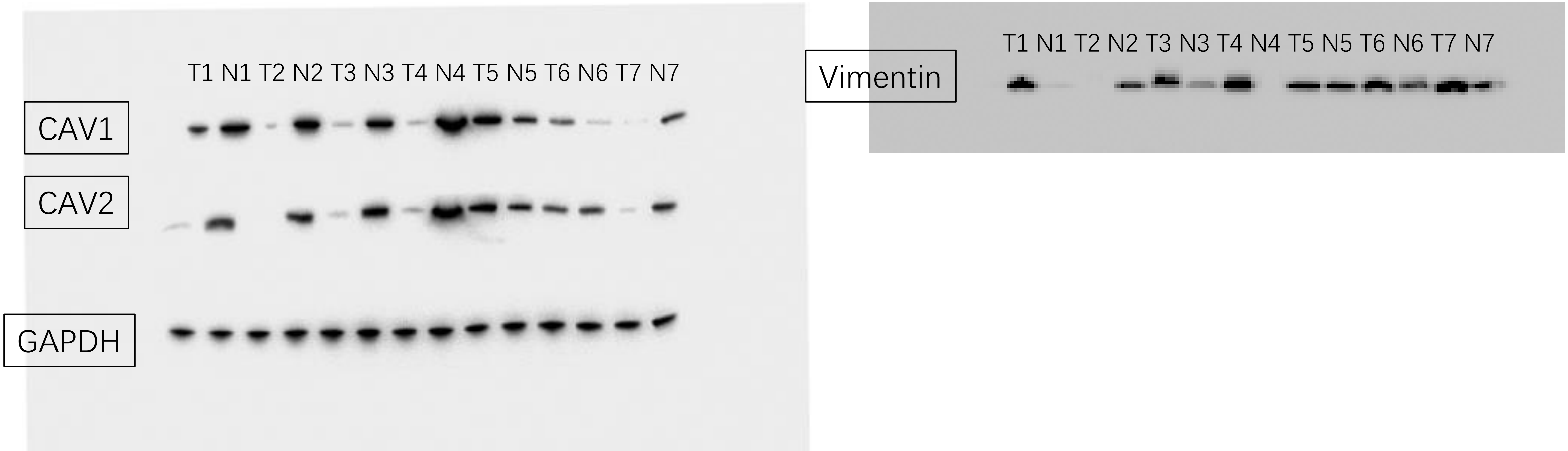
T1 N1 T2 N2 T3 N3 T4 N4 T5 N5 T6 N6 T7 N7
CAV1
CAV2
GAPDH
Vimentin
T1 N1 T2 N2 T3 N3 T4 N4 T5 N5 T6 N6 T7 N7

Figure 5 D

https://cistrome.shinyapps.io/timer/

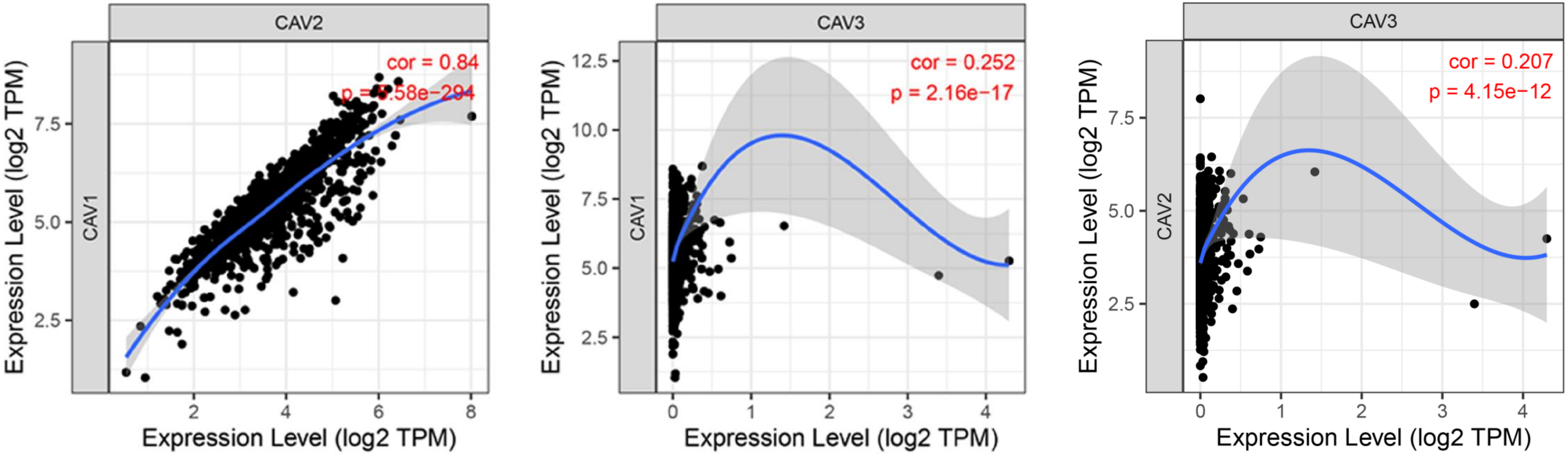

Figure 5 E

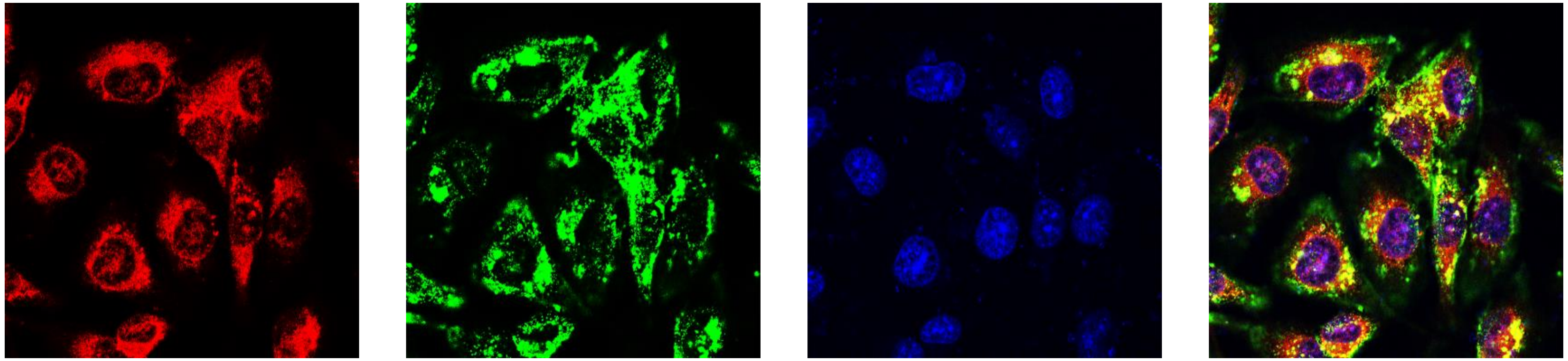

http://genemania.org/

https://metascape.org/gp/index.html#/main/step1

Figure 10 A

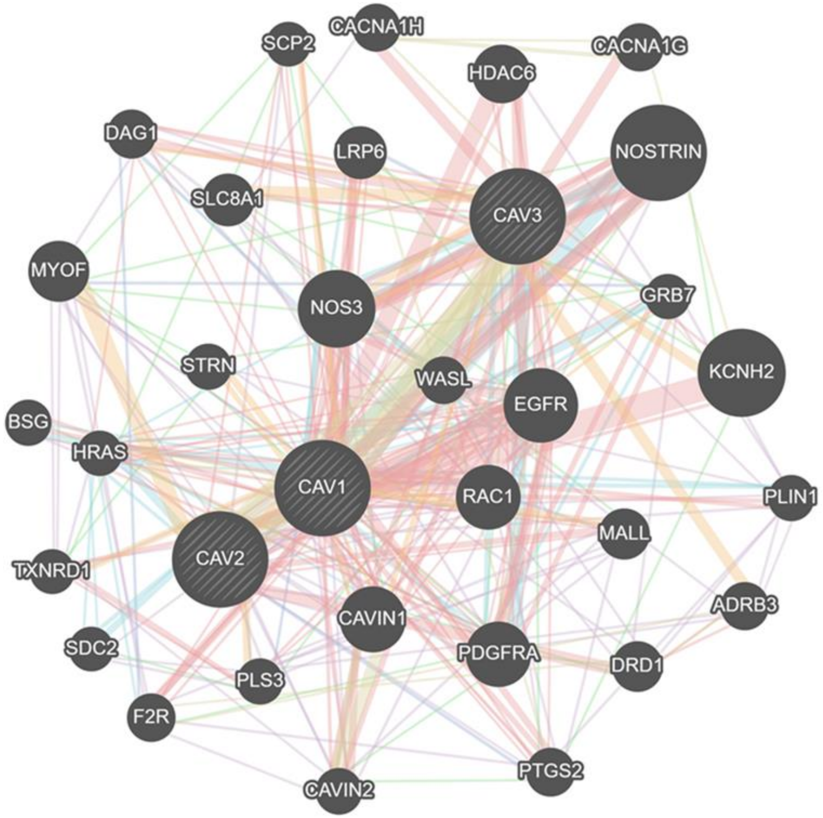

Figure 10 B

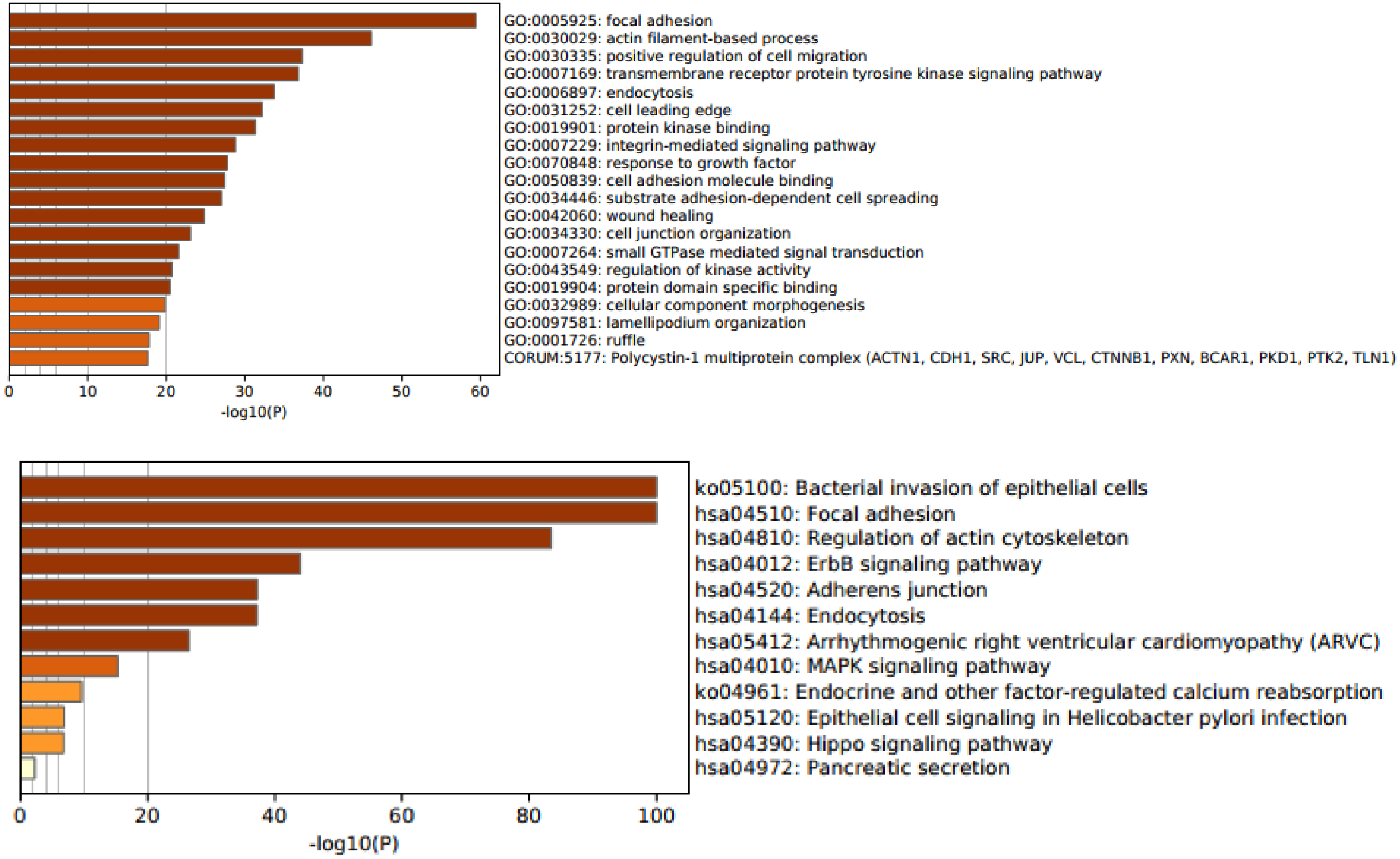

Figure 10 C

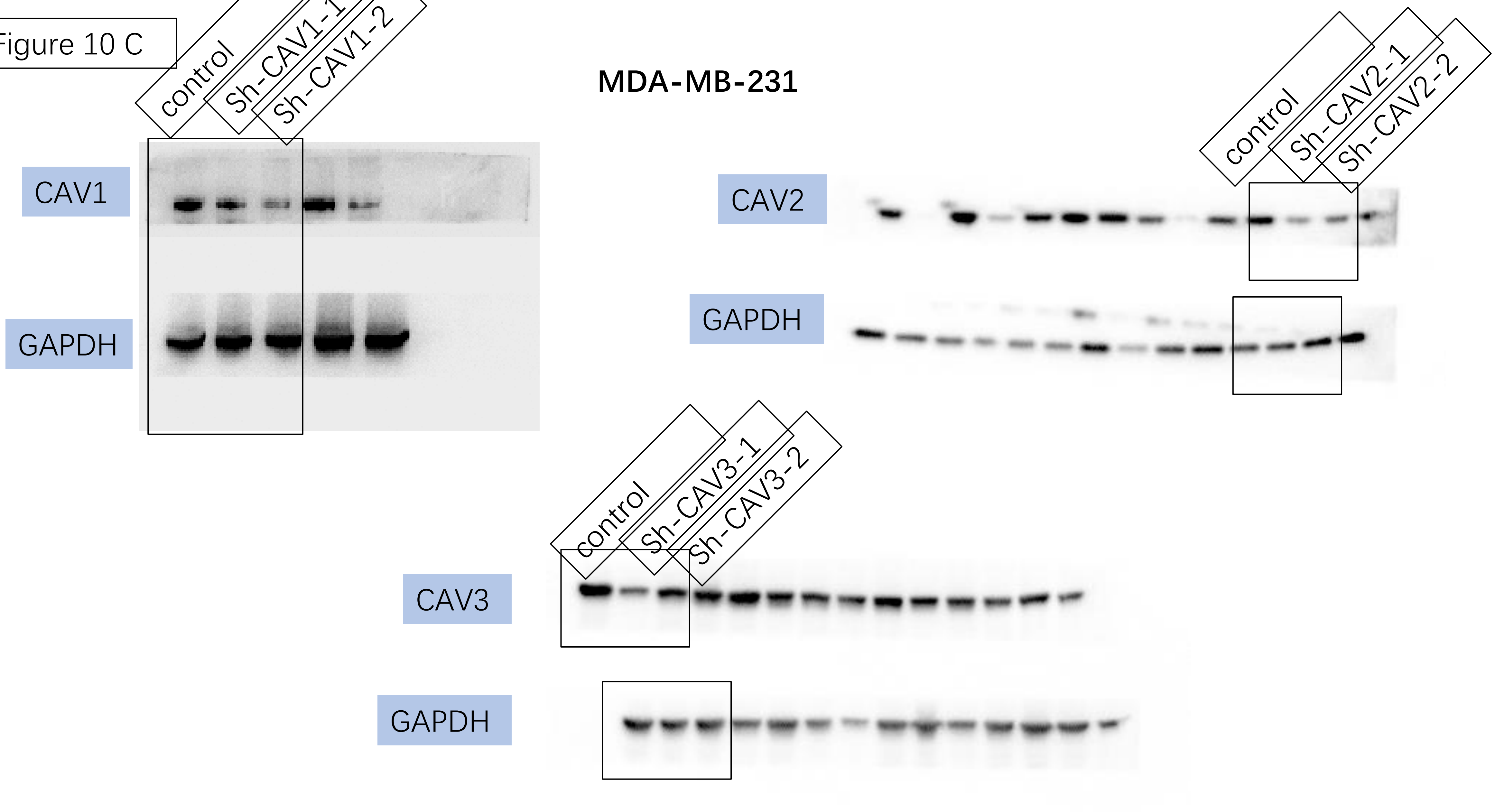
MDA-MB-231
control
Sh-CAV1-1
Sh-CAV1-2
CAV1
GAPDH
control
Sh-CAV2-1
Sh-CAV2-2
CAV2
GAPDH
control
Sh-CAV3-1
Sh-CAV3-2
CAV3
GAPDH

Figure 10 D

MDA-MB-231

control

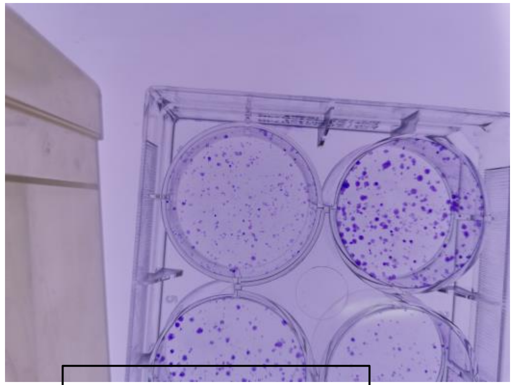

Sh-CAV1-1

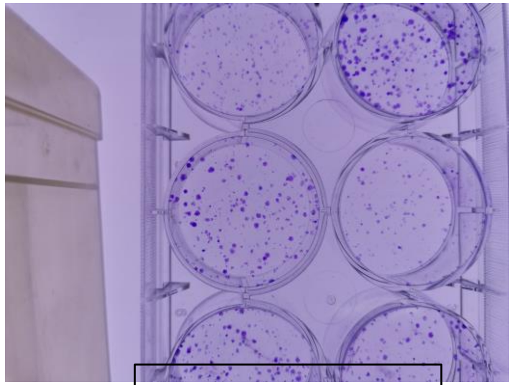

Sh-CAV1-2

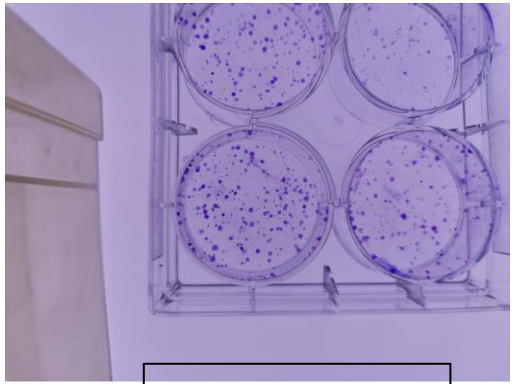

control

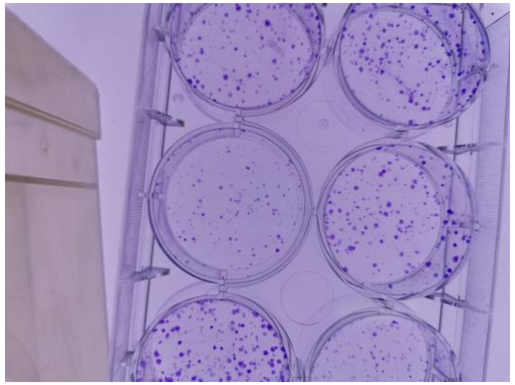

Sh-CAV2-1

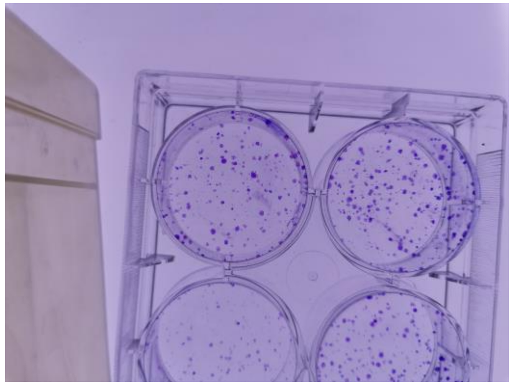

Sh-CAV2-2

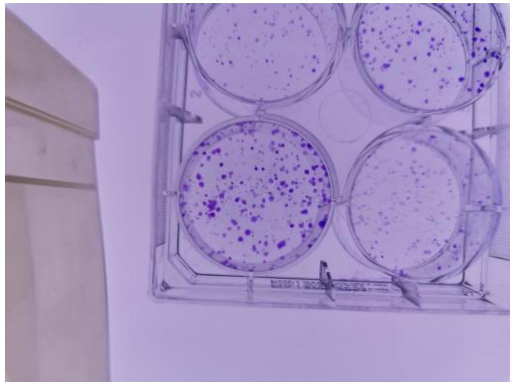

Figure 10 D

MDA-MB-231

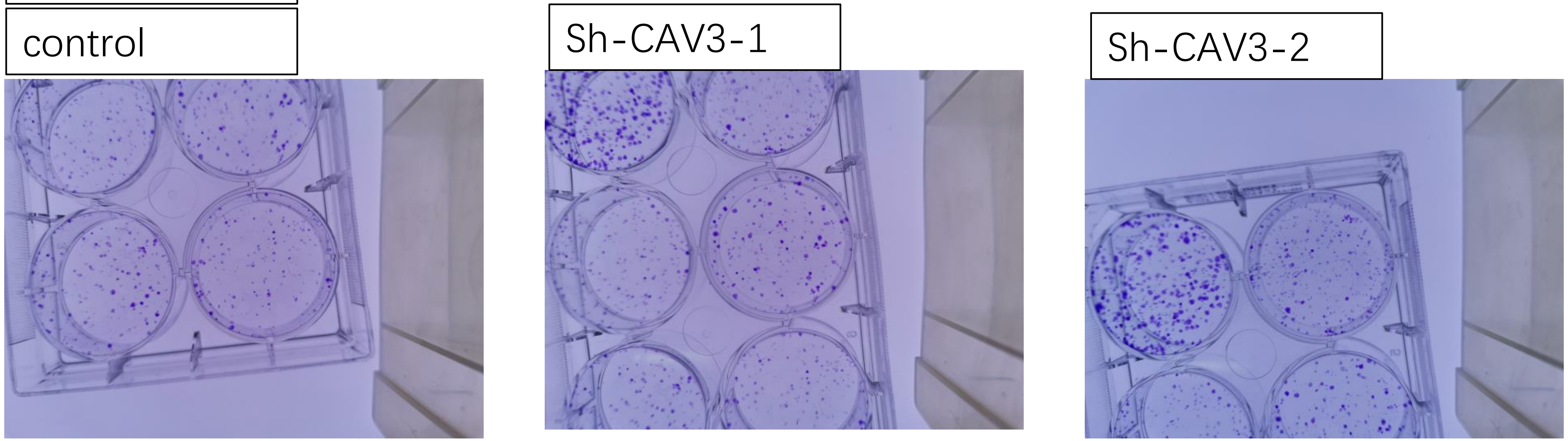

MDA-MB-231

Figure 10 E

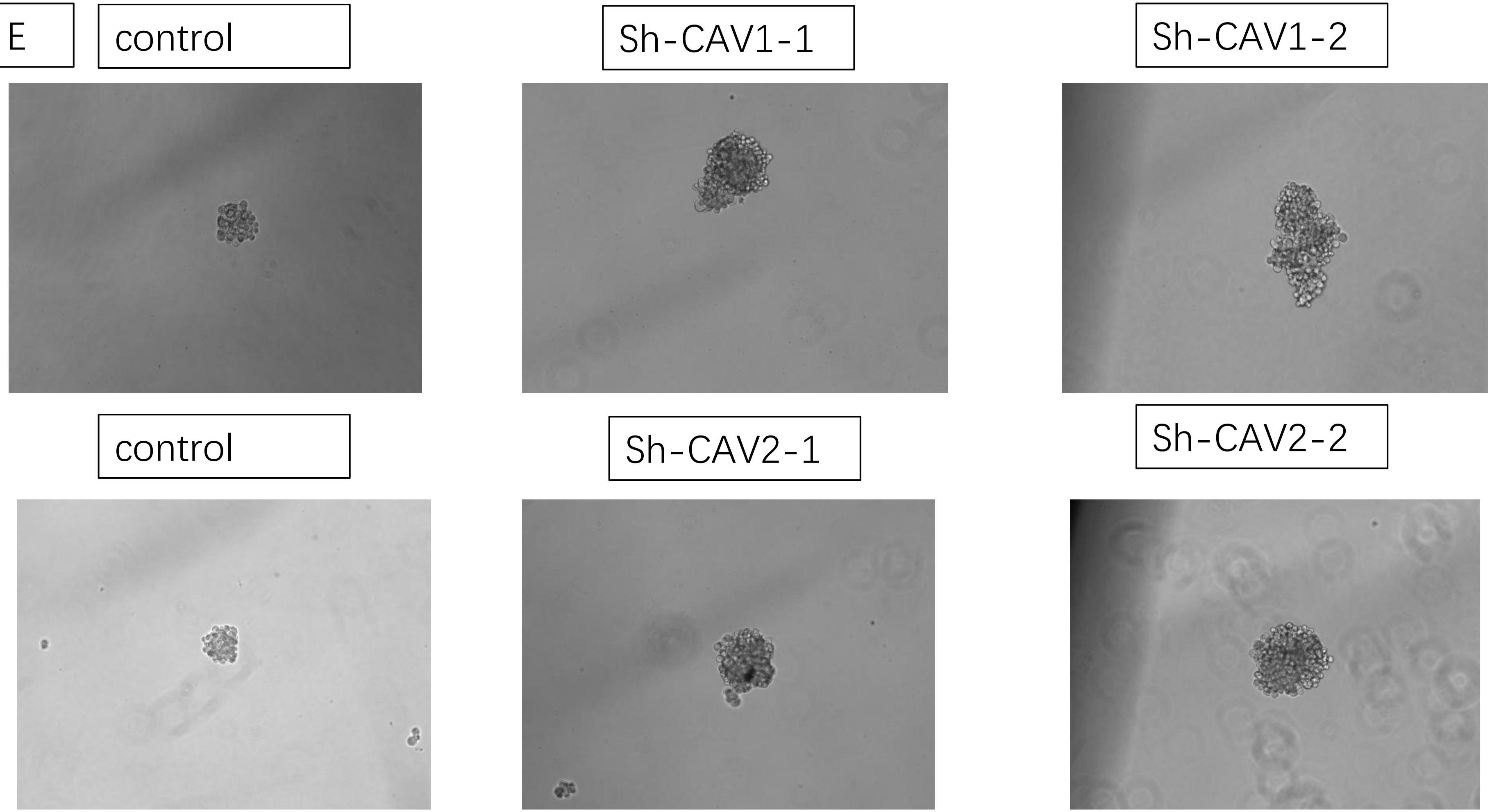

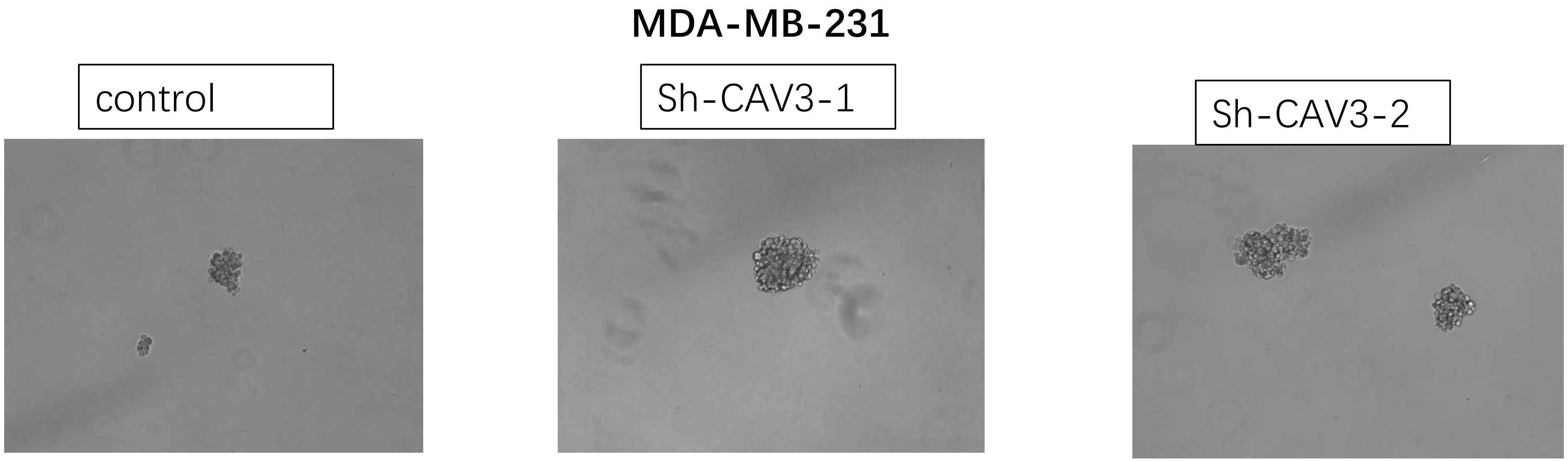
MDA-MB-231
control
Sh-CAV3-1
Sh-CAV3-2

Figure 11 A

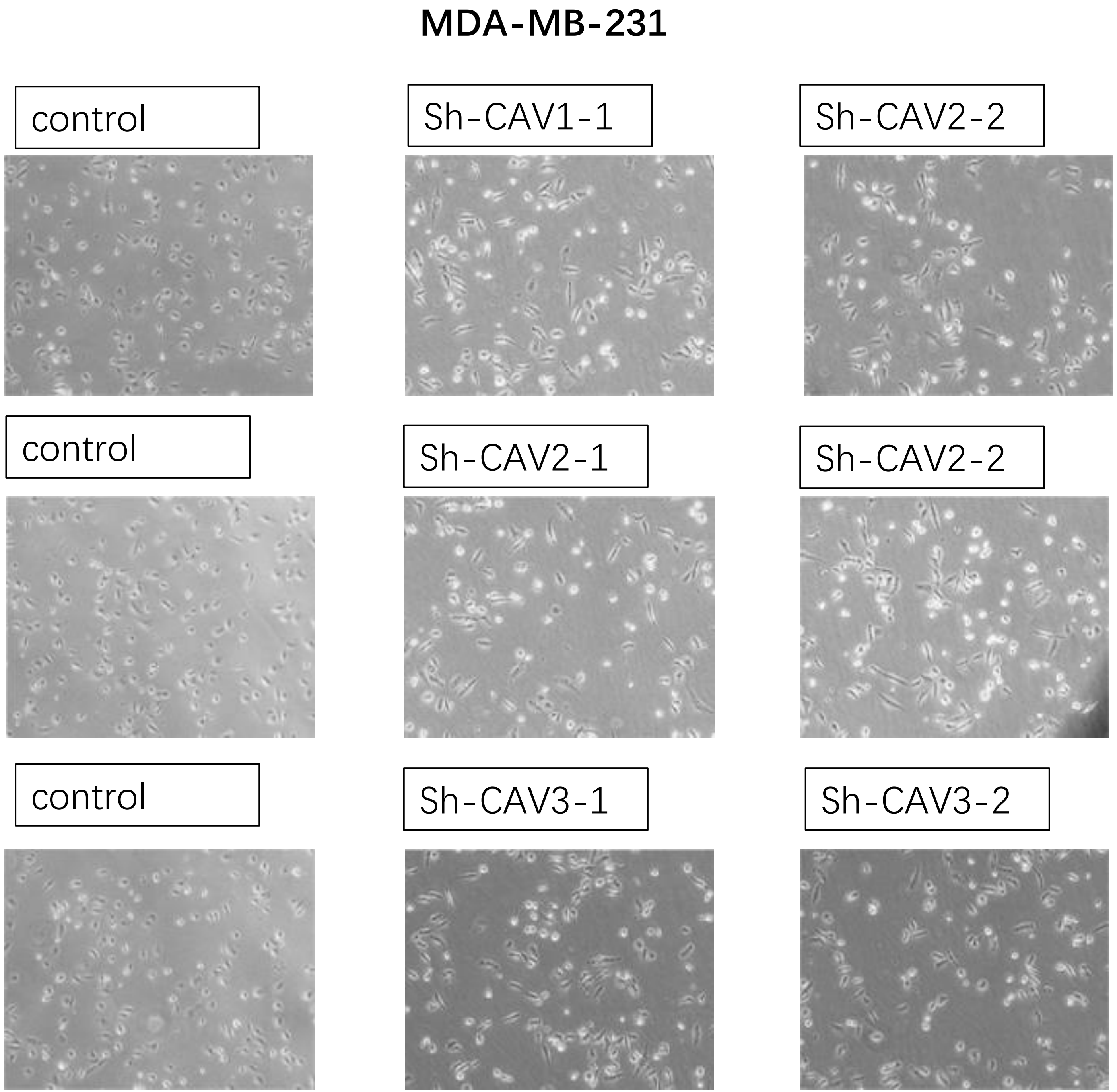
MDA-MB-231
control
Sh-CAV1-1
Sh-CAV2-2
control
Sh-CAV2-1
Sh-CAV2-2
control
Sh-CAV3-1
Sh-CAV3-2

Figure 11 B

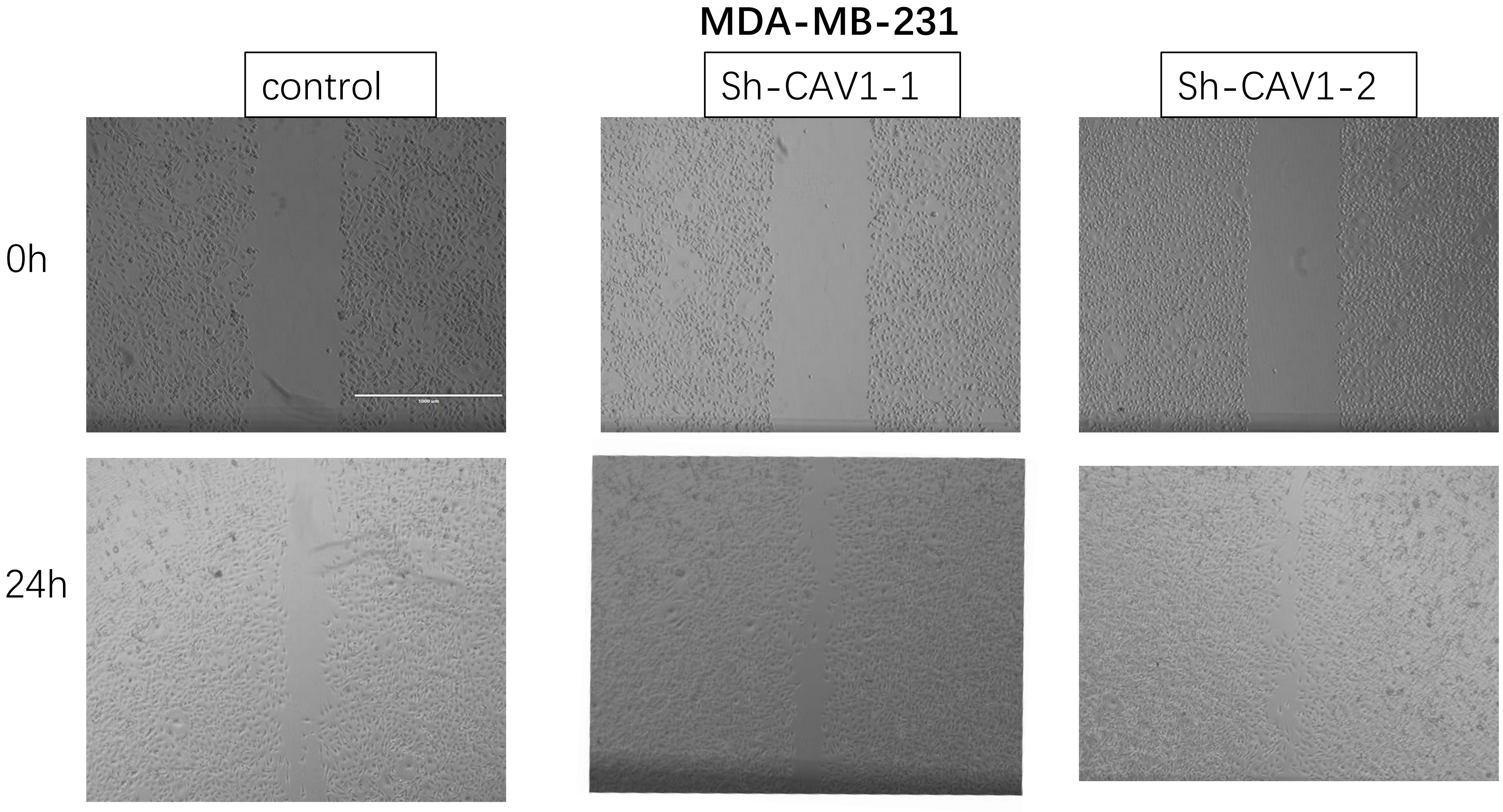

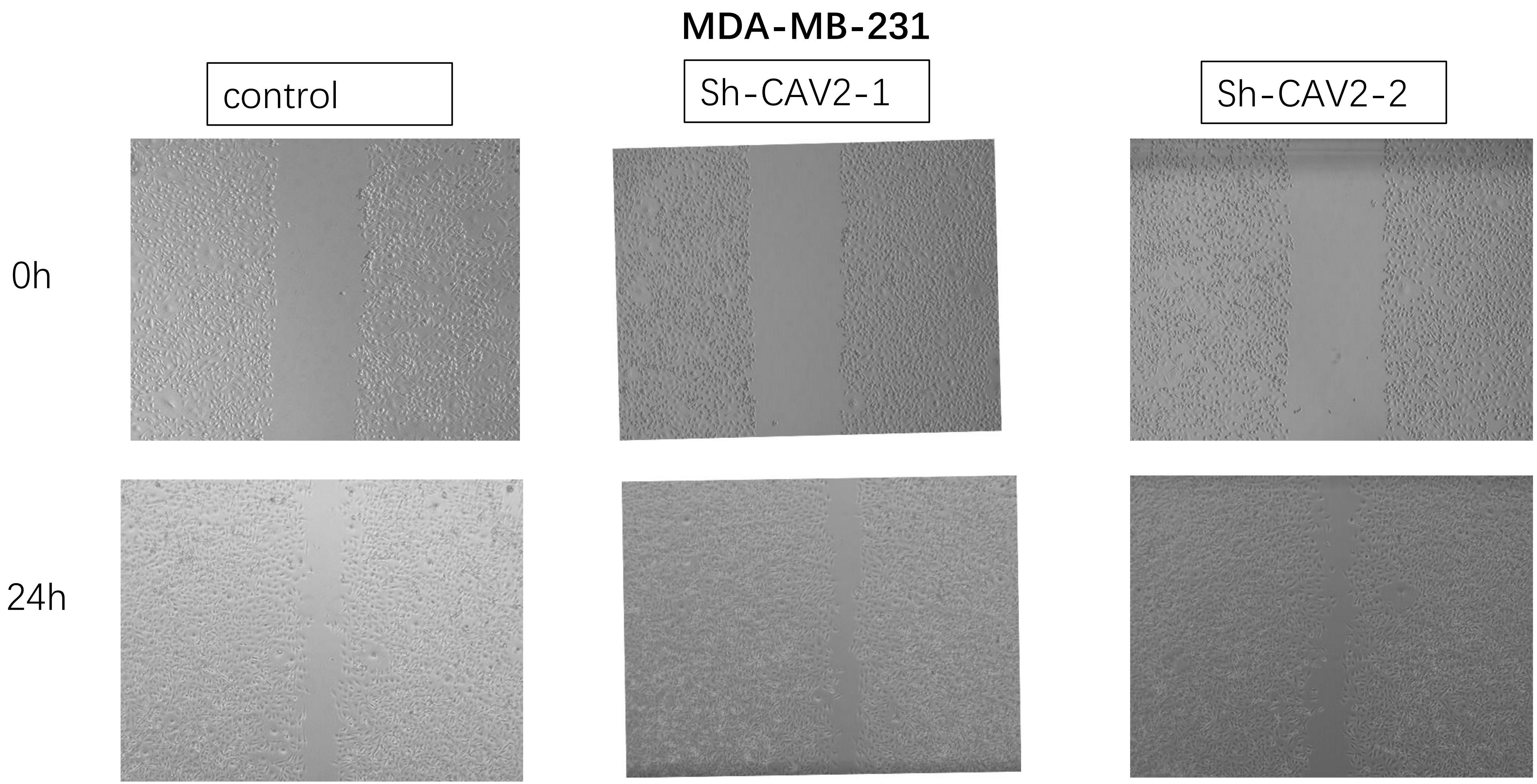
MDA-MB-231
control
Sh-CAV2-1
Sh-CAV2-2
0h
24h

Figure 11 B

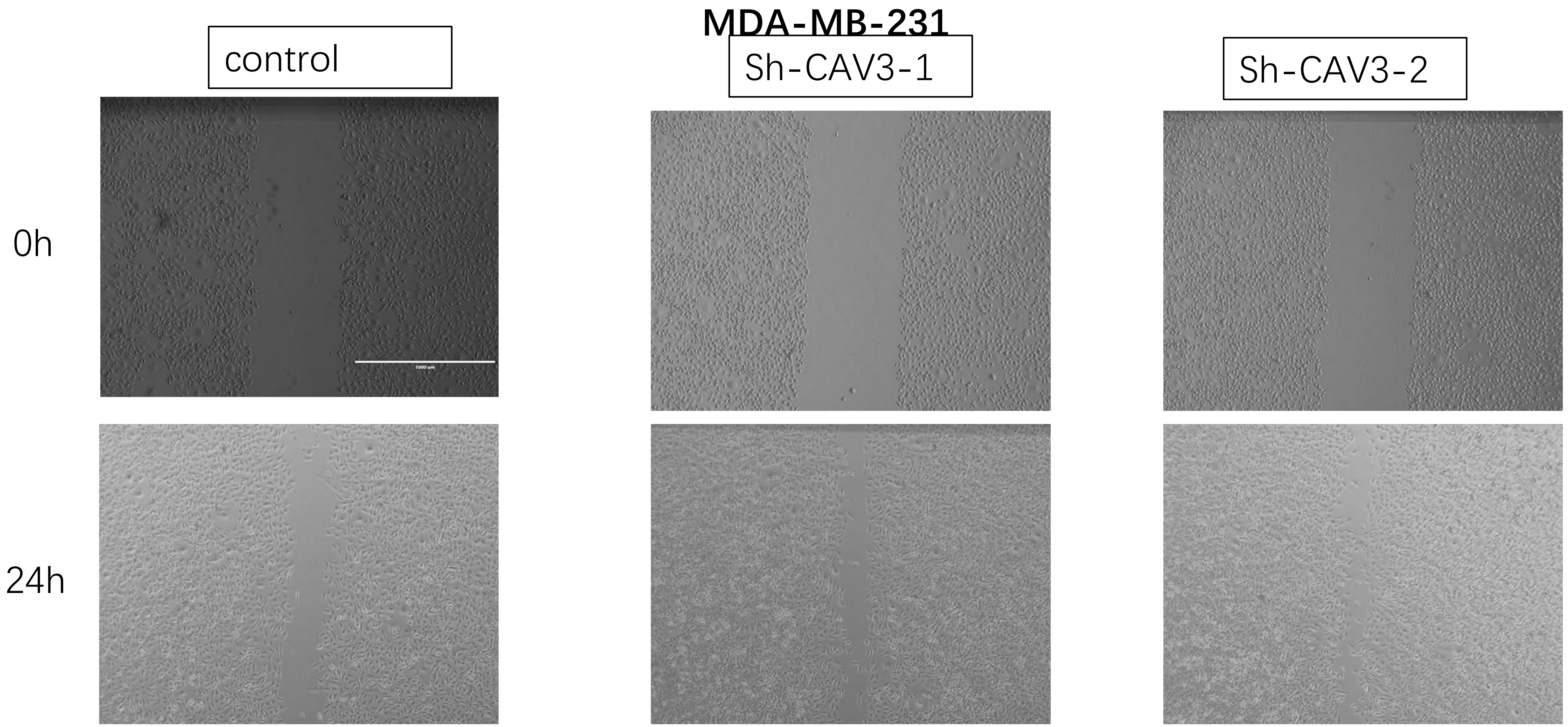

Figure 11 C

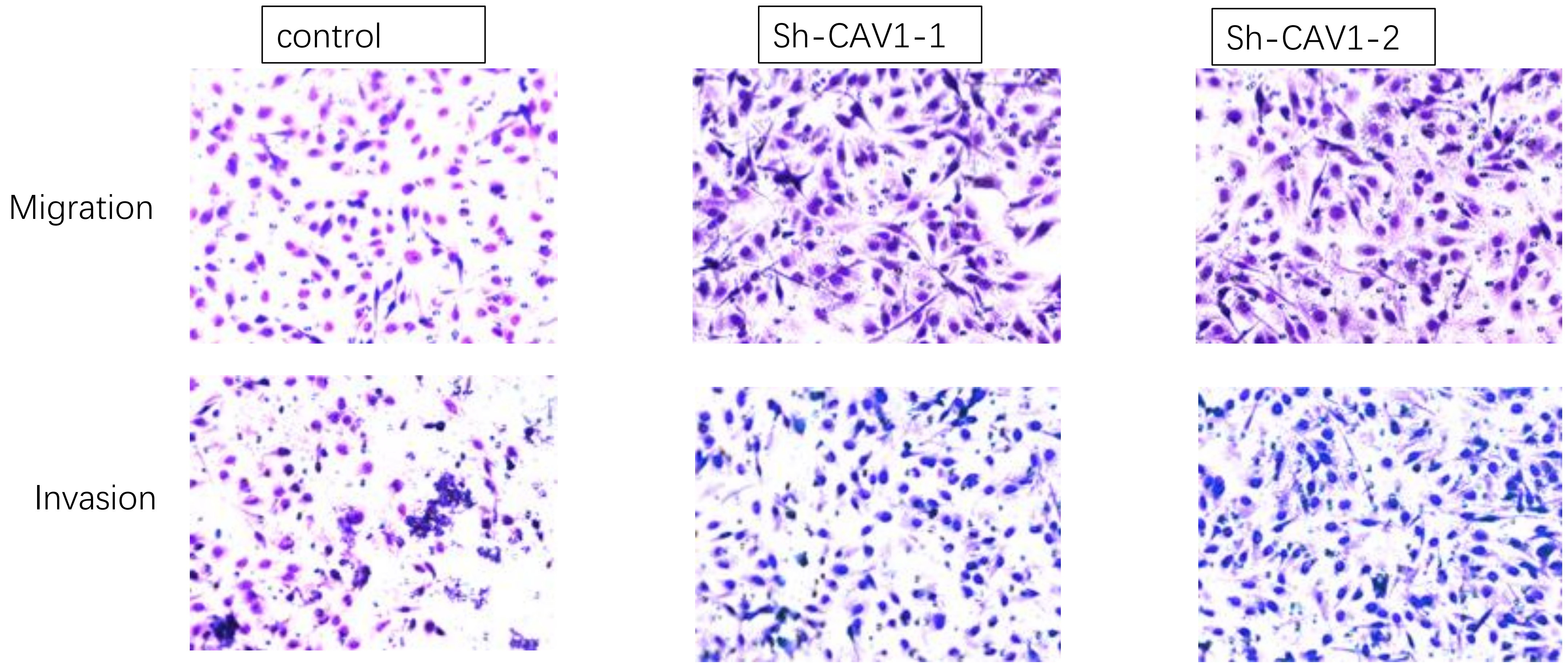

Figure 11 C

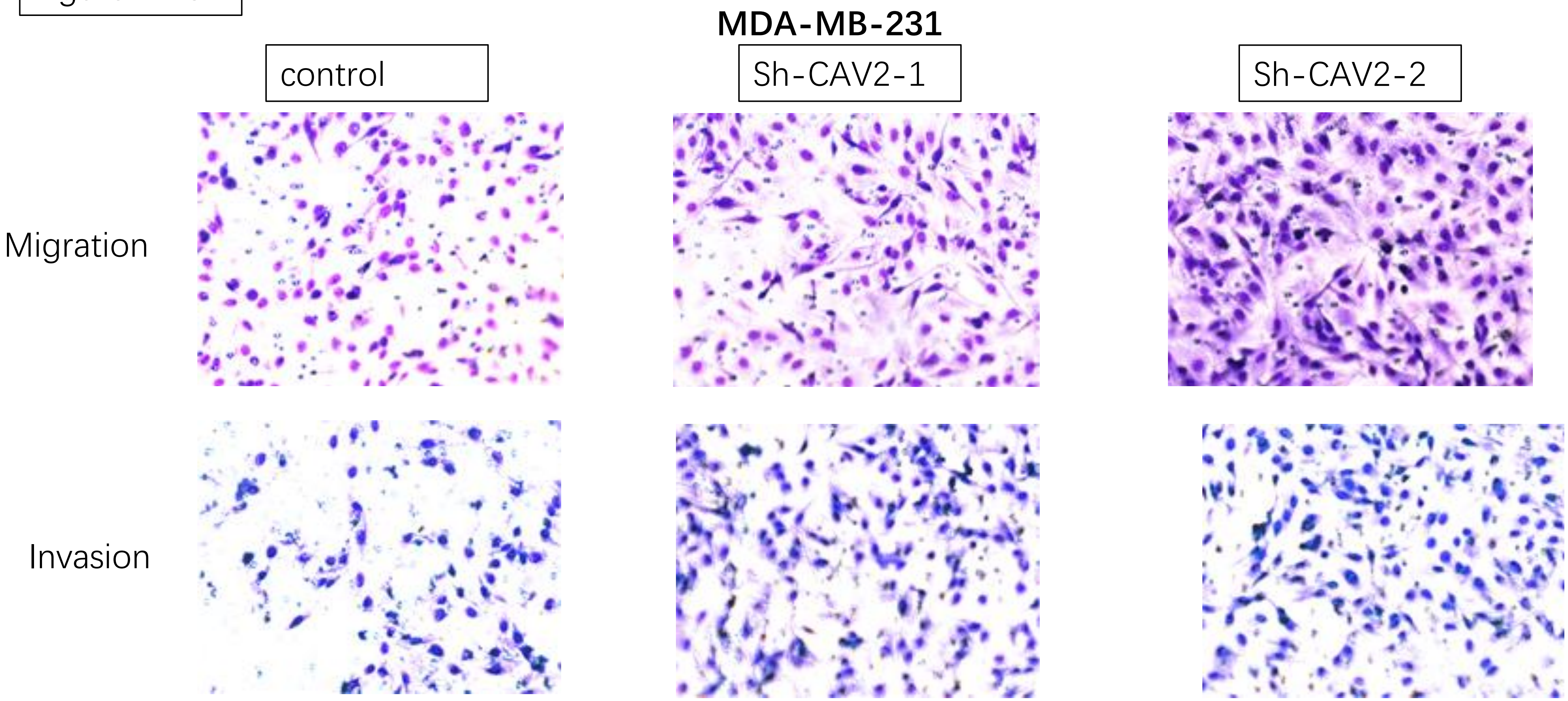

Figure 11 C

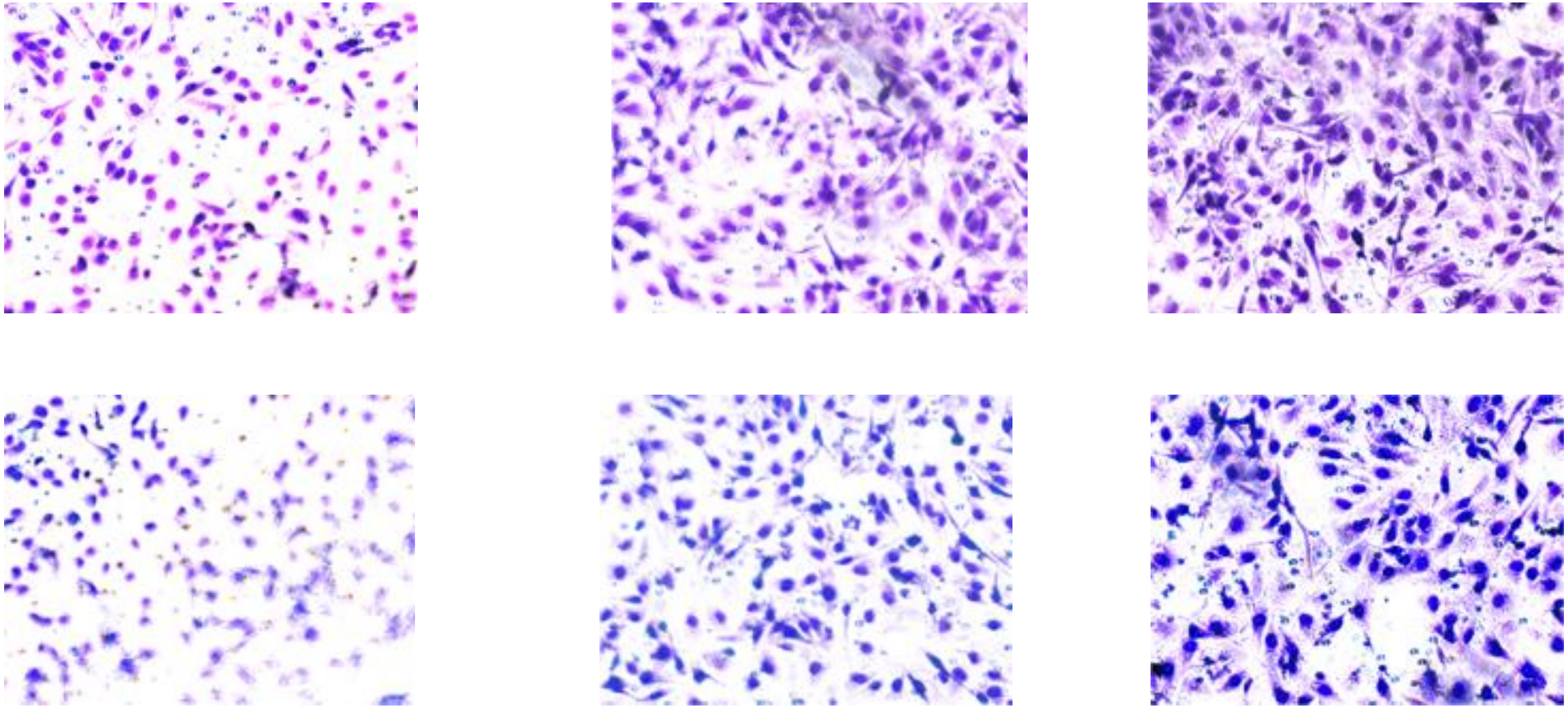

Figure 12 A

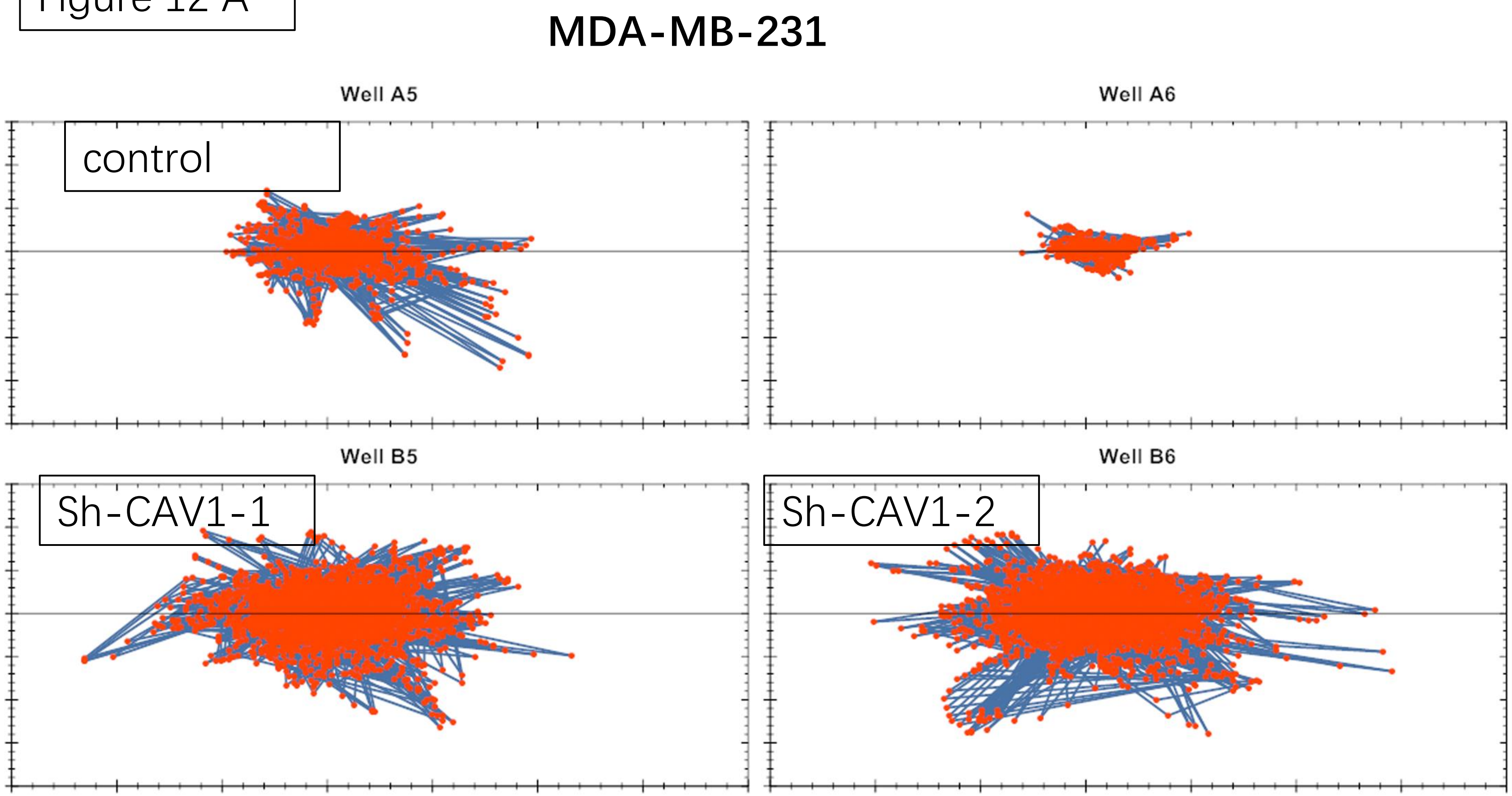
MDA-MB-231
Well A5
control
Well A6
Well B5
Sh-CAV1-1
Well B6
Sh-CAV1-2

Figure 12 A

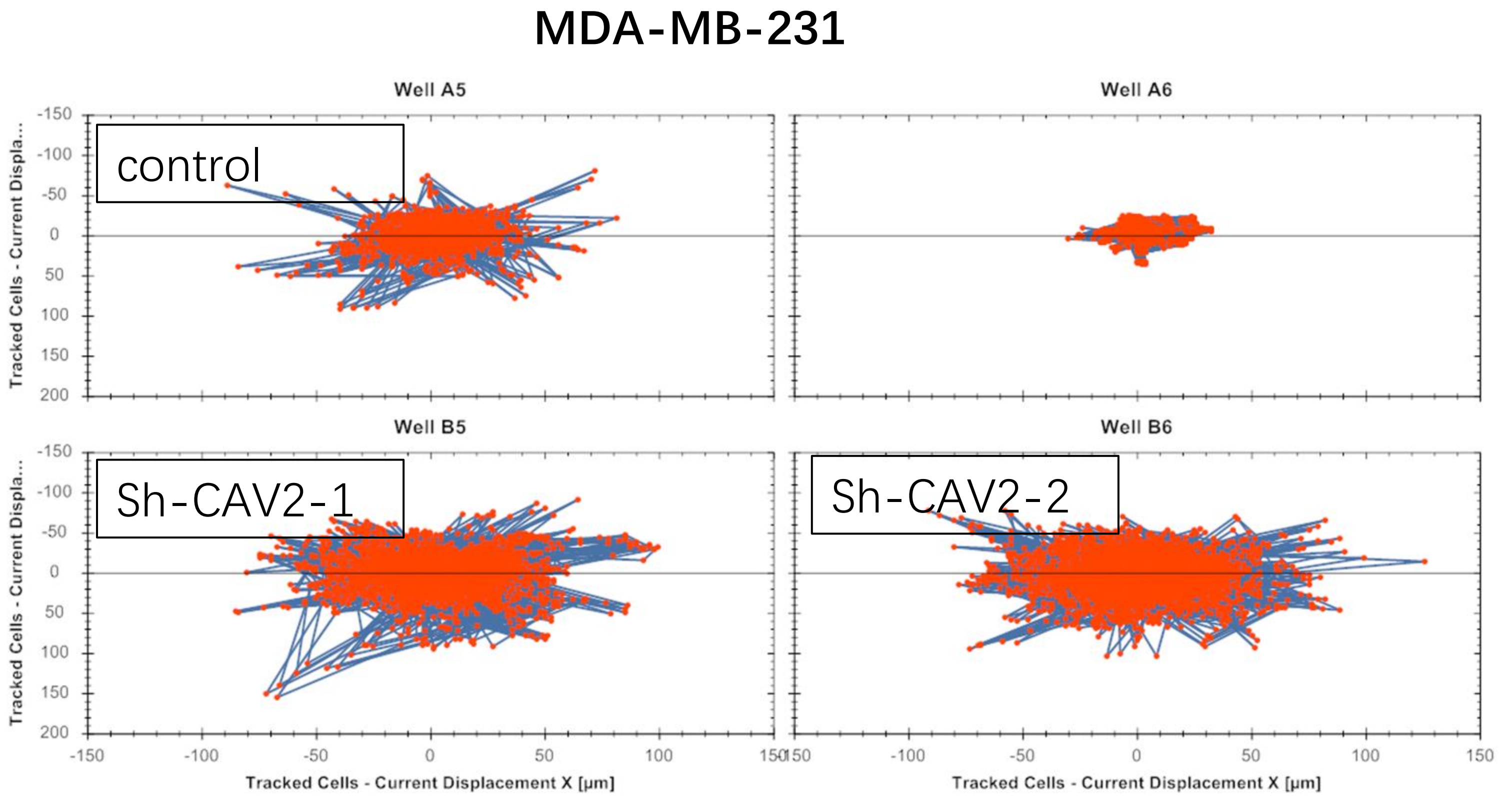
MDA-MB-231
Well A5
Well A6
Well B5
Well B6
control
Sh-CAV2-1
Sh-CAV2-2
Tracked Cells - Current Displa...
Tracked Cells - Current Displacement X [µm]
-150
-100
-50
0
50
100
150
200

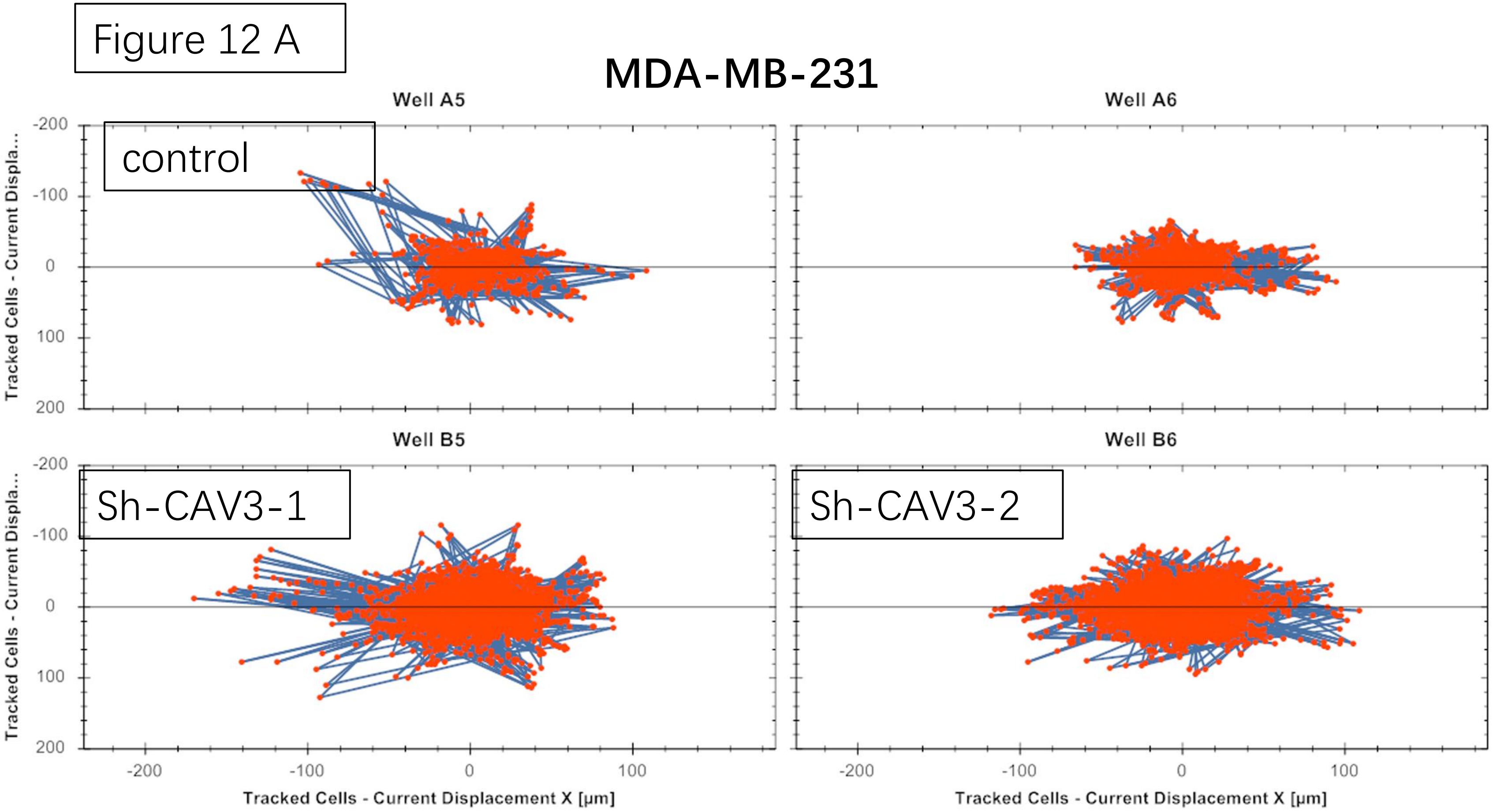
Figure 12 A
MDA-MB-231
Well A5
control
Well A6
Well B5
Sh-CAV3-1
Well B6
Sh-CAV3-2
Tracked Cells - Current Displa...
Tracked Cells - Current Displacement X [µm]
-200
-100
0
100
200

Figure 13 A

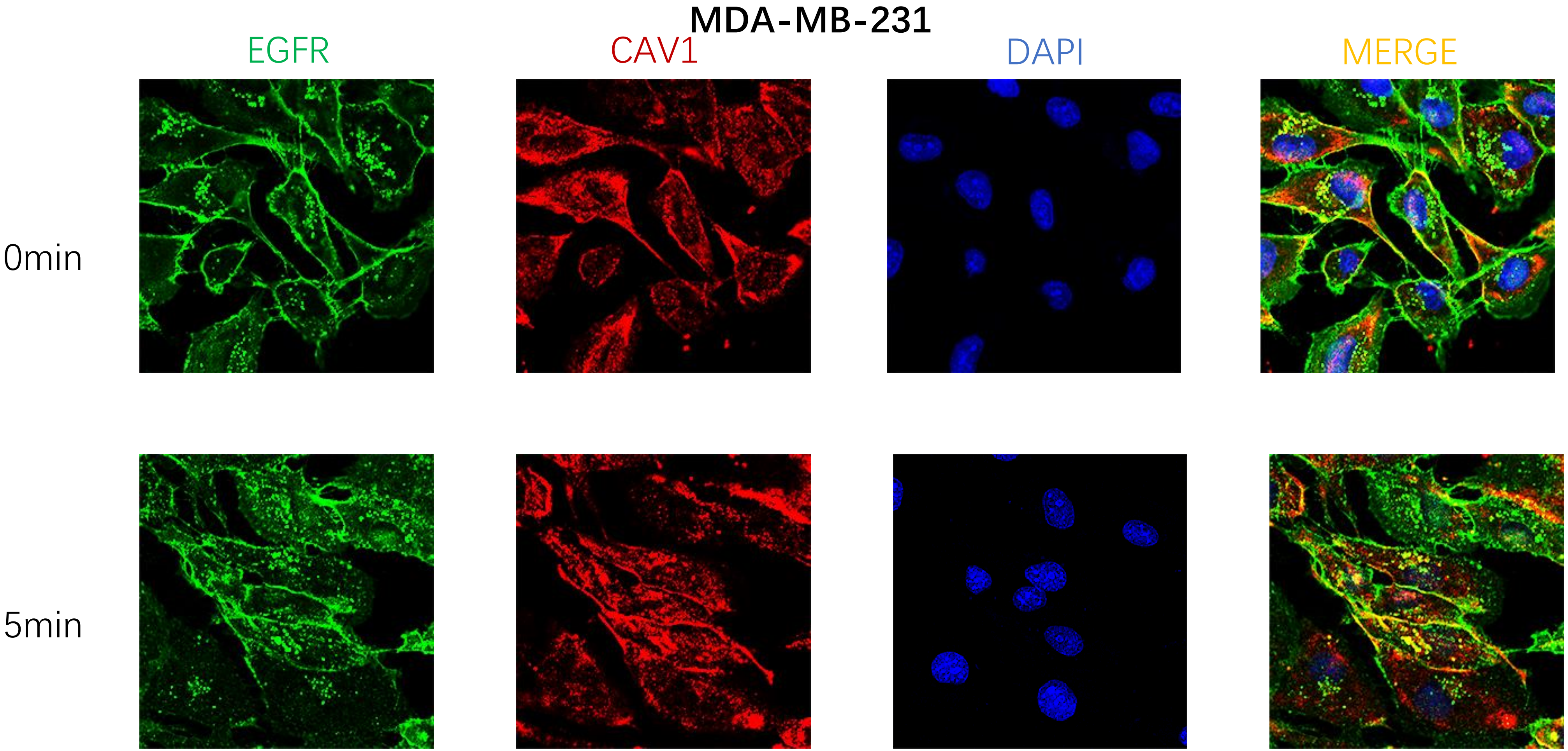

Figure 13 A

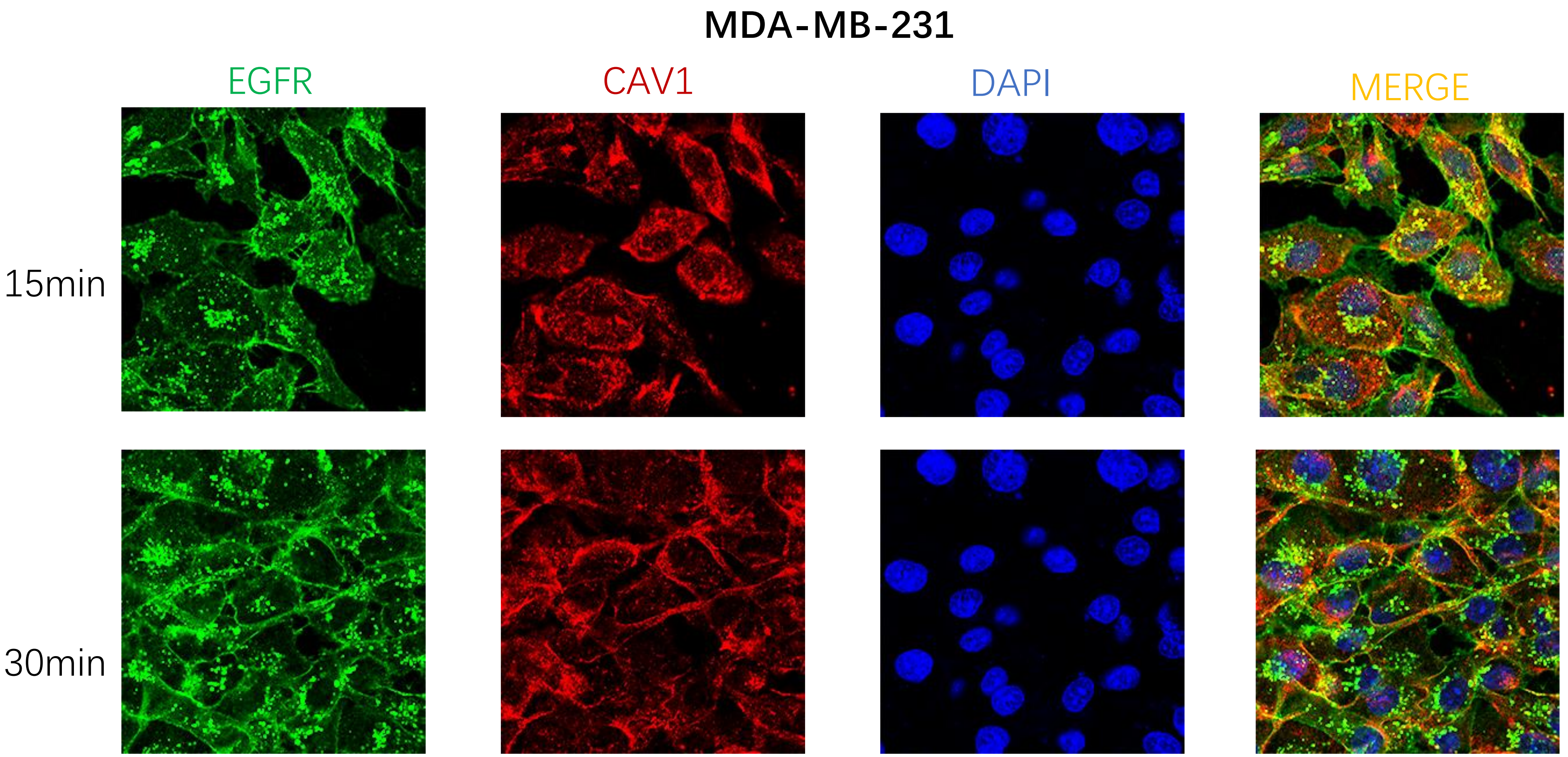

Figure 13 A

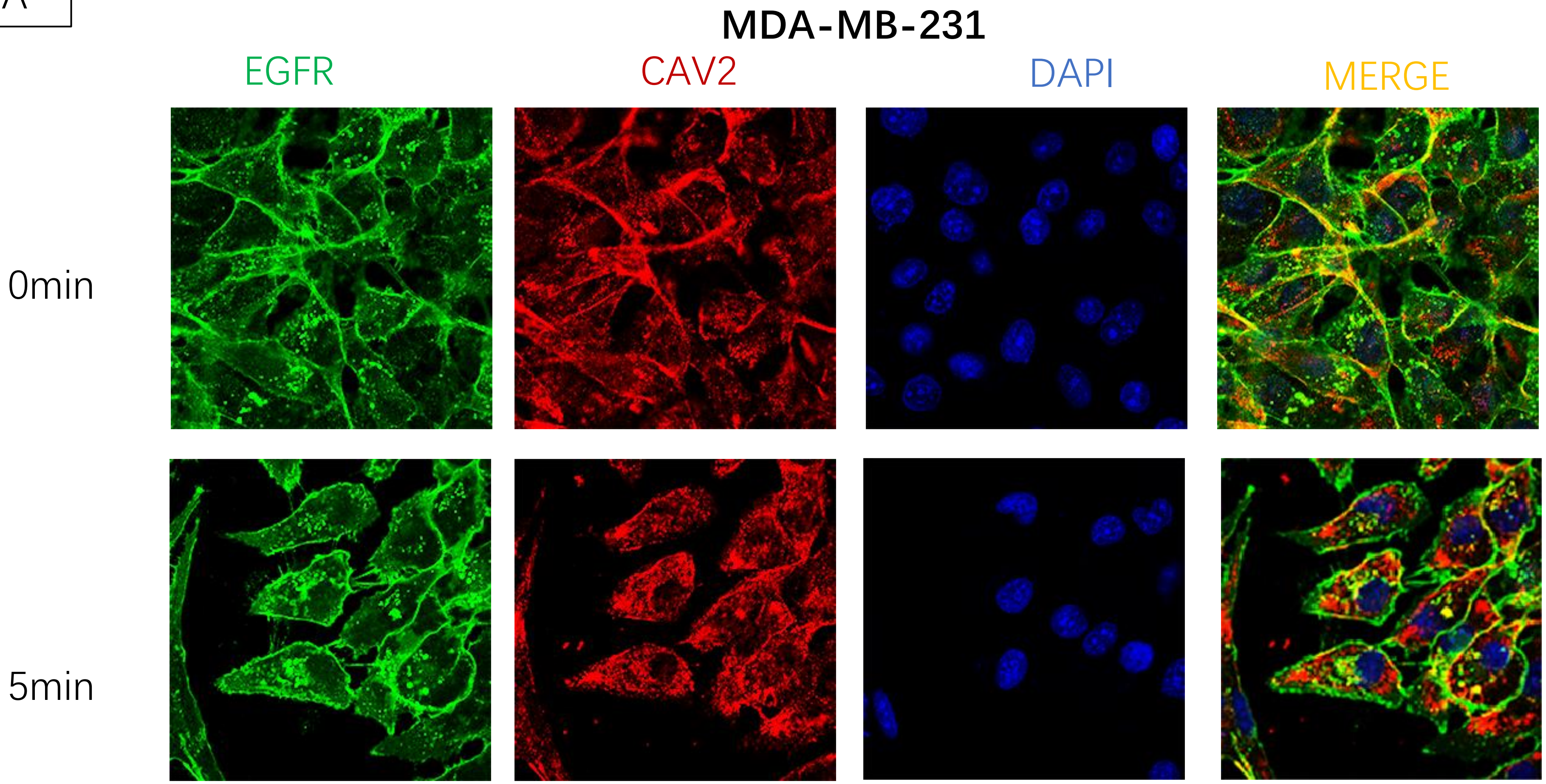

Figure 13 A

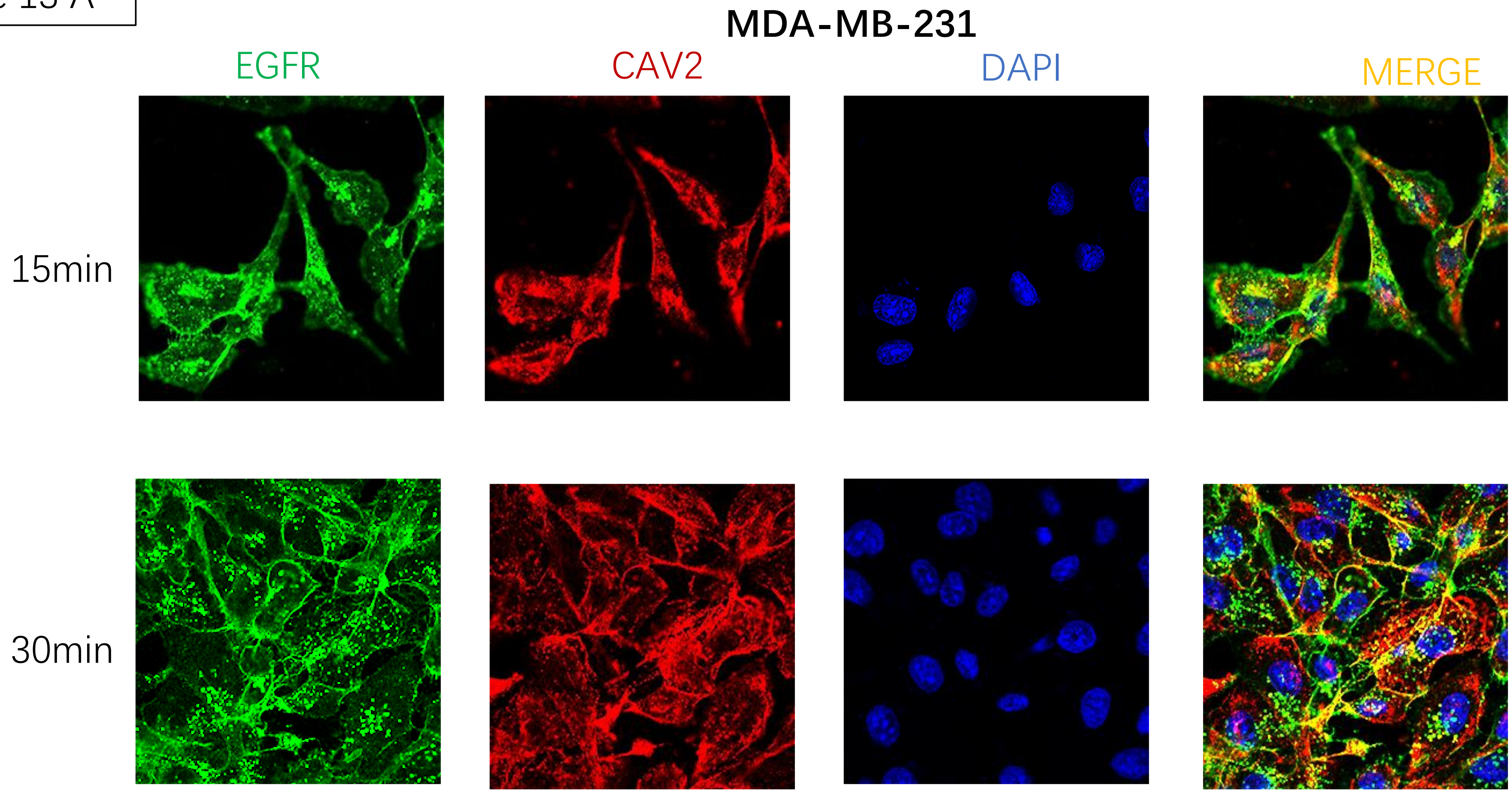

Figure 14 A

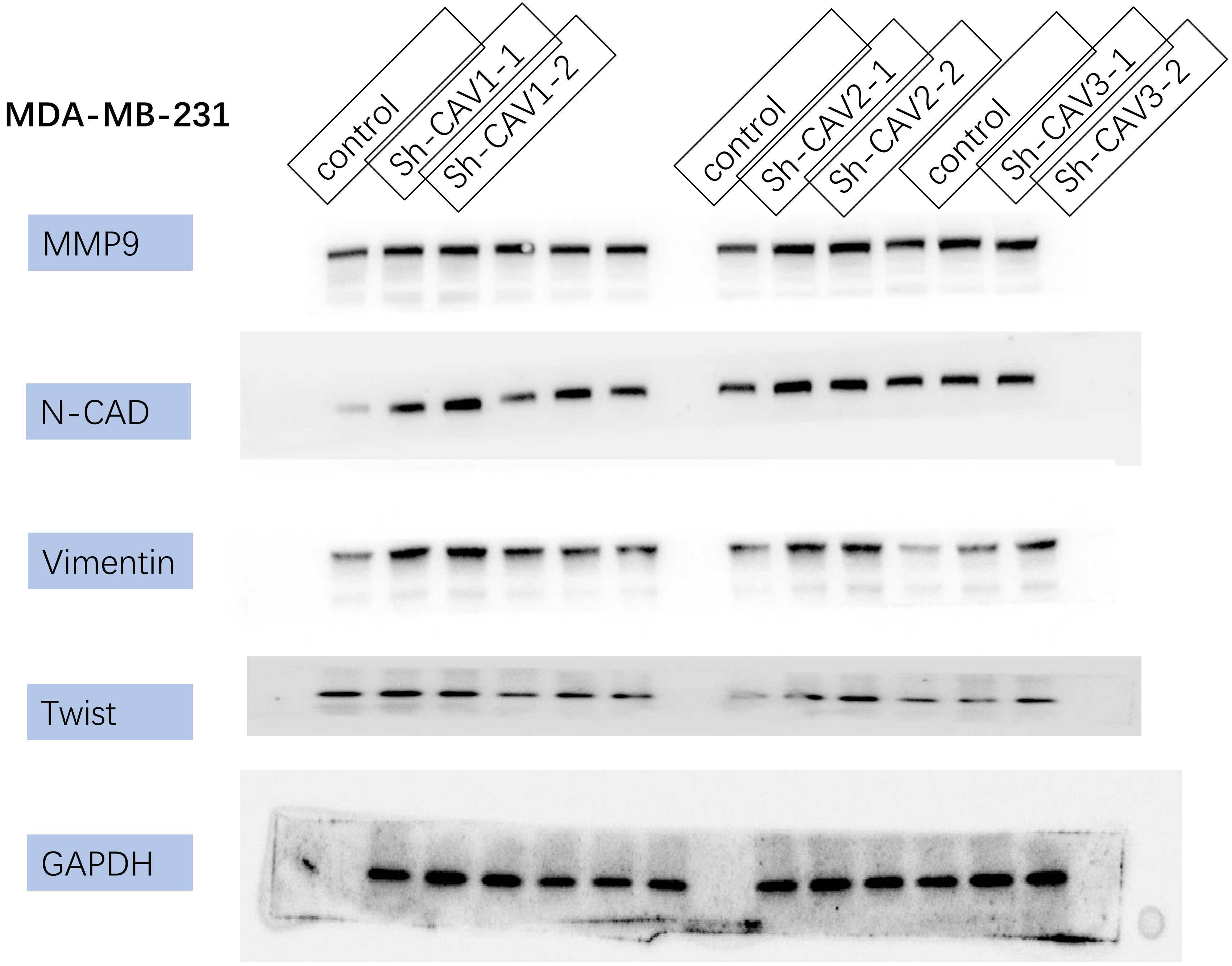
MDA-MB-231
control
Sh-CAV1-1
Sh-CAV1-2
control
Sh-CAV2-1
Sh-CAV2-2
control
Sh-CAV3-1
Sh-CAV3-2
MMP9
N-CAD
Vimentin
Twist
GAPDH

Figure 13 B

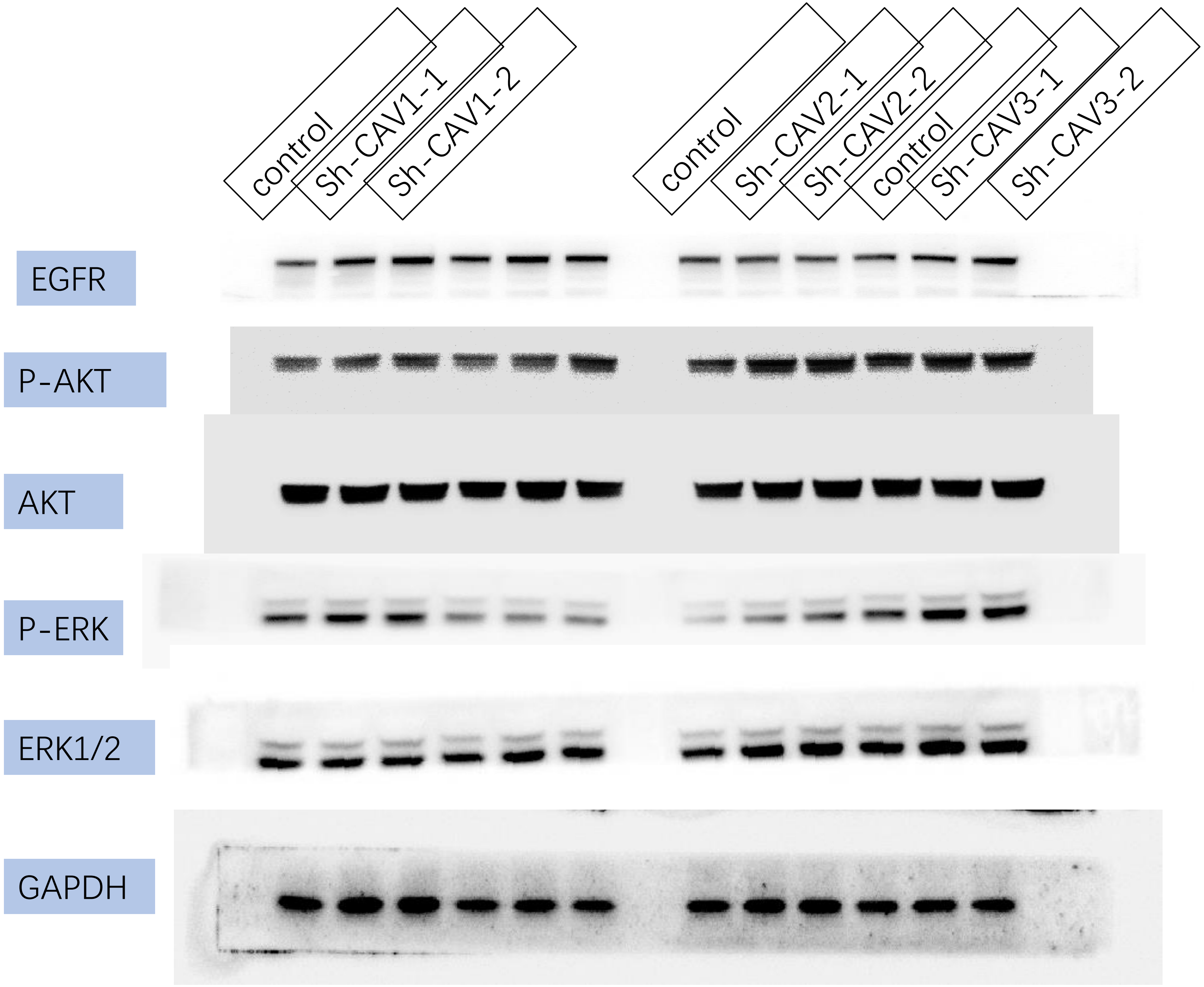
control
Sh-CAV1-1
Sh-CAV1-2
control
Sh-CAV2-1
Sh-CAV2-2
control
Sh-CAV3-1
Sh-CAV3-2
EGFR
P-AKT
AKT
P-ERK
ERK1/2
GAPDH
