# Supplementary material for: Identification and validation of a prognostic risk model based on caveolin family genes for breast cancer
Source: Front Cell Dev Biol. 2022 Sep 6;10:822187. doi: 10.3389/fcell.2022.822187 (PMC9485841; doi:10.3389/fcell.2022.822187)
Supplement: Supplementary file 2 [file Table1.docx]

| **Gene symbol** | **CpG Name** | **Hazard ratio** | **CI** | **P value** | **UCSC RefGene Group** | **Relation to UCSC CpG Island** |
| --- | --- | --- | --- | --- | --- | --- |
| **CAV1** | cg01265597 | 0.553 | (0.371;0.823) | 0.0046 | TSS1500 | N_Shore |
|  | cg18329349 | 0.597 | (0.394;0.906) | 0.019 | 5'UTR;1stExon | Island |
|  | cg17469978 | 0.659 | (0.442;0.982) | 0.038 | TSS200 | Island |
|  | cg04474049 | 0.579 | (0.339;0.988) | 0.033 | TSS1500 | N_Shore |
|  |  |  |  |  |  |  |
| **CAV2** | cg12739419 | 0.5 | (0.336;0.743) | 0.00051 | Body | S_Shore |
|  | cg16260298 | 0.622 | (0.419;0.922) | 0.017 | Body | Island |
|  | cg04696780 | 1.562 | (1.055;2.312) | 0.027 | TSS200 | N_Shore |
|  | cg16553024 | 0.549 | (0.322;0.936) | 0.018 | TSS1500 | N_Shore |
|  |  |  |  |  |  |  |
| **CAV3** | cg16328896 | 0.546 | (0.365;0.817) | 0.0027 | TSS200 | Open_Sea |
|  | cg16448890 | 0.651 | (0.438;0.968) | 0.032 | Body | Open_Sea |

**Table 1.** **The significant prognostic values of CpG in the Cavs family members**
