# Supplementary figures and images for: Identification and validation of a prognostic risk model based on caveolin family genes for breast cancer
Source: Front Cell Dev Biol. 2022 Sep 6;10:822187. doi: 10.3389/fcell.2022.822187 (PMC9485841; doi:10.3389/fcell.2022.822187)

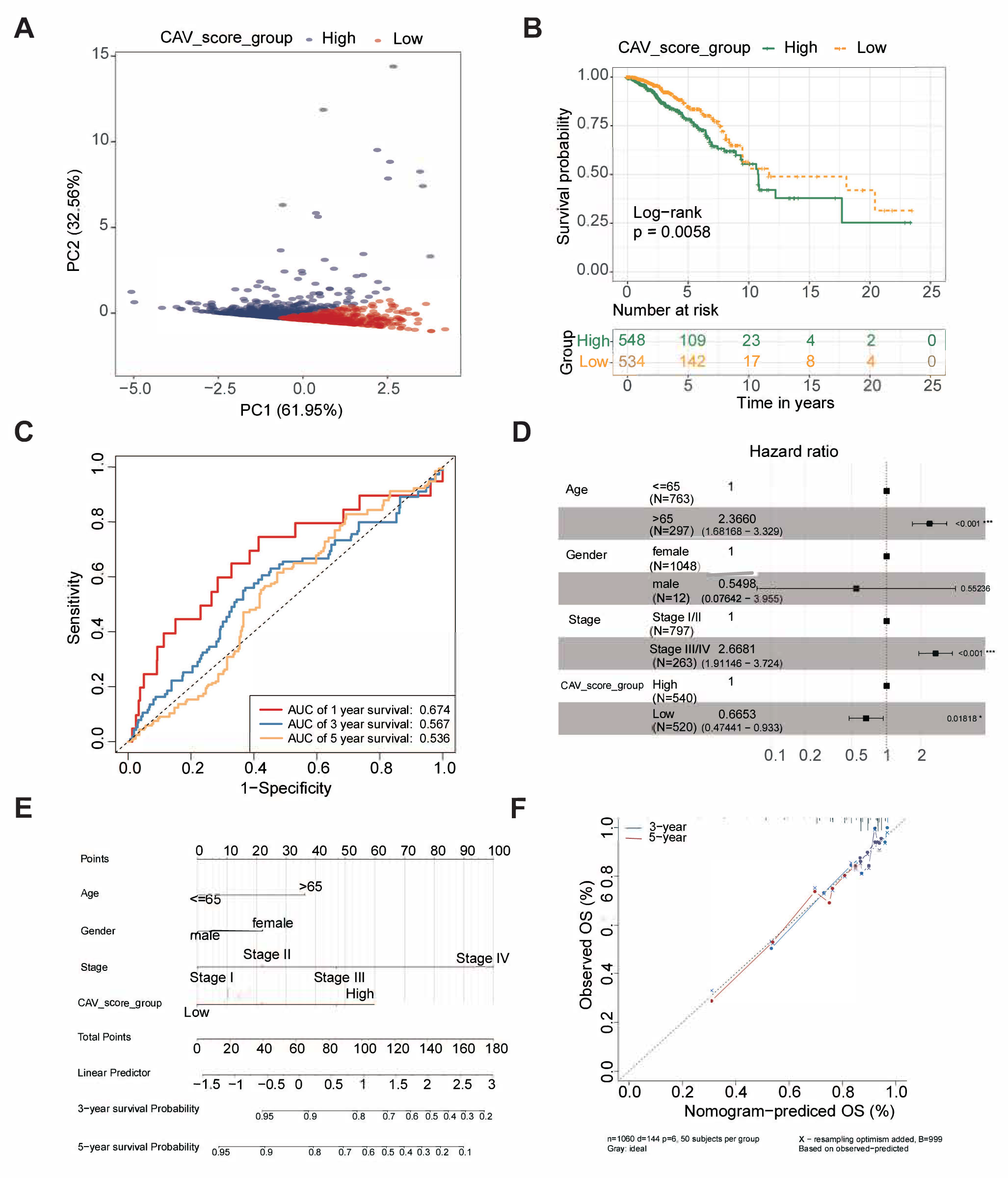

Supplement: Supplementary file 3 [file DataSheet1.zip › Fig6/Figure 6.tif]

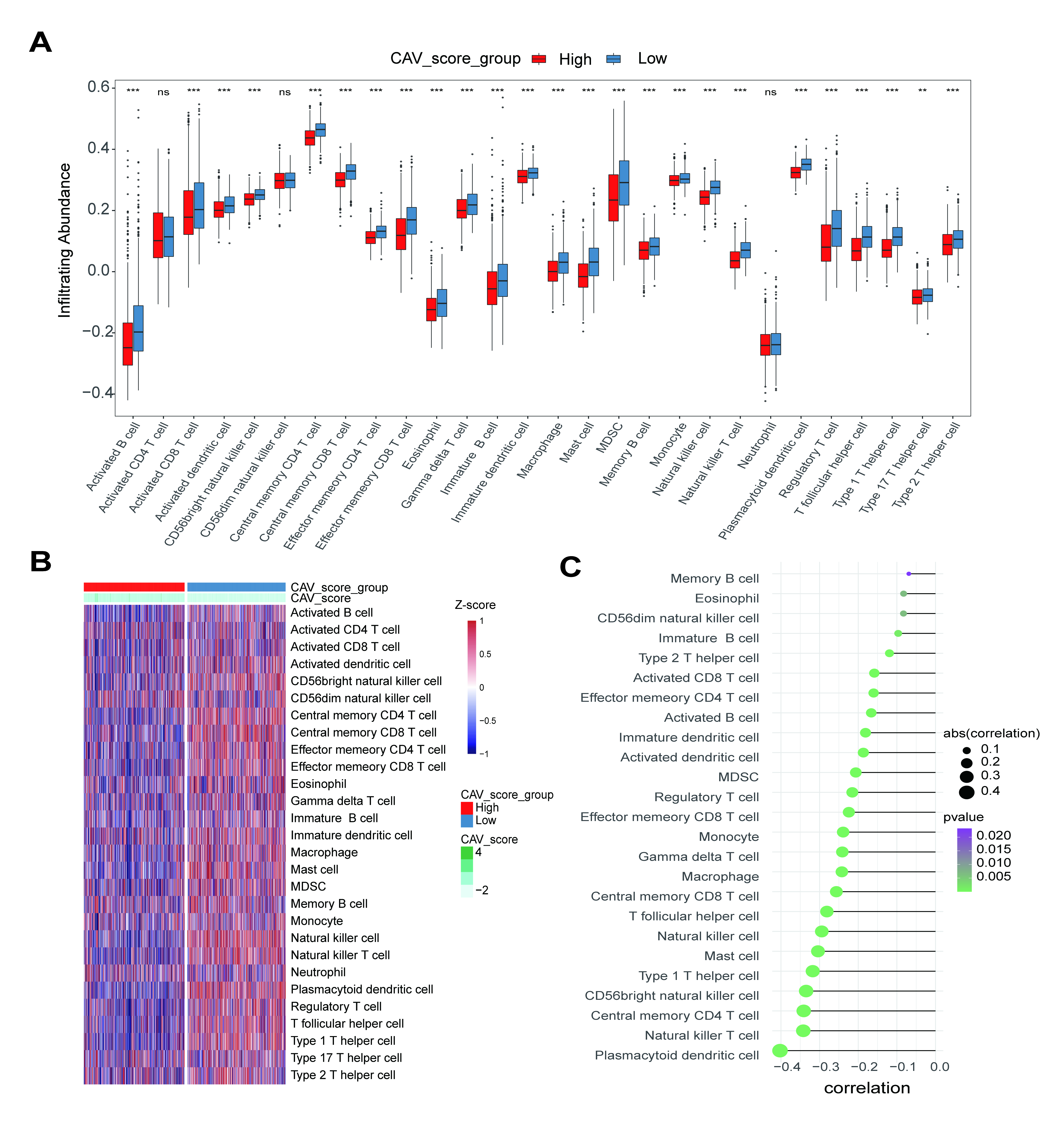

Supplement: Supplementary file 3 [file DataSheet1.zip › Fig7/Figure 7.tif]

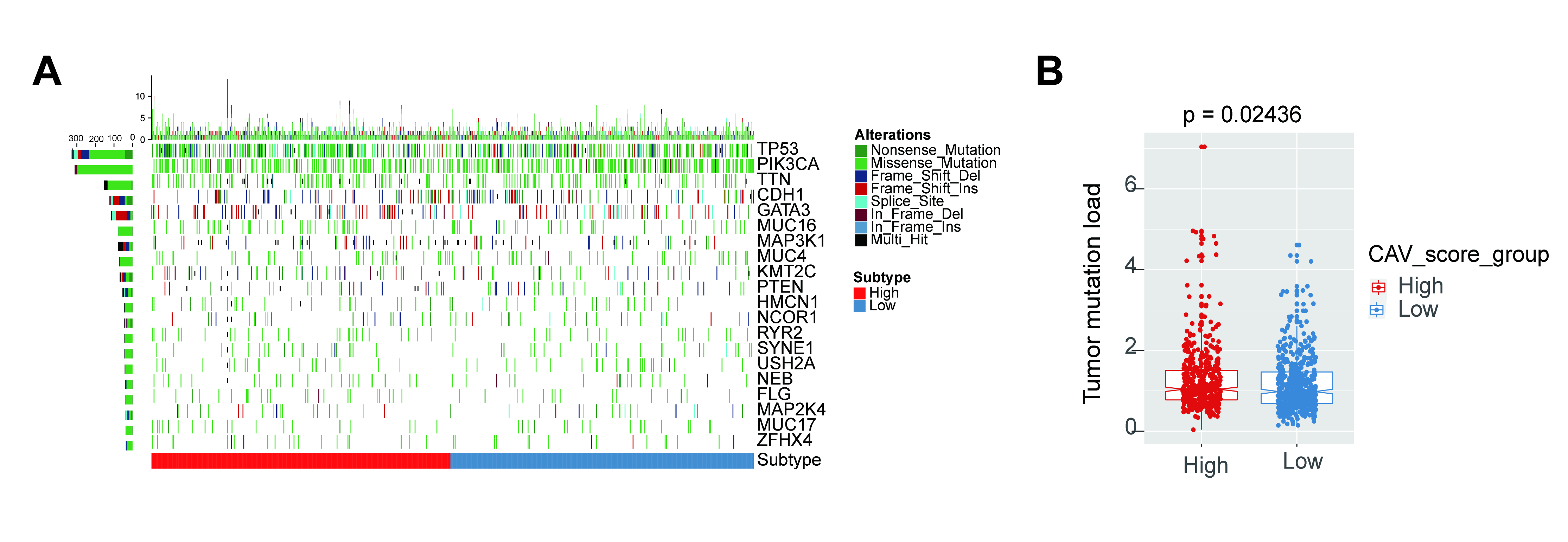

Supplement: Supplementary file 3 [file DataSheet1.zip › Fig8/Figure 8.tif]

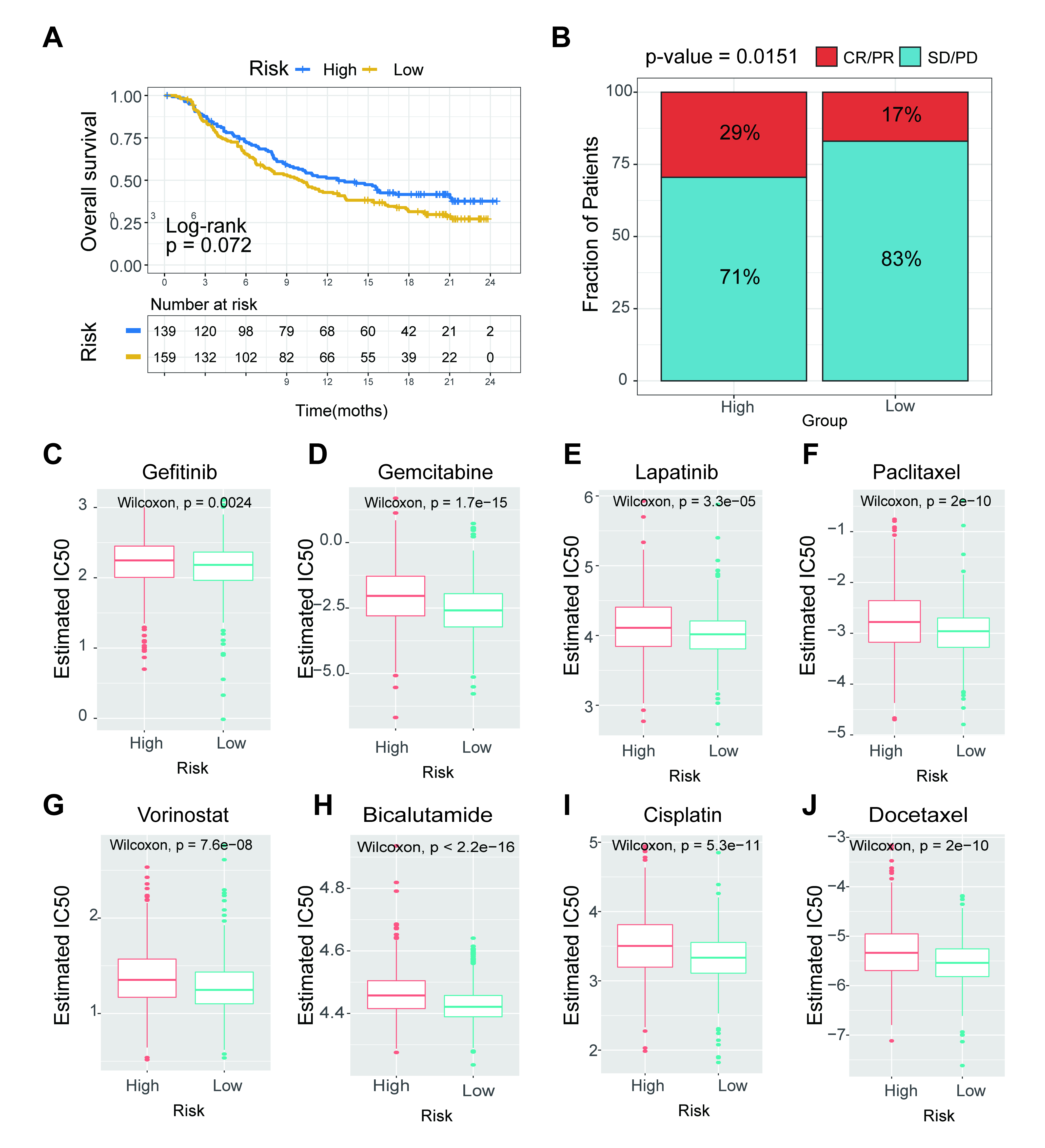

Supplement: Supplementary file 3 [file DataSheet1.zip › FIg9/Figure 9.tif]

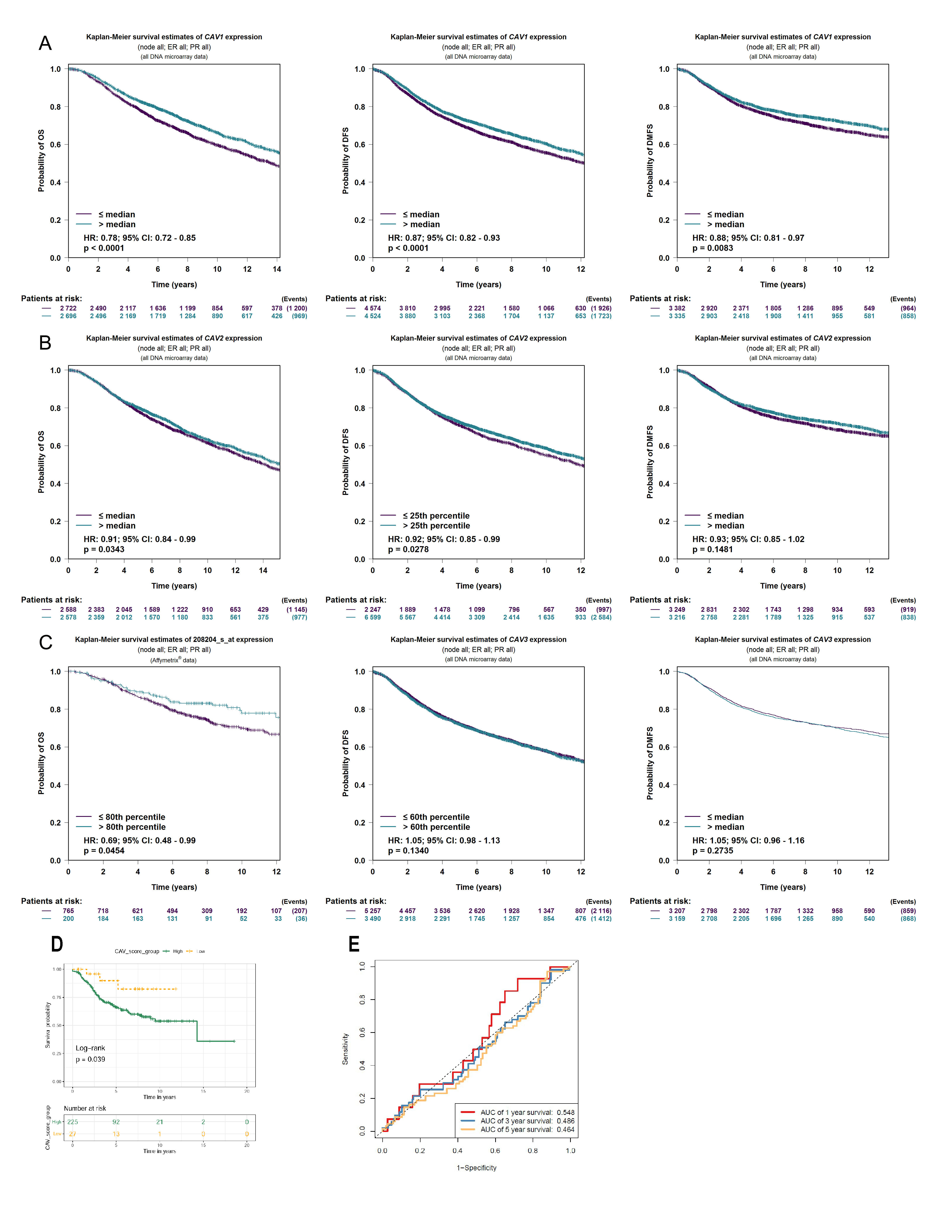

Supplement: Supplementary file 5 [file Image1.jpg]
