# Supplementary material for: Identification and validation of a prognostic risk model based on caveolin family genes for breast cancer
Source: Front Cell Dev Biol. 2022 Sep 6;10:822187. doi: 10.3389/fcell.2022.822187 (PMC9485841; doi:10.3389/fcell.2022.822187)
Supplement: Supplementary file 4 [file Table2.docx]

| **Gene symbol** | |  | | **Sequence 1** | | **Sequence 2** |  |
| --- | --- | --- | --- | --- | --- | --- | --- |
| **CAV1** | **Forward** | | CCGGCCACCTTCACTGTGACGAAATCTCGAGATTTCGTCACAGTGAAGGTGGTTTTTG | | CCGGGCTTTGTGATTCAATCTGTAACTCGAGTTACAGATTGAATCACAAAGCTTTTTG | | |
|  | **Reverse** | | AATTCAAAAACCACCTTCACTGTGACGAAATCTCGAGATTTCGTCACAGTGAAGGTGG | | AATTCAAAAAGCTTTGTGATTCAATCTGTAACTCGAGTTACAGATTGAATCACAAAGC | | |
| **CAV2** | **Forward** | | CCGGCAACTGAGCCAGGATTGAATACTCGAGTATTCAATCCTGGCTCAGTTGTTTTTG | | CCGGGCACCACTGTTCTGTTCATTTCTCGAGAAATGAACAGAACAGTGGTGCTTTTTG | | |
|  | **Reverse** | | AATTCAAAAACAACTGAGCCAGGATTGAATACTCGAGTATTCAATCCTGGCTCAGTTG | | AATTCAAAAAGCACCACTGTTCTGTTCATTTCTCGAGAAATGAACAGAACAGTGGTGC | | |
| **CAV3** | **Forward** | | CCGGCGTCAAGGATATCCACTGCAACTCGAGTTGCAGTGGATATCCTTGACGTTTTTG | | CCGGCAAGAACATTAACGAGGACATCTCGAGATGTCCTCGTTAATGTTCTTGTTTTTG | | |
|  | **Reverse** | | AATTCAAAAACGTCAAGGATATCCACTGCAACTCGAGTTGCAGTGGATATCCTTGACG | | AATTCAAAAACAAGAACATTAACGAGGACATCTCGAGATGTCCTCGTTAATGTTCTTG | | |

**Supplemental Table 1.** **The sequences used for gene silencing of CAVs.**
